# Single nucleus RNA-sequencing integrated into risk variant colocalization discovers 17 cell-type-specific abdominal obesity genes for metabolic dysfunction-associated steatotic liver disease

**DOI:** 10.1016/j.ebiom.2024.105232

**Published:** 2024-07-10

**Authors:** Seung Hyuk T. Lee, Kristina M. Garske, Uma Thanigai Arasu, Asha Kar, Zong Miao, Marcus Alvarez, Amogha Koka, Nicholas Darci-Maher, Jihane N. Benhammou, David Z. Pan, Tiit Örd, Dorota Kaminska, Ville Männistö, Sini Heinonen, Martin Wabitsch, Markku Laakso, Vatche G. Agopian, Joseph R. Pisegna, Kirsi H. Pietiläinen, Jussi Pihlajamäki, Minna U. Kaikkonen, Päivi Pajukanta

**Affiliations:** a Department of Human Genetics, David Geffen School of Medicine at UCLA, Los Angeles, CA, USA; b A. I. Virtanen Institute for Molecular Sciences, University of Eastern Finland, Kuopio, Finland; c Bioinformatics Interdepartmental Program, UCLA, Los Angeles, CA, USA; d Vatche and Tamar Manoukian Division of Digestive Diseases and Gastroenterology, Hepatology and Parenteral Nutrition, David Geffen School of Medicine at UCLA and VA Greater Los Angeles HCS, Los Angeles, CA, USA; e Institute of Public Health and Clinical Nutrition, University of Eastern Finland, Kuopio, Finland; f Division of Cardiology, Department of Medicine, UCLA, Los Angeles, CA, USA; g Institute of Clinical Medicine, Internal Medicine, University of Eastern Finland, Kuopio, Finland; h Department of Internal Medicine, Kuopio University Hospital, Kuopio, Finland; i Obesity Research Unit, Research Program for Clinical and Molecular Metabolism, Faculty of Medicine, University of Helsinki, Helsinki, Finland; j Division of Pediatric Endocrinology and Diabetes, Department of Pediatrics and Adolescent Medicine, University of Ulm, Ulm, Germany; k Department of Surgery, David Geffen School of Medicine at UCLA, Los Angeles, CA, USA; l Department of Medicine and Human Genetics, Division of Gastroenterology, Hepatology and Parenteral Nutrition, David Geffen School of Medicine at UCLA and VA Greater Los Angeles HCS, Los Angeles, CA, USA; m Healthy WeightHub, Endocrinology, Abdominal Center, Helsinki University Central Hospital and University of Helsinki, Helsinki, Finland; n Department of Medicine, Endocrinology and Clinical Nutrition, Kuopio University Hospital, Kuopio, Finland; o Institute for Precision Health, David Geffen School of Medicine at UCLA, Los Angeles, CA, USA

**Keywords:** Abdominal obesity, Metabolic dysfunction-associated steatotic liver disease (MASLD), Single-nucleus RNA-sequencing (snRNA-seq), Colocalization, Expression quantitative trait loci (eQTL), Genome-wide association study (GWAS), Waist-hip ratio adjusted for body mass index (WHRadjBMI)

## Abstract

**Background:**

Abdominal obesity increases the risk for non-alcoholic fatty liver disease (NAFLD), now known as metabolic dysfunction-associated steatotic liver disease (MASLD).

**Methods:**

To elucidate the directional cell-type level biological mechanisms underlying the association between abdominal obesity and MASLD, we integrated adipose and liver single nucleus RNA-sequencing and bulk *cis*-expression quantitative trait locus (eQTL) data with the UK Biobank genome-wide association study (GWAS) data using colocalization. Then we used colocalized *cis*-eQTL variants as instrumental variables in Mendelian randomization (MR) analyses, followed by functional validation experiments on the target genes of the *cis*-eQTL variants.

**Findings:**

We identified 17 colocalized abdominal obesity GWAS variants, regulating 17 adipose cell-type marker genes. Incorporating these 17 variants into MR discovers a putative tissue-of-origin, cell-type-aware causal effect of abdominal obesity on MASLD consistently with multiple MR methods without significant evidence for pleiotropy or heterogeneity. Single cell data confirm the adipocyte-enriched mean expression of the 17 genes. Our cellular experiments across human adipogenesis identify risk variant -specific epigenetic and transcriptional mechanisms. Knocking down two of the 17 genes, *PPP2R5A* and *SH3PXD2B*, shows a marked decrease in adipocyte lipidation and significantly alters adipocyte function and adipogenesis regulator genes, including *DGAT2*, *LPL, ADIPOQ*, *PPARG,* and *SREBF1*. Furthermore, the 17 genes capture a characteristic MASLD expression signature in subcutaneous adipose tissue.

**Interpretation:**

Overall, we discover a significant cell-type level effect of abdominal obesity on MASLD and trace its biological effect to adipogenesis.

**Funding:**

10.13039/100000002NIH grants R01HG010505, R01DK132775, and R01HL170604; the 10.13039/501100000781European Research Council (ERC) under the 10.13039/501100007601European Union’s Horizon 2020 research and innovation program (Grant No. 802825), 10.13039/501100002341Academy of Finland (Grants Nos. 333021), the 10.13039/501100005633Finnish Foundation for Cardiovascular Research the 10.13039/501100006306Sigrid Jusélius Foundation and the 10.13039/501100004012Jane and Aatos Erkko Foundation; 10.13039/100005347American Association for the Study of Liver Diseases (AASLD) Advanced Transplant Hepatology award and NIH/NIDDK (P30DK41301) Pilot and Feasibility award; NIH/NIEHS F32 award (F32ES034668); 10.13039/501100013500Finnish Diabetes Research Foundation, Kuopio University Hospital Project grant (EVO/VTR grants 2005-2021), the 10.13039/501100002341Academy of Finland grant (Contract no. 138006); Academy of Finland (Grant Nos 335443, 314383, 272376 and 266286), Sigrid Jusélius Foundation, Finnish Medical Foundation, 10.13039/501100013500Finnish Diabetes Research Foundation, 10.13039/501100009708Novo Nordisk Foundation (#NNF20OC0060547, NNF17OC0027232, NNF10OC1013354) and Government Research Funds to Helsinki University Hospital; 10.13039/501100007083Orion Research Foundation, 10.13039/100010129Maud Kuistila Foundation, Finish Medical Foundation, and 10.13039/100007797University of Helsinki.

**Research in context**

**Evidence before this study**

Previous genome-wide association studies (GWASs) have identified abdominal obesity loci that harbor genes predominantly expressed in the adipose tissue, suggesting that adipose tissue is a central tissue of action for obesity risk variants. Obesity induced dysfunction of adipose tissue is hypothesised to lead to metabolic dysfunction-associated steatotic liver disease (MASLD). However, adipose cell-type level biological mechanisms underlying the effects of abdominal obesity on MASLD remain elusive. To assess the existing understanding of this topic, we searched PubMed until May 15, 2024 with the following terms: (NAFLD) OR (MASLD) AND (abdominal obesity) OR (WHRadjBMI) AND (colocalization) OR (Mendelian randomization). This search returned 4 articles. Of these 4 studies, none utilised snRNA-seq data and colocalization and Mendelian randomization methods to discover adipose cell-type level effect of abdominal obesity on MASLD.

**Added value of this study**

We conducted a multi-omics analysis integrating subcutaneous adipose single nucleus RNA-sequencing and *cis*-expression quantitative trait locus (eQTL) data colocalized with waist-hip ratio adjusted for body mass index (WHRadjBMI) GWAS data, which identified 17 adipose cell-type-specific abdominal obesity genes. Using the colocalized regional variants of the 17 genes as instrumental variables, we demonstrate a putative causal effect of abdominal obesity on MASLD through Mendelian randomization analyses. We further show that the abdominal obesity genes that are highly expressed in preadipocytes and adipocytes are differentially expressed longitudinally during human preadipocyte differentiation (i.e. adipogenesis), critical for healthy adipose tissue. Finally, we found that knockdown of two of these genes, *PPP2R5A* and *SH3PXD2B*, in human preadipocytes impairs adipogenesis. Our findings discover 17 genes, many with links to human adipogenesis, that underly the significant adipose cell-type level effect of abdominal obesity on MASLD.

**Implications of all the available evidence**

This study discovers 17 cell-type-specific abdominal obesity genes with an adipose-origin MASLD expression signature. These genes may serve as potential therapeutic targets for future treatment of abdominal obesity, which in turn can prevent obesity-driven MASLD. Additional functional studies with these genes will further elucidate their underlying molecular mechanisms with cross tissue effects.

## Introduction

Obesity is a fast-increasing global health problem^1^ that predisposes to multiple common diseases, including non-alcoholic fatty liver disease (NAFLD), now known as metabolic dysfunction-associated steatotic liver disease (MASLD).^2^ Abdominal obesity, in particular, has been linked to these cardiometabolic co-morbidities. Waist-to-hip ratio adjusted for body mass index (WHRadjBMI) is a well-established surrogate for abdominal obesity^3^ and has a heritability estimate of 40–80%.^4^ Previous extensive genome-wide association studies (GWASs) have identified numerous WHRadjBMI-associated loci^4^ and showed that the genes at the WHRadjBMI GWAS loci are predominantly expressed in the adipose tissue,^5^^,^^6^ whereas the body mass index (BMI) GWAS loci harbor genes with expression preference in brain.^5^ This suggests that adipose tissue pathways contribute to the cardiometabolically relevant abdominal obesity (WHRadjBMI) as opposed to the traditionally used obesity proxy, BMI. However, the causal cell-type level biological mechanisms at many of the obesity GWAS loci remain elusive.

A large number of GWAS variants reside in non-coding regions of the genome and are thus likely gene regulatory.^7^ Recently, genetic colocalization analysis, which statistically assesses overlap between GWAS and *cis*-expression quantitative trait loci (eQTL), has been widely used to elucidate biological mechanisms at GWAS loci. Colocalization analysis tests for the hypothesis that two traits, i.e. the GWAS and gene expression traits, share the same underlying variants. We hypothesised that colocalization of the WHRadjBMI GWAS variants and adipose *cis*-eQTL data can improve the identification of regional genes regulated by the GWAS variants, especially if adjusting the adipose gene expression data for the confounding cell-type proportions prior to the identification of *cis*-eQTLs. This rationale is supported by the fact that cellular heterogeneity in tissues, such as adipose tissue, confounds the *cis*-eQTL results,^8^ and thus hampers the discovery of biological mechanisms underlying abdominal obesity.

MASLD, the key abdominal obesity comorbidity, has a staggering global prevalence of 25% and spectrum of severity, ranging from simple steatosis to metabolic dysfunction-associated steatohepatitis (MASH).^9^^,^^10^ The known MASLD variants have provided insight into the aetiology of MASLD^11^; however, they explain only ∼10–20% of the overall heritability,^12^ warranting additional genetic discoveries. Using all WHRadjBMI GWAS variants as instrumental variables (IVs) in a Mendelian randomization (MR) analysis, past studies have shown a causal relationship between abdominal obesity and MASLD.^13^^,^^14^ However, there is a biological lack of knowledge about the tissue- and cell-type-of-origin effects underlying the impact of abdominal obesity on MASLD. Furthermore, the genetic signals of obesity exhibit only small effect sizes and are associated with many other confounders, making the conducted MR analysis prone to horizontal pleiotropy,^15^ thus hampering the establishment of causal inference. Overall, it is difficult to elucidate the underlying heterogeneous biological mechanisms of causal effects using all significant GWAS single nucleotide polymorphisms (SNPs) as IVs in MR analysis. We hypothesised that incorporation of single-cell expression data into a genetic risk variant colocalization and MR analysis will help determine adipose tissue- and cell-type level factors of abdominal obesity related to MASLD by selecting functionally important cell-type-aware WHRadjBMI GWAS eQTL IV SNPs that statistically contribute to the MR signal the same amount as regular GWAS SNPs, while also gaining functional insight into MASLD over non-cell-type oriented MR. To this end, we integrated adipose and liver single nucleus and bulk RNA-sequencing data with the UK Biobank data using colocalization and multiple MR methods, followed by functional validation experiments, which discovers 17 adipose cell-type-specific abdominal obesity genes contributing to MASLD.

## Methods

### Study cohorts

#### Kuopio Obesity Surgery Study (KOBS) cohort used for bulk and single nucleus RNA-sequencing

Finnish individuals with obesity undergoing bariatric surgery (*n* = 509) were recruited at the University of Eastern Finland and Kuopio University Hospital, Kuopio, Finland for the longitudinal Kuopio Obesity Surgery Study (KOBS) that included a 1-year follow-up, as previously described.^16^, ^17^, ^18^ The study was approved by the Ethics Committee of the Northern Savo Hospital District, in accordance with the Helsinki Declaration, and all participants provided written informed consent. Inclusion criteria of the study were a pre-surgery BMI of ≥40 kg/m^2^ or 35 kg/m^2^ with a significant comorbidity. In this study, we analysed existing subcutaneous adipose bulk RNA-seq data (*n* = 262)^18^ and liver bulk RNA seq data (*n* = 267)^17^ from the KOBS participants. In addition, subcutaneous adipose tissue biopsies from 8 KOBS participants were used for single nucleus RNA-sequencing (snRNA-seq).

#### The Finnish Twin and CRYO studies used for subcutaneous adipose snRNA-seq

Finnish monozygotic twins were recruited for the Finnish Twin study, a population-based longitudinal study in Finland, as previously described.^19^ For the CRYO study, a case–control dietary intervention study, individuals were recruited for a 12-month weight loss trial, as previously described.^18^^,^^19^ Existing subcutaneous adipose snRNA-seq data^19^ from 13 participants in the Finnish Twin study and 8 participants in the CRYO study were analysed in this study. The Finnish Twin and CRYO studies were approved by the local ethics committee, and all participants provided written informed consent.

#### Liver snRNA-seq cohort used for liver cell-type marker gene identification

Three female patients undergoing surgical resection for hepatocellular carcinoma (HCC) treatment at the Dumont-UCLA Liver Cancer Center were identified as MASLD-related HCC cases.^20^ All 3 patients were women with a mean BMI of 25.3 ± 2.9 kg/m^2^ with components of the metabolic syndrome including hypertension, dyslipidaemia, and insulin resistance. Features of nonalcoholic steatohepatitis (NASH) were observed on liver histopathology, and none had cirrhosis based on the METAVIR fibrosis score.^21^ Liver tissues were collected from tumour and adjacent non-tumour and were characterised by a pathologist. Existing liver snRNA-seq^20^ from the non-tumour tissues were used in our study. The study was approved by the UCLA IRB, and all participants provided written informed consent.

#### METabolic Syndrome In Men (METSIM) cohort used for linkage disequilibrium (LD) reference

Finnish males (*n* = 10,197) were originally recruited to the METabolic Syndrome In Men (METSIM) study at the University of Eastern Finland and Kuopio University Hospital, Kuopio, Finland, as previously described.^22^ The study was approved by the local ethics committee and all participants provided written informed consent. We used existing genotype data generated using an Illumina HumanOmniExpress BeadChip from 6686 unrelated METSIM participants in this study.^22^

#### UK Biobank cohort

The UK Biobank (UKB) consists of data from 502,617 individuals, collected from 22 different centres.^23^ As described previously, genotypes were obtained using either the Affymetrix or Applied Biosystems UK Biobank Axiom technology and imputed with the Haplotype Reference Consortium and the merged UK10K and 1000 Genomes phase 3 reference panel.^23^ Imaging data, including abdominal magnetic resonance imaging (MRI), was later collected for a subset of the cohort in follow-up assessments.^24^ In this study, analyses were limited to the unrelated individuals of European ancestry with phenotype data available. Data from UKB were accessed under application 33934.

### Genotype quality control and imputation

We extracted DNA for genotyping from blood samples from the 8 individuals with adipose snRNA-seq data in the KOBS cohort. The DNAs from the 8 individuals were genotyped on Illumina OmniExpressExome-8 array. The DNAs of the other KOBS participants were genotyped on an Illumina HumanOmniExpress BeadChip, as previously described.^19^

For all KOBS and METSIM genotype data, we filtered out unmapped and strand ambiguous SNPs, monomorphic SNPs, and variants with missingness >5%, Hardy–Weinberg Equilibrium (HWE) *p*-value <10^−6^, and minor allele frequency (MAF) < 0.5%. We inferred biological sex using the ‘--sex-check’ function in PLINK 1.9^25^ and cross-checked with sex identification from the phenotype data. No individuals with mismatching inferred and reported sex were observed. High-quality genotypes were then uploaded to the Michigan Imputation Server^26^ for imputation using HRC reference panel version r1.1 2016.^27^ Variants that did not match with the reference panel were removed and array build in GRCh38 was liftover to GRCh37. Haplotype phasing was done using Eagle v2.4^28^ and imputation was performed using Minimac4^26^ in the Michigan Imputation Server.^26^ We filtered imputed genotypes by R^2^ > 0.3 and MAF>0.5% and subset for 6686 unrelated individuals in METSIM. All quality control steps were performed using PLINK 1.9.^25^

### Bulk RNA-sequencing of human subcutaneous adipose and liver tissues

The bulk RNA-seq on subcutaneous adipose and liver biopsies from the KOBS cohort was performed as described previously.^17^^,^^18^ Briefly, total RNA was isolated and sequencing libraries were constructed using TruSeq library prep kit for adipose RNA and Ribo-Zero library prep kit for liver RNA. Sequencing was done using Illumina HiSeq 4000 and HiSeq 2500 platforms for adipose and liver RNA, respectively.^17^^,^^18^ We examined the RNA-seq quality using FastQC and obtained RNA-seq metrics using the function CollectRNAseqMetrics of Picard Tools v2.25.6.

### Nuclei isolation and snRNA-seq of human subcutaneous adipose tissue

We performed snRNA-seq experiments on 8 individuals from the KOBS cohort, as previously described with minor modifications.^19^ Briefly, we first pooled approximately 0.1 g frozen subcutaneous adipose biopsies obtained from 8 participants in the KOBS. We isolated nuclei from the pooled biopsy as described earlier^19^ with minor modifications. We measured nuclei concentration and determined the overall quality using Countess II by staining the nuclei with trypan blue and DAPI. We used the 10X Chromium platform with the Single Cell 3’ v3.1 protocol for library construction and sequenced the libraries on an Illumina NovaSeq SP at a sequencing depth of 600M.

Data from two additional snRNA-seq experiments on a total of 21 participants of the Finnish Twin study and CRYO study^19^ were also analysed. In the first snRNA-seq experiment, we isolated nuclei from frozen subcutaneous adipose biopsies (*n* = 6) obtained from six participants in the Finnish Twin study, constructed sequence libraries, and performed sequencing as previously described.^19^ In the second snRNA-seq experiment, we isolated nuclei from frozen subcutaneous adipose biopsies (*n* = 15) obtained from fifteen participants in the Finnish Twin (7 individuals) and CRYO (8 individuals) studies, constructed sequence libraries, and performed sequencing as previously described.^19^

### Nuclei isolation and snRNA-seq of human liver tissue

Nuclei from frozen liver biopsies (*n* = 3) were isolated as previously described.^20^ Briefly, the tissues were lysed and dounce homogenised then filtered to remove debris. Nuclei were centrifuged and washed for a second filter step, isolated nuclei were assessed for quantity and overall quality under BZ-X710 fluorescent microscope after staining them with Hoechst stain. We used the 10X Chromium platform with the Single Cell 3’ v2 protocol for library construction and sequenced the libraries on an Illumina NovaSeq S2 at a sequencing depth of 300–400 million reads per sample.^20^

### Processing of human subcutaneous adipose and liver tissue snRNA-seq data

We processed the snRNA-seq data of frozen human subcutaneous adipose biopsies obtained from 8 individuals in the KOBS cohort as previously described with minor modifications.^19^ Briefly, the sequence reads were aligned to the GRCh37 human genome reference using STARSolo in STAR v2.7.5a^29^ and GENCODE v19 annotation.^30^ We used the ‘--soloFeatures GeneFull’ option in STAR to account for full pre-mRNA transcripts. We removed droplets contaminated with background RNA using DIEM^31^ then further filtered droplets using Seurat v4.3.0.^32^ As nuclei from the 8 samples were pooled before sequencing, we demultiplexed clean droplets for each individual using demuxlet from popscle software tool.^33^ Demuxlet overlaps individual level genotype data with the RNA-seq data, and then evaluates the likelihood of overlaps from each droplet to determine originating individual of each droplet. We excluded doublet and ambiguous assigned droplets and used the best matching individual based on the maximum likelihood to identify originating individuals of each singlet droplet. The count data of the remaining droplets were log-normalised using the default scaling factor of 10,000 and top 2000 variable genes were calculated using the FindVariableFeatures. Normalised read counts were scaled to mean 0 and variance 1, and the first 30 PCs were calculated for clustering with standard Louvain clustering. We used clustering resolution of 0.8. To annotate each droplet with a cell type, we used SingleR v1.2.4^34^ with the same reference datasets used in the previous study.^19^ Marker genes for each cell type and cluster were identified by Wilcoxon rank-sum test using the function FindAllMarkers in Seurat^32^ with default parameters and only.pos = TRUE. We used the Bonferroni corrected *p*-values <0.05 to correct for multiple testing. The subcutaneous adipose snRNA-seq data from the CRYO and Twins cohorts (*n* = 21) and liver snRNA-seq data (*n* = 3) were processed as previously described.^19^^,^^20^

### Cell-type proportion estimation of bulk tissues

We estimated the cellular composition of subcutaneous adipose and liver bulk tissues using the reference-free, marker-based decomposition mode of the method Bisque.^35^ Briefly, the marker-based decomposition approach uses expression of top cell-type marker genes to perform a principal component analysis (PCA) to capture variation in cell-types across individuals. We aligned subcutaneous adipose (*n* = 262) and liver (*n* = 267) RNA-seq data from the KOBS cohort to the GRCh37 human genome reference using the 2-pass STAR v2.7.5a^29^ and GENCODE v19 annotation.^30^ Transcripts were quantified using featureCounts,^36^ and were Trimmed Mean of M-values (TMM) normalised and converted to counts per million (CPM) using edgeR v3.36.0.^37^ We then log2 transformed CPMs after adding a prior count of 1 and regressed out technical factors, including the percentage of intronic bases, median 3’ bias, and RNA integrity number (RIN) to get the final normalised expression data. Bisque was run with default parameters on the final normalised expression data and cell-type markers identified from the snRNA-seq of subcutaneous adipose and liver tissues. We decomposed cell-type proportions for 5 and 7 main cell-types in subcutaneous adipose and liver bulk tissues, respectively.

### *Cis*-eQTL analysis of human subcutaneous adipose and liver bulk RNA-seq data

To detect *cis*-eQTLs in subcutaneous adipose (*n* = 262) and liver tissues (*n* = 267), we obtained FPKMs of each gene and filtered for genes expressed (FPKM>0) in at least 90% of the samples. To exclude hidden confounders, we employed probabilistic estimation of expression residuals (PEER)^38^ analysis with inverse normal transformed FPKMs. We optimised for PEER factors on chromosome 20 by maximizing power to detect *cis*-eQTL variants while removing hidden confounders. The subcutaneous adipose and liver expression data were adjusted for 37 and 10 PEER factors, respectively. We inverse normal transformed PEER factor adjusted FPKMs and performed *cis*-eQTL analysis with the imputed genotype data (MAF>5%) using Matrix eQTL^39^ while adjusting for technical factors, including percentage of intronic reads, median 3’ bias, and RIN. We defined *cis* regions as ±1 Mb from the ends of genes and significant *cis*-eQTL with an FDR <0.05.

Since previous studies have shown that incorporating cell population distributions of each sample into the *cis*-eQTL analysis increases the power to detect eQTLs,^8^ we repeated the *cis*-eQTL analysis by including the estimated cell-type proportions as covariates. The inverse normal transformed expression data of subcutaneous adipose and liver tissues were adjusted for 41 and 10 PEER factors, and the above listed technical factors and the estimated proportions of 5 (adipocytes, adipose stem and progenitor cells (ASPCs), endothelial cells, macrophages, and T cells) and 7 (cholangiocytes, hepatic sinusoidal endothelial cells (HSECs), hepatocytes, lymphoid, myeloid, neural, and stellate cells) subcutaneous adipose and liver main cell-types were included as covariates in the *cis*-eQTL analyses.

To evaluate whether the quality of the adipose eQTL results improves after incorporating the estimated cell-type proportions as covariates, we performed Wilcoxon rank sum test on the effect sizes of the 2,231,645 common *cis*-eQTL SNP-gene pairs obtained with and without adjusting for cell-type proportions. As *cis*-eQTL SNPs in tight LD may drive the significant increase in the observed effect sizes, we conducted the Wilcoxon rank sum test (alternative = “greater”) by only including the lead *cis*-eQTL SNP for each of the 15,957 eGenes observed both before and after adjusting for cell-type proportions. We considered the effect sizes to be significantly different using *p*-value <0.05. The Wilcoxon rank sum test was done in R v4.1.0.

### Functional enrichment of adipocyte and ASPC marker and expressed genes

We used Webgestalt^40^ to test for enrichment of the Gene Ontology^41^ biological processes for the adipocyte and ASPC marker genes and compared them with the enrichments of the same number of randomly selected genes expressed in the respective cell-types. All expressed genes in the corresponding cell-type were used as the reference gene set in Webgestalt. We considered a gene to be expressed if it has at least three counts in at least three cells of a same cell-type in the KOBS adipose snRNA-seq data.^42^ We determined an enrichment to be significant using FDR <0.05.

### Colocalization of WHRadjBMI GWAS and adipose cell-type marker gene *cis*-eQTL variants

To assess the relationship between adipose cell-type marker genes and WHRadjBMI GWAS variants, we focused on the KOBS subcutaneous adipose tissue *cis*-eQTL SNPs regulating adipose cell-type marker genes that we identified in the KOBS adipose snRNA-seq data, while using the cell-type adjusted *cis*-eQTL results. WHRadjBMI GWAS variants (*p*-value < 5 × 10^−8^) from GIANT and UKB meta-analysis^4^ that associated with gene expression levels of adipose cell type marker genes in *cis* (FDR < 0.05) were tested for colocalization using coloc.abf function of the Bayesian method COLOC^43^ v5.1.0. For each adipose cell-type marker genes regulated by the WHRadjBMI GWAS *cis*-eQTL SNPs, we applied COLOC using all variants within ±1 Mb from the ends of the genes and are present in both the WHRadjBMI GWAS and KOBS adipose *cis*-eQTL datasets. Traditional colocalization analysis is done under the assumption of a single causal variant between the two traits.^43^ This limits the analysis to only the strongest signals (i.e. lead variants) that are colocalized, which is often an unrealistic assumption and results in missed true signals.^43^ To overcome this limitation, we fine-mapped genetic signals for the presence of multiple causal signals and tested for colocalization using coloc.susie function of COLOC.^43^ The imputed genotype data from METSIM and UKB were employed as the linkage disequilibrium (LD) reference for the KOBS adipose *cis*-eQTL and WHRadjBMI GWAS variants, respectively. We restricted the UKB genotype data to a subset of randomly selected 6686 unrelated Europeans to match the number of unrelated individuals included in the METSIM genotype data. We used default prior probabilities of 1 × 10^−4^ for priors that a variant is associated with either GWAS or eQTL individually. For prior probability that the two traits share causal variants, we set the prior probability to 5 × 10^−6^, as recommended.^44^ We evaluated sensitivity to prior probability of shared causal variants using ‘sensitivity’ function of COLOC. Variants were defined to be colocalized when the posterior probability H_4_ was >0.8 based on its wide use in previous studies^45^, ^46^, ^47^ as well as the original COLOC paper.^44^ We defined colocalized variants as adipose cell-type-aware WHRadjBMI GWAS *cis*-eQTL SNPs.

### Colocalization of MASLD GWAS and liver cell-type marker gene *cis*-eQTL variants

We assessed the relationship between liver cell-type marker genes and MASLD GWAS variants using KOBS liver *cis*-eQTL variants regulating liver cell-type marker genes that we identified in the liver snRNA-seq data. MASLD GWAS variants (*p* < 5 × 10^−8^) from Miao et al.^48^ that associated with gene expression level of liver cell type marker genes (FDR < 0.05) were tested for colocalization using COLOC^43^ v5.1.0, as described above.

### Tissue and cell-type-aware Mendelian randomization analysis

To assess putative causal relationships between WHRadjBMI and MASLD, we conducted a two-sample Mendelian randomization (MR) analyses using the adipose cell-type-aware WHRadjBMI GWAS *cis*-eQTL variants as IVs. In these MR analyses, we used WHRadjBMI^4^ as the exposure variable and the MASLD score (i.e. the previous NAFLD score) developed in Miao et al.^48^ as the outcome variable. We used independent (R^2^ = 0.001) adipose cell-type-aware WHRadjBMI GWAS *cis*-eQTL SNPs as IVs. To comply with assumptions of MR, we removed SNPs that are also MASLD^48^ GWAS variants (*p*-value < 5 × 10^−8^). Palindromic variants that are ambiguously aligned were also removed. We implemented cML-MA-BIC,^49^ inverse variance weighting (IVW),^50^ MR-PRESSO,^51^ and weighted median^52^ to investigate if there is a potential causal effect of WHRadjBMI on MASLD. To assess heterogeneity between estimates of individual genetic variants and horizontal pleiotropic outliers, we performed the Cochran's Q test^53^ and the global heterogeneity test of MR-PRESSO.^51^ Default parameters were used in MR-PRESSO for the global heterogeneity test.

To further investigate the MR results using additional phenotypes, we used WHR^4^ as the exposure, and three additional outcome phenotypes, i.e. the fatty liver index (FLI),^54^ the meta-analysis of the International Classification of Disease, 10th revision (ICD-10) based MASLD status^55^ and liver magnetic resonance imaging (MRI) proton density fat fraction (PDFF) in UKB. These additional MR analyses were performed employing MR-PRESSO^51^ using the adipose cell-type-aware WHRadjBMI GWAS *cis*-eQTL variants as IVs.

For the GWAS of these additional MR analyses, we used publicly available GWAS summary statistics^55^ from the ICD-10 based MASLD case–control scans from the deCODE genetics, FinnGen, and Intermountain cohorts to first perform a fixed-effect MASLD case–control GWAS meta-analysis using METAL.^56^ Studies were weighted by the standard errors of the effect sizes, and remaining default parameters were used, as described previously.^55^ We also conducted GWASs for binarized fatty liver index (FLI) (*n* = 275,467) and continuous liver MRI PDFF (*n* = 26,407) in UKB. We quantile normalised the PDFF outcome, and defined FLI cases (*n* = 143,323) as FLI ≥60, and controls (*n* = 132,144) as FLI <30.^54^ We used BOLT-LMM v2.3.6^57^ for the GWASs, where we included the top 20 genetic PCs, testing centre, genotyping array, sex for both outcomes, as well as age at baseline and age^2^ at baseline for FLI, and age at imaging, and age^2^ at imaging for PDFF.

To evaluate the performance of our new MR design that used the cell-type-aware WHRadjBMI GWAS *cis*-eQTL variants, we also conducted a two-sample MR analyses using all LD clumped (R^2^ = 0.001) WHRadjBMI GWAS variants (*p*-value <5 × 10^−8^)^4^ as IVs after removing palindromic variants and the SNPs that are also MASLD GWAS variants (*p*-value <5 × 10^−8^).^48^ Same MR, heterogeneity, and horizontal pleiotropy tests were implemented as described above.

We tested for reverse causality by using the MASLD score^48^ as the exposure variable and WHRadjBMI as the outcome variable. We used the liver cell-type-aware MASLD GWAS *cis*-eQTL SNP as an IV. We also further tested for reverse causality using all LD clumped (R^2^ = 0.001) MASLD GWAS variants (*p*-value <5 × 10^−8^)^48^ while removing palindromic variants and SNPs that are also WHRadjBMI GWAS variants (*p*-value <5 × 10^−8^).^4^ Same MR, heterogeneity, and horizontal pleiotropy tests were implemented as described above, except for the MR analysis using a single liver cell-type-aware MASLD GWAS *cis*-eQTL SNP as an IV where only IVW^50^ was performed. All MR analyses were done using the MendelianRandomization v0.5.1,^58^ TwoSampleMR v0.5.7,^59^ MR-PRESSO v1.0,^51^ MRcM v0.0.0.9000,^49^ and cause v1.2.0^60^ packages in R v4.1.0.

### Integration and clustering of the three subcutaneous adipose tissue snRNA-seq datasets

To analyse subcutaneous adipose snRNA-seq data from all three experiments (Finnish Twin Study, CRYO, and KOBS) (total *n* = 29), we performed data integration while controlling for experiment-specific effects. To integrate snRNA-seq data from a total of 29 individuals across the three cohorts, we first log-normalised counts within each experiment and then integrated the log-normalised counts using CCA.^32^ Integration was done using the FindIntegrationAnchors and IntegrateData functions in Seurat v4.3.0^32^ with the top 2000 features that are repeatedly variable across all 3 adipose snRNA-seq datasets. The integrated counts were scaled to mean 0 and variance 1, and the first 30 PCs were calculated for clustering. To identify the adipose cell-type clusters, we performed standard Louvain clustering with a resolution of 0.8 by the FindClusters function.

### Scoring of the average cell-type level expression of the 17 WHRadjBMI GWAS genes

To assess average preferential cell-type level expression of the 17 adipose cell-type-aware WHRadjBMI GWAS *cis*-eQTL target genes, used as IVs in our MR, we assigned module scores in both the KOBS (*n* = 8) and in the integrated (*n* = 29) subcutaneous adipose snRNA-seq data using the AddModuleScore function in Seurat v4.3.0.^32^ Module scores were calculated for each cell by subtracting the average expression level of the gene set by the aggregated expression of control features that are randomly selected from each bin. We calculated the module scores of 17 adipose cell-type-aware WHRadjBMI GWAS *cis*-eQTL target genes using log-normalised counts for both KOBS and integrated snRNA-seq data. To evaluate the difference in module scores between the adipocyte and non-adipocyte nuclei, we conducted the Wilcoxon rank sum test in R v4.1.0.

### Human primary preadipocyte (PAd) culture and differentiation

Cryopreserved human subcutaneous primary white PAd (Zen-Bio catalog # SP-F-2, lot L120116E) were seeded into PromoCell PAd growth medium (PromoCell C-27410) with 1% Gibco Penicillin-Streptomycin (ThermoFisher 15140122) and cultured according to PromoCell PAd culturing protocols. Cells were maintained in a monolayer culture at 37 °C and 5% CO2. Cells were propagated for the full experiment and not cultured beyond 5 passages.

The plating and differentiation of cells was staggered so that time points 1-day, 2-day and 4-day were collected at the same time, and time points 7-day, 14-day were collected at the same time. The 0-day (PAd) time point was collected separately. To induce adipogenesis, cells were plated at confluency into 12-well plates for both ATAC-seq and RNA-seq (4 technical replicates per time point and assay) and the following day, adipogenesis was initiated using PAd differentiation medium (PromoCell C-27436). The 1-day and 2-day time points were collected before any further media changes. For all other differentiation time points, 72 h after the PAd differentiation medium was added, it was replaced with adipocyte nutrition medium (PromoCell C-27438), following PromoCell PAd differentiation protocols.

### Processing of bulk expression data from the human primary preadipocyte differentiation experiment

We performed bulk RNA-seq on the 4 technical replicates of primary human preadipocytes, in which we induced a 14-day differentiation, and collected samples at the baseline (0-day), 1-day, 2-day, 4-day, 7-day, and 14-day time points. For the RNA collection, cells were washed with PBS once and then lysed with TriZOL (Invitrogen 15596026), and RNA was purified using Direct-Zol RNA Mini-Prep (Zymo Research R2061). Libraries were prepared using the Illumina TruSeq Stranded mRNA kit and sequenced on one lane of an Illumina NovaSeq S1 flowcell to produce an average of 42M (SD = 5M) reads per sample. We aligned reads to the GRCh38 human genome reference with GENCODE v39^30^ annotations using STAR v2.7.10a^29^ with the two-pass method and default options. We examined the RNA-seq quality using FastQC and obtained RNA-seq metrics using the function CollectRNAseqMetrics of Picard Tools v2.25. Non-uniquely mapped reads and reads mapped to the mitochondrial genome were removed. We then quantified fragments at the gene level with featureCounts v2.0.3.^36^

### Differential expression and clustering analyses of the adipocyte and ASPC marker genes during adipogenesis

To evaluate differences in the expression of the 10 adipocyte and ASPC marker genes that are adipose cell-type-aware WHRadjBMI GWAS *cis*-eQTL eGenes, during adipogenesis, we used the limma v3.50.3^61^ R package and the voom^62^ normalization method to first test for differential expression (DE) between the baseline and 7-day time points. No additional covariates were used since we used cell-line data, which are unlikely to have strong confounding effects from technical factors. P-values were corrected for multiple testing of 10 genes using Bonferroni.

We next tested the 10 genes for DE across all 6 time points over the 14 days. To perform the DE testing, we used the ImpulseDE2^63^ R package, which fits impulse models for each gene and then performs a log-likelihood ratio test on each impulse model against a constant model. ImpulseDE2 was run in case–control mode, and the original sample ID was included as a batch effect. We corrected P-values for multiple testing using FDR (FDR < 0.05).

To group the 10 genes based their temporal adipogenesis expression pattern across the 6 adipogenesis time points, we used DPGP^64^ with the default parameters in addition to the following parameters: --check_convergence --check_burnin_convergence --true_times --cluster_uncertainty_estimate.

### Processing of bulk assay for transposase accessible chromatin (ATAC)-seq data from the human primary preadipocyte differentiation experiment

We followed the Omni-ATAC protocol to generate the ATAC-seq data,^65^ as described previously.^66^ Libraries were sequenced on one lane of an Illumina NovaSeq S4 flowcell to produce an average of 126M (SD = 48M) reads per sample. Sequencing reads were processed according to the ENCODE ATAC-seq Data Standards and Processing Pipeline and as described previously.^66^ The quality control (QC) metrics are summarized in Supplemental Table S1. To get a list of consensus peaks, we first called peaks at each time point separately using MACS2^67^ v2.2.7.1 and peaks with a q-value < 0.01 were retained. Peaks in blacklisted regions and those with fewer than one peak (bin) count per million mapped reads (BPM) in more than 20% of samples were filtered out. The peak coverage filtering reduced the peak numbers per time point from an average of 160,250 (SD = 22,943) peaks to 109,959 (SD = 2408) peaks. The filtered peaks per time point were then merged using BEDTools^68^ to create the final consensus peak set of 135,190 peaks.

For the differential accessibility (DA) analysis across adipogenesis, we used the R package ImpulseDE2,^63^ using default case-only parameters except the ‘boolIdentifyTransients’ parameter was set to TRUE. We tested 21 of the consensus peaks containing our adipose cell-type-aware WHRadjBMI GWAS *cis*-eQTL variants targeting adipocyte and ASPC marker genes for DA across adipogenesis or variants in tight LD (R^2^ > 0.95) with them.

### Simpson-Golabi-Behmel Syndrome (SGBS) cell culture and differentiation

We repeated the preadipocyte differentiation experiment using the human preadipocyte cell strain Simpson-Golabi-Behmel Syndrome (SGBS; RRID: CVCL_GS28). SGBS cells have been frequently studied due to its high capacity for adipogenic differentiation.^69^ The cells were cultured in DMEM/F-12 Nut media (Lonza # BE12-719F) supplemented with 17 μM Pantothenate (Sigma, #P-5155), 33 μM Biotin (Sigma #B-4639), 10% fetal bovine serum (FBS), 1% penicillin-streptomycin until they are confluent. The cells were then induced to differentiate from day 0 by adding serum free 3FC differentiation medium (DMEM/F-12 supplemented with 17 μM Pantothenate, 33 μM Biotin, 1% penicillin-streptomycin, 0.1 μM cortisol (Sigma #H0888), 0.01 mg/mL transferrin (Sigma #T8158), 0.2 nmol triiodotyronin (Sigma #T6397), and 20 nM human insulin (Sigma #I9278)) with 2 μM rosiglitazone (Cayman Chemical # CAT 71740), 25 nM dexamethasone (Sigma # D-4902), 0.5 μM methylisobuthylxantine (Sigma #I5879) for 7 days. During post differentiation the cells were supplemented with 3FC medium which was replenished twice every week.

### Processing of the bulk expression data from the SGBS differentiation experiments

We first extracted the total RNA from the SGBS cells throughout the differentiation at the baseline (0-day), 2-day, and 7-day time points (3 technical replicates per time point) using miRNeasy micro kit (Qiagen). Then we prepared library samples using QuantSeq 3′ mRNA-seq library prep kit FWD (Lexogen) according to the manufacturer's instructions, amplified for 18 cycles, and sequenced with Illumina NextSeq 500 for 75 cycles. The raw QuantSeq RNA-seq reads from the adipogenesis experiment were first trimmed with cutadapt v3.5 using a polyA sequence concatenated to the standard Illumina adapter as the trimming target as recommended by the company of the QunatSeq 3′ mRNA-seq library prep kit FWD. Then we aligned the trimmed reads to the GRCh37 human genome reference using the 2-pass STAR v2.7.5a^29^ and GENCODE v19 annotation.^30^ We examined the RNA-seq quality using FastQC and obtained RNA-seq metrics using the function CollectRNAseqMetrics of Picard Tools v2.25.6.

### Replication of the adipocyte and ASPC marker gene differential expression and clustering during adipogenesis in the SGBS cells

The DE analysis for the adipocyte and ASPC marker genes between the baseline and 7-day time points as well as across all 3 time points during the 7-day SGBS cell adipogenesis were performed as described above for the human primary preadipocyte differentiation experiment. For the replication analysis in the SGBS cells, we tested for 8 out of the 10 adipocyte and ASPC marker genes that are adipose cell-type-aware WHRadjBMI GWAS *cis*-eQTL eGenes because two genes were not detected. We then grouped the 8 genes based their temporal adipogenesis expression pattern across the 3 time points using DPGP,^64^ as described above for the human primary preadipocyte differentiation experiment.

### Small interfering RNA (siRNA) knockdown of *PPP2R5A* and *SH3PXD2B* in the SGBS cells

The SGBS cells were seeded in a 6-well plate (1.6 × 10^6^ cells per well) and when they were 50–60% confluent, the cells were transfected with siRNA using lipofectamine RNAiMAX (Invitrogen) according to the manufacturer's instructions. The siRNA transfection mixtures were independently prepared and applied for each well. We used predesigned siRNAs from Thermo Fisher Scientific (scrambled (control) siRNA (60 nM & 150 nM), *PPP2R5A* (60 nM), and *SH3PXD2B* (150 nM)).

It has been observed that during differentiation the cells stop dividing due to the lack of serum in the differentiation media. It was observed that this phenomenon allows the cells to retain the siRNA transfection mix for up to 14 days, as previously shown.^70^ Baseline (0-day) time point samples were collected 48 h post incubation with transfection mix. We treated the rest of the samples with differentiation media (as described above) and collected the cells at 2-day (2D) and 7-day (7D) time points.

### Oil Red O (ORO) staining of the SGBS control and siRNA knockdown cells

The SGBS cells were seeded in a 12-well plate (8 × 10^4^ cells per well) with 3 technical replicates per condition and time point. At the indicated time points, we washed the cells with 1xDPBS twice and fixed with 4% paraformaldehyde for 30 min at room temperature (RT). Next, we removed the fixation solution and washed the cells twice with milliQH2O and once with 60% isopropanol for 5 min at RT. We then stained the cells with ORO (0.5 g/100 mL isopropanol) for 20 min at RT and washed them until no excess stain was visible. Finally, we counter stained the cells with hematoxylin for 1 min and washed off excess stain. We visualized the cells using EVOS Core XL microscope and quantified the ORO stain in preadipocytes and adipocytes by extracting the stain from the cells using 100% isopropanol. The ORO intensity from the wells were measured using a plate reader at 492 nm and the resulting values were then normalised with the cell number. The above process was repeated 2–4 times for each condition at each time point (i.e. 2-4 biological replicates with 3 technical replicates per condition and time point).

### Processing of the bulk expression data from the SGBS cell siRNA knockdown experiments

We first extracted the total RNA from the SGBS cells using miRNeasy micro kit (Qiagen). Then we prepared library samples using QuantSeq 3′ mRNA-seq library prep kit FWD (Lexogen) according to the manufacturer's instructions, amplified for 18 cycles, and sequenced with Illumina NextSeq 500 for 75 cycles. The RNA extraction, library construction, sequencing, and data processing of the SGBS cells from the scrambled (control) siRNA (60 nM and 150 nM), *PPP2R5A* siRNA knockdown, and *SH3PXD2B* siRNA knockdown conditions across the three differentiation time points, with 3–4 replicates per condition, were performed together with the non-transfected control SGBS cells as described above, for a total of 51 samples. One sample was removed due to the low number of sequence reads.

### Differential expression (DE) analysis of the SGBS cell siRNA knockdown expression data

We conducted a DE analysis between the *PPP2R5A* and *SH3PXD2B* knockdown samples to their respective scrambled controls for each of the three time points (baseline, 2D, and 7D). We quantified transcripts from uniquely mapped reads using featureCounts^36^ and removed lowly expressed genes with a minimum count of 10 or less summed across the samples within one group. Next, we TMM normalised the expression values and used the limma v3.50.3^61^ R package and the voom^62^ normalization method without correcting for technical covariates since the samples are from *in vitro* cell-line experiment with biological replicates. We used QuantSeq 3’ tag-based sequencing, and restricted the tested genes to adipocyte and ASPC cell-type marker genes and adipogenesis pathway genes from WikiPathways^71^ WP236. We considered a gene to be significantly DE using FDR<0.05.

### Quantification of secreted adiponectin protein in the *PPP2R5A* and *SH3PXD2B* knockdown cells

The concentration of adiponectin in the cell culture supernatant of SGBS cells was measured using human total adiponectin (DRP300) Quantikine ELISA kits (R&D systems). The cells were treated with siRNA as described above (scrambled (control) siRNA (150 nM), *PPP2R5A* (60 nM), and *SH3PXD2B* (150 nM)) and the supernatants were collected at the baseline (0D), 2-day (2D), and 7-day (7D) time points (3 technical replicates per condition and time point), which was then subjected to centrifugation to remove cell debris. The assay was performed following the guidelines provided by the manufacturer. The optical density was measured at 450 nm, and a wavelength correction was performed at 540 nm using CLARIOstar microplate reader. The concentration of the adiponectin in the cell culture supernatant was determined by interpolating it against a standard curve and then normalised based on the total protein content. Protein was extracted using radioimmunoprecipitation assay (RIPA) buffer and quantified using BCA protein assays (Thermo Fisher Scientific). The above process was repeated 3 times for each condition at each time point (i.e. 3 biological replicates with 3 technical replicates per condition and time point).

### Principal component analysis (PCA) of 17 abdominal obesity genes and differential expression of *DGAT2* by MASLD status

To search for a gene expression signature of the 17 abdominal obesity genes, we performed PCA on the expression data of the 17 genes using the subcutaneous adipose bulk RNA-seq data from the 262 individuals with obesity in the KOBS cohort. The expression values were first TMM normalised and then converted to CPM using edgeR v3.36.0.^37^ Next, we log2 transformed the normalised CPMs after adding a prior count of 1 and regressed out technical factors, including the percentage of intronic bases, median 3’ bias, and RIN as well as the adipose cell-type proportion estimates. The final normalised expression data for the 17 genes were then used to perform PCA. The difference in the gene signature of the 17 genes in the adipose tissue was evaluated by performing Wilcoxon rank sum test on the first principal component (PC1) between the individuals with healthy liver and MASLD.

We used the subcutaneous adipose and liver bulk RNA-seq data from the 262 individuals with obesity in the KOBS cohorts to compare the expression of *DGAT2* in each tissue between individuals with histology-based diagnosis of steatosis (*n* = 154), fibrosis (*n* = 115), or NASH (*n* = 81) *vs* the controls (*n* = 86 for all tests) using the Wilcoxon rank sum test. The adipose and liver bulk RNA-seq data were normalised and corrected for technical factors as described above.

### Ethics

All participants provided written informed consent to participate in this research. The KOBS (IRB approval study numbers 54/2005, 104/2008, and 27/2010) and METSIM (IRB approval study number 171/2004) studies were approved by the Ethics Committee of the Northern Savo Hospital District. The Finnish Twin study (IRB approval study number 270/13/03/01/2008) and CRYO study (IRB approval study number 255/13/03/01/2009) were approved by the Ethics Committee of the Hospital District of Helsinki and Uusimaa. The liver snRNA-seq study was approved by the UCLA (IRB approval study number 20–001319). The UK Biobank has approval from the North West Multi-centre Research Ethics Committee (MREC) as a Research Tissue Bank (RTB) approval (REC reference number 21/NW/0157). All research was performed in alignment with the principles of the Helsinki Declaration.

### Statistics

All statistical tests used, justification for their use, and multiple testing correction methods are described above. We performed *p*-value corrections for multiple testing using the Bonferroni procedure when the number of tested hypotheses was small (i.e., in tens) and otherwise using FDR since FDR is considered to be more liberal than the Bonferroni correction method and its power increases when the number of tests increases. In contrast, the Bonferroni procedure can be too conservative when a large number of not fully independent tests are performed (e.g. *cis*-eQTL analysis), potentially hiding true significant signals. Thus, we selected the Bonferroni and FDR multiple testing correction methods mainly based on the number of tests performed in each analysis while also considering how independent the particular measurements are.

### Role of funders

The funders did not have any role in the study design, data collection, data analyses, interpretation, or writing of this article.

## Results

### Study overview

To first search for biologically important, regulatory WHRadjBMI GWAS variants and their underlying regional adipose cell-type marker genes, we conducted the following steps using the Kuopio Obese Surgery Study (KOBS) bulk and single nucleus adipose expression data and previously published extensive WHRadjBMI GWAS data^4^ (Supplementary Figs. S1, S2): (1) gene expression profiling (bulk RNA-seq) of abdominal subcutaneous adipose biopsies from individuals with obesity, (2) analysis of adipose single nucleus RNA-seq (snRNA-seq) data to identify adipose cell-types and their marker genes, (3) integration of the snRNA and bulk RNA-seq data to decompose cell-type proportion estimates in the adipose bulk RNA-seq data, (4) adipose *cis*-eQTL analysis adjusting for cell-type composition, and (5) colocalization of WHRadjBMI GWAS variants with adipose *cis*-eQTL variants focusing on the SNPs regulating the expression of adipose cell-type marker genes as their target genes (i.e. eGenes). Then, we used these adipose cell-type-aware WHRadjBMI GWAS *cis*-eQTL SNPs as IVs to test for a putative causal effect of WHRadjBMI on MASLD by multiple MR methods and compared the results to the use of regular WHRadjBMI GWAS SNPs as IVs. Next, we examined the identified IV SNPs and their target cell-type marker genes during differentiation of human preadipocytes to adipocytes (i.e. adipogenesis) to elucidate their biological mechanisms. Two key eGenes were also knocked down during adipogenesis to test their effects on adipocyte lipidation, known adipogenesis regulators, and secretion of an adipokine, adiponectin (ADIPOQ). Finally, we tested the identified eGenes for an adipose gene signature in MASLD.

### Identification of WHRadjBMI GWAS SNPs that regulate expression of adipose cell-type marker genes

**Fig. 1:**
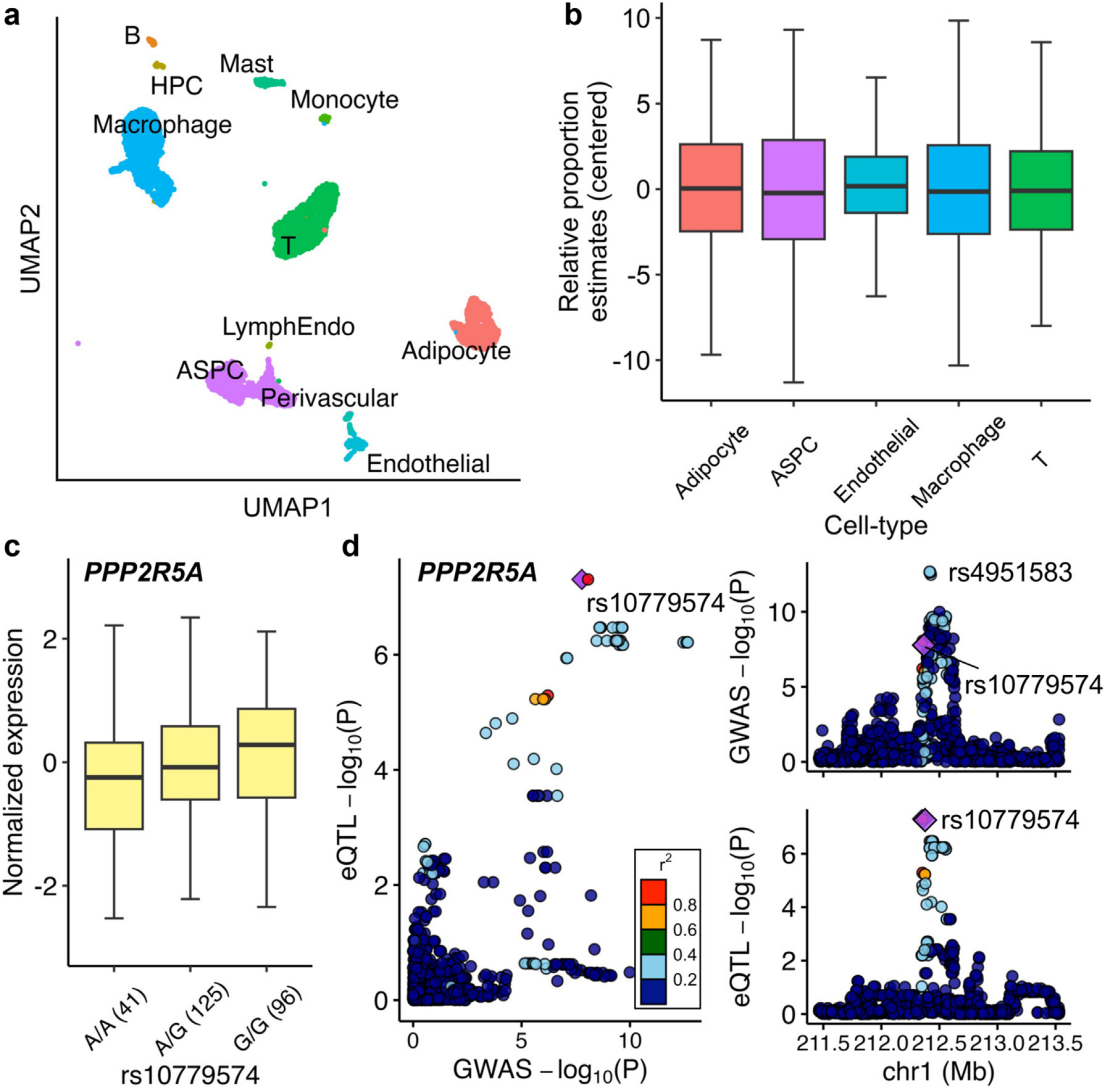
**Single-nucleus RNA-sequencing (snRNA-seq) and colocalization analyses identify adipose cell-type marker genes underlying abdominal obesity**. **a,** UMAP illustration of 11 cell-type clusters in snRNA-seq data of subcutaneous adipose tissue from 8 individuals with obesity in the KOBS cohort. ASPC indicates adipose stem and progenitor cells; HPC, hematopoietic stem cell; LymphEndo, lymphatic endothelial cells; and UMAP, Uniform Manifold Approximation and Projection. **b**, Main adipose cell-type proportions estimated in the 262 bulk RNA-seq data using Bisque.^35^ The box shows the 25th and 75th percentiles, the centre line shows the medians, and the whiskers extend to the 5th and 95th percentiles. **c**, *Cis*-eQTL effect for *PPP2R5A* in the subcutaneous adipose tissue. Boxplots show association between the genotypes of rs10779574 and the normalised expression of *PPP2R5A* from the adipose bulk RNA-seq data of the 262 individuals in the KOBS cohort. The box shows the 25th and 75th percentiles, the centre line shows the medians, and the whiskers extend to the 5th and 95th percentiles. **d**, Comparison of WHRadjBMI GWAS and adipose *cis*-eQTL SNPs (left panel) and regional overview of WHRadjBMI GWAS (top right panel) and adipose *cis*-eQTL loci (bottom right panel) demonstrates a significant colocalization of the WHRadjBMI GWAS and adipose *cis*-eQTL SNP rs10779574, targeting the gene *PPP2R5A*. The axes show the −log_10_ of *p*-values from the GIANT and UK Biobank WHRadjBMI GWAS meta-analysis^4^ and −log_10_ of *p*-values from the subcutaneous adipose *cis*-eQTL analysis in the KOBS cohort (*n* = 262). The colocalized *cis*-eQTL SNP, rs10779574, is represented by a purple diamond. Colors represent LD (r^2^) with colocalized *cis*-eQTL SNP. Chr indicates chromosome; eQTL, expression quantitative trait locus; GWAS, genome-wide association study; LD, linkage disequilibrium; Mb, mega base; P, *p*-value; SNP, single nucleotide polymorphism; and UMAP, Uniform Manifold Approximation and Projection.

We first performed a *cis*-eQTL analysis in 262 individuals with obesity from the KOBS cohort (see Methods) using their imputed genotype data (MAF > 5%) and subcutaneous adipose bulk RNA-seq data (Supplementary Table S2). Without LD pruning, we found 2.30 × 10^6^ SNP-gene pairs from 16,330 eGenes with an FDR < 0.05. To account for cell-type heterogeneity in the *cis*-eQTL analysis, we performed snRNA-seq on subcutaneous adipose biopsies (*n* = 8) to identify cell-type marker genes and determine cell-type proportions using the cell-type marker-based reference-free model of Bisque^35^ (Fig. 1a and Supplementary Table S3). As the accuracy of the marker-based decomposition mode of Bisque relies on cell-type specificity and co-expression of the top marker genes in each cell-type, we focused on estimating cell-type proportions of the 5 main adipose cell-types that include adipocytes, adipose stem and progenitor cells (ASPCs), endothelial cells, macrophages, and T cells (Fig. 1b and Supplementary Fig. S3). Including the cell-type proportions of the 5 main cell-types into the eQTL model replicated 97% of the identified significant SNP-gene pairs from the general eQTL model, likely because adjusting for PEER factors already captures a large part of the cell-type differences as hidden confounders. Nevertheless, 38,364 new adipose *cis*-eQTL SNPs and 326 new eGenes were discovered, and 368 genes were no longer regulated by significant *cis*-SNPs when compared to the *cis*-eQTL model not adjusted for the cell-type proportions. When comparing the results of the two *cis*-eQTL approaches (with and without correcting for cell-type proportions), we also observed that the effect sizes of the *cis*-eQTL SNP-gene pairs that were shared between the approaches significantly increased after adjusting for cell-type proportions (*p*-value < 2.23 × 10^−308^), thus demonstrating the improved quality of the eQTL results. Overall, we found 2,315,256 significant gene-SNP pairs in 16,372 eGenes when adjusting cell-type proportions. For the subsequent analyses, we used these data and prioritized the 105 eGenes that are marker genes of the 5 main adipose cell-types and have at least one SNP that is also a WHRadjBMI GWAS variant^4^ (*p*-value < 5 × 10^−8^).

We prioritized the eGenes that are cell-type marker genes as they are, by definition, uniquely expressed only in one adipose cell-type and may have important functions in the cell-types they are enriched in. To further demonstrate this, we conducted a functional enrichment analysis using Webgestalt^40^ with the marker genes of two of the key adipose tissue cell-types, adipocytes and ASPCs, and compared the number of significantly enriched biological processes (FDR < 0.05) obtained using these adipocyte and ASPC marker genes versus the same number of randomly selected expressed genes in the adipocyte and ASPC data. We observed 116 and 49 significant functional enrichments, centred around the key adipocyte and adipose tissue functions, with the adipocyte and ASPC marker genes whereas no significant functional enrichments were observed with the same numbers of random adipocyte and ASPC expressed genes, further confirming that the marker genes are more functional than random genes expressed in these cell-types (Supplementary Fig. S4).

### Identification of WHRadjBMI GWAS SNPs that colocalize with adipose cell-type marker gene *cis*-eQTL SNPs

We next performed colocalization between WHRadjBMI GWAS variants and adipose cell-type marker gene *cis*-eQTL variants using the Bayesian method COLOC.^43^ Among the 105 adipose cell-type marker gene *cis*-eQTL variants that are also the WHRadjBMI GWAS variants, 18 pairs of variants were colocalized with the posterior probability H_4_ > 0.8 (Table 1, Fig. 1c and d, and Supplementary Figs. S5–S7). We then tested for the possibility of multiple causal variants using COLOC-SuSiE,^43^ which uses Sum of Single Effects (SuSiE) regression framework to fine-map genetic signals and evaluate colocalization of multiple causal variants simultaneously. We identified 7 additional loci with evidence of colocalization (H_4_ > 0.8) with COLOC-SuSiE (Table 1 and Supplementary Figs. S5–S7). Then we checked for the independence among the 25 adipose cell-type marker gene *cis*-eQTL SNPs and removed four redundant SNPs in LD (R^2^ > 0.001) with the remaining SNPs, resulting in a final set of 21 adipose cell-type-aware GWAS *cis*-eQTL SNPs (Table 1, Fig. 1c and d, and Supplementary Figs. S5–S7).

**Table 1:** Colocalization analysis of WHRadjBMI GWAS SNPs and adipose cell-type marker gene *cis*-eQTL SNPs identifies 17 colocalized variants to be used as instrumental variables in the MR analyses (i.e. abdominal obesity → MASLD).

eGene^a^	Adipose cell-type^b^	WHRadjBMI GWAS^4^					Subcutaneous adipose *cis*-eQTL					COLOC^43^ (PP.H_4_)^d^	COLOC-SuSiE^43^ (PP.H_4_)^e^
		Variant	EA^f^	EAF	BETA	P	eSNP^c^	EA	EAF	BETA	FDR		
BCKDHB	Adipocyte	rs3812126	T	0.43	−0.01	4.81E-12	rs3805877∗	A	0.52	−0.39	2.81E-09		0.94
LHCGR	Adipocyte	rs17326656	T	0.23	0.02	5.77E-13	rs4519576	C	0.40	−0.73	3.28E-12	0.94	0.89
LONRF1	Adipocyte	rs4474021	T	0.31	0.01	2.44E-10	rs4474021∗∗	T	0.39	−0.69	7.33E-18		1.00
MYEOV	Adipocyte	rs6606672	A	0.65	−0.01	2.26E-13	rs7481709	C	0.51	−1.46	1.09E-39		0.98
PDE8B	Adipocyte	rs4704389	G	0.59	−0.01	1.74E-12	rs6864250	C	0.63	−0.57	1.26E-12	0.99	0.99
PPP2R5A	Adipocyte	rs4951583	T	0.59	−0.01	2.15E-13	rs10779574	G	0.61	0.42	4.94E-08	0.93	
TMEM132C	Adipocyte	rs7973997	T	0.36	0.01	2.20E-08	rs7973997	T	0.33	−0.42	3.50E-06	0.98	0.98
AHNAK	ASPC	rs2509963	C	0.74	0.02	9.36E-17	rs2509963	C	0.77	0.25	2.57E-06		1.00
EDEM2	ASPC	rs11696967	C	0.21	−0.01	6.78E-13	rs6120849	T	0.23	−0.43	2.96E-07		0.97
NF1	ASPC	rs3087591	G	0.32	−0.01	1.00E-09	rs7216033	G	0.35	0.16	6.41E-04	0.83	0.84
SH3PXD2B	ASPC	rs6859752	T	0.33	0.01	3.67E-10	rs6866204	A	0.28	0.62	3.71E-13	0.99	0.99
TSC22D1	ASPC	rs10507524	C	0.10	0.02	2.68E-09	rs9525915	G	0.61	−0.35	3.56E-04	0.90	
ATP2B4	Endothelial	rs2821231	C	0.54	−0.01	4.90E-10	rs2821231	C	0.53	0.41	1.65E-16	1.00	1.00
GPCPD1	Macrophage	rs805770	T	0.40	0.02	1.33E-33	rs805770	T	0.40	0.46	2.74E-09	1.00	1.00
PDCD6IP	Macrophage	rs10212473	G	0.24	0.01	5.70E-09	rs4678620	G	0.23	−0.29	1.04E-07	0.92	0.92
PDGFC	Macrophage	rs1425486	T	0.35	−0.01	2.91E-11	rs17228328∗	A	0.14	0.46	3.91E-05	0.92	0.89
SLC18B1	Macrophage	rs12190623	C	0.06	0.02	3.76E-07	rs12190623∗	C	0.12	−0.56	7.32E-06		0.93
SNX10	Macrophage	rs1534696	A	0.57	−0.02	3.15E-44	rs1534696	A	0.55	1.10	1.85E-53		1.00
ZZEF1	Macrophage	rs7225453	C	0.18	0.01	2.66E-10	rs8082227	T	0.20	0.31	8.50E-08	0.89	
LITAF	T	rs7102	C	0.35	0.01	1.38E-10	rs3784924	G	0.33	−1.32	4.87E-50	0.89	0.92
PCNX	T	rs2810073	C	0.69	0.01	1.91E-09	rs989501	A	0.69	0.49	5.68E-24	0.93	0.93

a Human subcutaneous adipose cell-type marker genes that are target genes of adipose *cis*-eQTL SNPs (eGene).

b Cell-types of the eGenes in human subcutaneous adipose tissue obtained using single-nucleus RNA-sequencing.

c *Cis*-eQTL eSNPs were used as instrumental variables in the MR analysis. All palindromic SNPs (marked with ∗) and SNPs associated with MASLD (marked with ∗∗) were removed from the MR analysis.

d Posterior probability (PP) of WHRadjBMI GWAS SNP and adipose *cis*-eQTL SNP colocalization (H_4_) measured using the coloc.abf function in COLOC.^43^ Only the PP.H_4_ > 0.8 are reported.

e Posterior probability (PP) of WHRadjBMI GWAS SNP and adipose *cis*-eQTL SNP colocalization (H_4_) measured using the coloc.susie function in COLOC.^43^ Only the PP.H_4_ > 0.8 are reported.

f EA indicates effect allele; EAF, effect allele frequency; eQTL, expression quantitative trait loci; FDR, false discovery rate; GWAS, genome-wide association study; P, *p*-value; PP, posterior probability; SNP, single nucleotide polymorphism; and WHRadjBMI, waist-to-hip ratio adjusted for body mass index.

The primary *cis*-eQTL SNPs or SNPs in tight LD (R^2^ > 0.8) for 12 genes (*ATP2B4*, *LHCGR*, *LITAF*, *NF1*, *PCNX*, *PDCD6IP*, *PDE8B*, *PPP2R5A*, *SH3PXD2B*, *TMEM132C*, *TSC22D1*, and *ZZEF1*) colocalized with the lead GWAS SNPs or SNPs in tight LD (R^2^ > 0.8) with the lead GWAS SNPs (Table 1, Fig. 1c and d, and Supplementary Figs. S5–S7). For *AHNAK*, *EDEM2*, *GPCPD1*, *MYEOV*, and *SNX10*, we observed colocalization after fine-mapping regional GWAS SNPs using the SuSiE method implemented in COLOC-SuSiE (Table 1 and Supplementary Figs. S5–S7).

As colocalization results can be sensitive to the selected prior probability, we also conducted a sensitivity analysis to evaluate robustness of our colocalization analysis results. The sensitivity analysis shows that our colocalized variants (posterior probability H_4_ > 0.8) are not sensitive to changes to prior probability that we used in the analysis (Supplementary Fig. S8).

### Tissue and cell-type-aware MR establishes a directional effect of abdominal obesity on MASLD

**Fig. 2:**
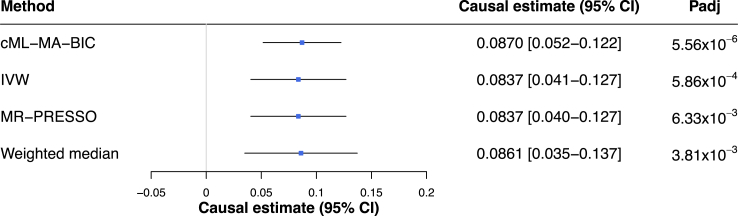
**Mendelian randomization (MR) demonstrates a putative causal effect of WHRadjBMI on MASLD using multiple MR methods**. Significant putative causal effects of WHRadjBMI on MASLD are demonstrated by multiple MR methods using adipose cell-type-aware WHRadjBMI GWAS *cis*-eQTL variants as instrumental variables (IVs). Forest plot of MR analyses shows causal estimates (betas) with 95% confidence intervals and *p*-values computed using cML-MA-BIC,^49^ inverse variance weighting,^50^ MR-PRESSO^51^ and weighted median.^52^ No outlier SNPs were detected by the MR-PRESSO outlier test in the MR analysis, and no significant evidence (*p*-value>0.05) for horizontal pleiotropy was observed by the MR-PRESSO global test. CI indicates confidence interval; IVW, inverse variance weighting; MASLD, metabolic dysfunction-associated steatotic liver disease; Padj, *p*-value adjusted for multiple testing using Bonferroni; and WHRadjBMI, waist-to-hip ratio adjusted for body mass index.

To search for possible causal relationships between abdominal obesity and MASLD, we performed a multilocus two-sample bi-directional MR analysis using WHRadjBMI as the surrogate for abdominal obesity and the MASLD score (i.e. the previous NAFLD score) that we developed in Miao et al.^48^ as the proxy for the MASLD risk in the UK Biobank. Our MASLD scoring resulted in 28,396 MASLD cases and 108,652 healthy individuals in the UK Biobank at a >90% confidence level.^48^ After removing one SNP directly associated with MASLD and three palindromic SNPs, we used the remaining 17 adipose cell-type-aware GWAS *cis*-eQTL SNPs from the colocalization analysis as the IVs in MR (F-statistic = 53.2). Using these adipose cell-type-aware WHRadjBMI GWAS *cis*-eQTL SNPs, we identified a significant putative causal effect of abdominal obesity on MASLD consistently using multiple MR methods that include cML-MA-BIC^49^ (theta = 0.087, *p*.adj = 5.56 × 10^−6^), inverse variance weighting (IVW)^50^ (beta = 0.084, *p*.adj = 5.86 × 10^−4^), MR-PRESSO^51^ (beta = 0.084, *p*.adj = 6.33 × 10^−3^), and weighted median^52^ (beta = 0.086, *p*.adj = 3.81 × 10^−3^) after correcting for multiple testing of 4 using Bonferroni (Fig. 2). Notably, no invalid IVs were detected among the 17 IVs when using the cML-MA-BIC method. In addition, we found no significant evidence of horizontal pleiotropy (*p*-value > 0.05) and no outlier IVs were detected in our data by MR-PRESSO. We further employed Cochran's Q test^53^ and observed no evidence for significant heterogeneity (Q = 26.079, *p*-value > 0.05). Taken together these results should not be confounded by horizontal pleiotropy or heterogeneity.

To further investigate the MR results using additional phenotypes, we employed waist-to-hip ratio (WHR) as another surrogate measurement for abdominal obesity, and the fatty liver index (FLI),^54^ meta-analysis of the International Classification of Disease, 10th revision (ICD-10) based MASLD status,^55^ and liver magnetic resonance imaging (MRI) PDFF as additional outcomes using the same 17 SNPs as IVs (F-statistic = 38.1 for WHR and F-statistic = 53.2 for WHRadjBMI). Employing first the WHR and MASLD score, we observed a significant putative causal effect in consistent direction of effect with MR-PRESSO (beta = 0.104, *p* = 6.25 × 10^−4^) (Supplementary Fig. S9). Next, we performed the MR analysis employing WHR and WHRadjBMI separately as the exposure trait and FLI as the outcome trait. We observed a significant putative causal effect with WHRadjBMI (beta = 0.187, *p* = 2.28 × 10^−3^) (Supplementary Fig. S9), and a similar putative causal effect with WHR (beta = 0.275, *p* = 3.86 × 10^−5^) after removing one SNP (rs7481709), detected as an outlier in this latter analysis by MR-PRESSO (Supplementary Fig. S9). We further conducted the MR analysis with WHR and WHRadjBMI separately as the exposure trait and the meta-analysis of the ICD-10-based MASLD status^55^ as the outcome trait, which had a smaller number (33.4%) of MASLD cases (*n* = 9491) when compared to the imputed MASLD score phenotype (*n* = 28,396 cases).^48^ We observed a trend in causal effect of WHR (beta = 1.417, *p* = 0.073) and significant causal effect of WHRadjBMI (beta = 1.323, *p* = 0.046) on the ICD-10 based MASLD status in the consistent direction of effect, both after removing one SNP (rs805770), detected as an outlier (Supplementary Fig. S9).

Lastly, we used WHR and WHRadjBMI separately as the exposure and liver PDFF as the outcome and observed a trend in causal effect of WHR (beta = 0.289, *p* = 0.0684) and WHRadjBMI (beta = 0.260, *p* = 0.0502) in consistent direction of effect on liver PDFF without any outliers by MR-PRESSO (Supplementary Fig. S9). Given that the number of individuals included in the liver PDFF GWAS (*n* = 33,011) is only 24% of the individuals included in the MASLD score GWAS (*n* = 137,048), thus decreasing the power for both the GWAS and subsequent MR analyses, these results with considerably smaller sample sizes further support the putative causal effect of abdominal obesity on MASLD.

To still further evaluate our MR results with WHRadjBMI and the MASLD score, we repeated the MR analysis using all LD clumped (R^2^ = 0.001), genome-wide significant WHRadjBMI GWAS variants (*p*-value<5 × 10^−8^) as IVs (*n* = 442, F-statistic = 63.4) after removing palindromic variants and SNPs that are also associated with MASLD (*p*-value<5 × 10^−8^). In line with our MR finding with the 17 IVs, we observed a significant (Bonferroni adjusted *p*-value < 0.05 corrected for multiple testing of 5) positive effect of abdominal obesity on MASLD using IVW (beta = 0.112, *p*.adj = 7.54 × 10^−174^), weighted median (beta = 0.109, *p*.adj = 7.86 × 10^−95^), MR-PRESSO after outlier correction (beta = 0.113, *p*.adj = 2.77 × 10^−102^), and cML-MA-BIC-DP (theta = 0.116, *p*.adj = 7.36 × 10^−184^) where data perturbation (DP) estimate model was used after rejecting the null hypothesis by the goodness-of-fit tests, as recommended^49^ (Supplementary Fig. S10); however, both MR-PRESSO global test and the Cochran's Q test revealed evidence of overall horizontal pleiotropy (*p*-value < 1 × 10^−4^) and heterogeneity (Q = 729.852, *p*-value = 1.34 × 10^−16^) after outlier correction, indicating that the MR modeling assumptions or IV assumptions have been violated.

We next tested for reverse causality using the MASLD score as the exposure trait and WHRadjBMI as the outcome trait. Adopting our tissue-of-origin, cell-type-aware IV selection MR approach, we identified liver cell-type-aware MASLD GWAS *cis*-eQTL SNPs to be used as IVs (Supplementary Fig. S11). We performed snRNA-seq on liver biopsies from 3 individuals^20^ to identify liver cell-types and their marker genes (Supplementary Fig. S11a and Supplementary Table S4), and utilised the marker gene-based decomposition mode of Bisque^35^ to estimate cell-type proportions of the 7 liver cell-types (Supplementary Fig. S11b). The liver *cis*-eQTL analysis was then performed adjusting for the 7 cell-type proportions (see Methods). After integrating liver snRNA-seq data with liver *cis*-eQTL results, we found that expression of 19 liver cell-type marker genes is regulated by at least one variant (FDR < 0.05) that is also a MASLD GWAS variant (*p*-value < 5 × 10^−8^). We also tested for colocalization between the MASLD GWAS and liver cell-type marker gene *cis*-eQTL variants in these 19 genes and observed significant colocalization in *RP11-152K4.2* with the posterior probability H_4_ > 0.8 (Supplementary Fig. S11c and d). Using liver cell-type-aware GWAS *cis*-eQTL SNP of *RP11-152K4.2* as an IV in IVW^50^ MR analysis (F-statistic = 63.2), we did not observe a significant directional effect of MASLD on WHRadjBMI (*p*-value = 0.250) (Supplementary Fig. 12a). Thus, no evidence for reverse causality was observed.

As only one liver cell-type-aware GWAS *cis*-eQTL SNP was identified for the reverse MR analysis, we further tested for the directional effect of MASLD on WHRadjBMI by using all LD clumped (R^2^ = 0.001) MASLD GWAS risk variants (*p*-value<5 × 10^−8^)^48^ as IVs. Even though we observed a possible causal effect of MASLD on WHRadjBMI using MR-PRESSO (beta = 0.239, *p*-value = 0.011) after removing three identified outliers (*n* = 16, F-statistic = 78.6), we also observed evidence for both significant horizontal pleiotropy and heterogeneity by the global test of MR-PRESSO (*p*-value < 1 × 10^−4^) and Cochran's Q test (Q = 33.53, *p*-value = 0.004) (Supplementary Fig. S12b), respectively, indicating that the MR modeling assumptions or IV assumptions have been violated.

### The identified WHRadjBMI genes show preferential expression in adipocytes

**Fig. 3:**
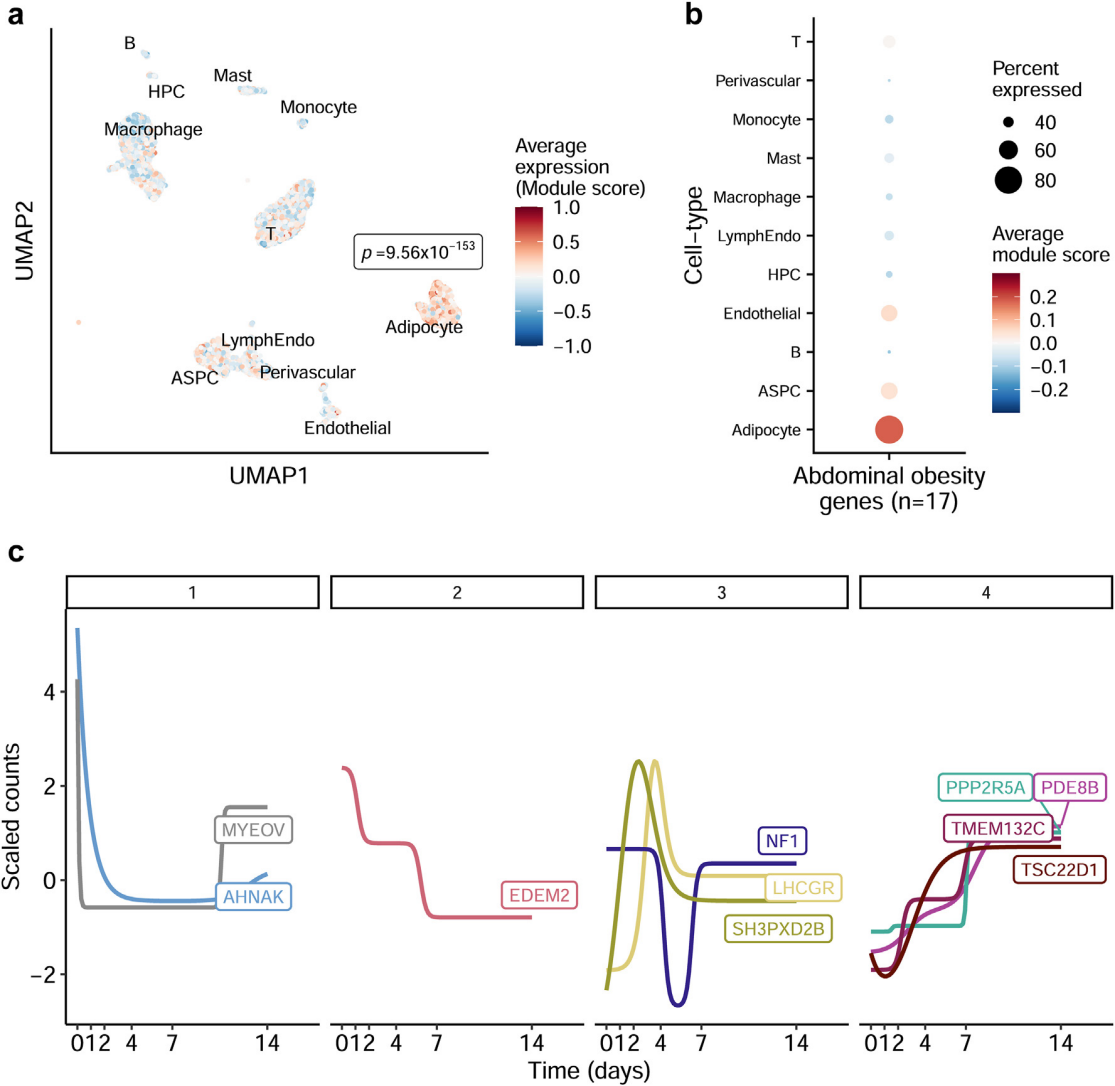
**Nuclei expression of the 17 adipose cell-type-aware abdominal obesity genes shows preferential average expression in adipocytes, and longitudinal expression of the 10 adipocyte and ASPC marker genes changes significantly across human adipogenesis**. **a**,**b**, The module scores of the 17 adipose cell-type-aware GWAS *cis*-eQTL target genes in the subcutaneous adipose snRNA-seq data (*n* = 8) show preferential average expression of the genes in the adipocyte cell-type. Module scores are calculated using the AddModuleScore function in Seurat^22^ as an average expression level of the 17 adipose cell-type-aware GWAS eQTL target genes while subtracting the aggregate expression level of the control feature sets for each nucleus. **a**, UMAP illustration of the adipose cell-type clusters where each dot represents a nucleus coloured by the module score of the 17 abdominal obesity genes. The *p*-value was computed using the Wilcoxon rank sum test to evaluate the difference in module scores of adipocyte *vs* non-adipocyte nuclei. **b**, Dot plot shows a higher average module score in the adipocytes. The size of each dot represents the percent of cells with a module score >0 in each cell-type and the colors represent an average module score for each cell-type. **c**, Human adipogenesis experiment shows longitudinal expression changes of the 10 adipocyte and ASPC marker genes during differentiation of human primary preadipocytes to adipocytes. Human primary preadipocytes were differentiated for 14 days and RNAs were collected at 6 time points for bulk RNA-sequencing. The genes were grouped into 4 distinct clusters based on the high probability of cluster assignment in their longitudinal expression trajectories during adipogenesis, detected using DPGP.^64^ Colors represent expression of the 10 adipocyte and ASPC marker genes from the 17 abdominal obesity genes quantified by bulk RNA-sequencing, and counts were normalised and scaled using ImpulseDE2.^63^ Gene-wise expression trajectory fits were obtained by implementing the impulse model, and the longitudinal differential expressions were evaluated using ImpulseDE2.^63^ ASPC indicates adipose stem and progenitor cells; HPC, hematopoietic stem cell; LymphEndo, lymphatic endothelial cells; and UMAP, Uniform Manifold Approximation and Projection.

To better understand the biological mechanisms underlying abdominal obesity by the 17 WHRadjBMI-associated variants used as IVs in our MASLD MR analysis, we further examined their *cis*-regulated adipose cell-type marker eGenes. Of the 17 genes, 5 are adipocyte marker genes (*LHCGR*, *MYEOV*, *PDE8B*, *PPP2R5A*, *TMEM132C*) while among the remaining 12 genes, there are 5 ASPC (*AHNAK*, *EDEM2*, *NF1*, *SH3PXD2B*, *TSC22D1*), 4 macrophage (*GPCPD1*, *PDCD6IP*, *SNX10*, *ZZEF1*), 2 T cell (*LITAF*, *PCNX*), and 1 endothelial cell (*ATP2B4*) marker genes, respectively (Table 1 and Supplementary Fig. S7). To assess the average cell-type level expression of these 17 WHRadjBMI GWAS *cis*-eQTL target genes in the subcutaneous adipose tissue snRNA-seq data from 8 individuals with obesity in the KOBS cohort, we used the module score analysis for each cell (see Methods) (Fig. 3a and b). We found that the expression module (i.e. average expression) of these genes is significantly higher (*p*-value = 9.6 × 10^−153^) in the adipocyte *vs* non-adipocyte nuclei using the Wilcoxon rank sum test (Fig. 3a and b). Integrating adipose snRNA-seq data from 21 additional individuals from the Finnish Twin^19^ and CRYO^18^^,^^19^ studies into our module score analysis further confirmed that the average expression of these 17 target genes is enriched (*p*-value<2.2 × 10^−308^) in adipocyte *vs* non-adipocyte nuclei. In contrast, the average expression of these 17 target genes is not enriched in any liver cell-types using the liver snRNA-seq data (Supplementary Fig. S11e and f). Taken together, our results indicate that the average expression of the target genes of the 17 WHRadjBMI GWAS *cis*-eQTL SNPs show significant preference for adipocytes, and thus they may induce changes in important adipocyte functions, ultimately impacting abdominal obesity.

### Adipocyte and ASPC marker gene expression dynamically changes during human adipogenesis

Based on our module score results that demonstrate the significant preferential average expression of the WHRadjBMI GWAS *cis*-eQTL target genes in adipocytes (Fig. 3a and b), we further investigated the expression changes of the genes during human adipocyte differentiation (i.e. adipogenesis). In this experiment, we differentiated human primary preadipocytes (*n* = 4 technical replicates), and measured expression of the 5 adipocyte (*LHCGR*, *MYEOV*, *PDE8B*, *PPP2R5A*, *TMEM132C*) and 5 ASPC (*AHNAK*, *EDEM2*, *NF1*, *SH3PXD2B*, *TSC22D1*) marker genes underlying the regional WHRadjBMI GWAS signals at 6 time points (Fig. 3c). We found that all 10 genes are significantly differentially expressed (DE) between preadipocytes at the baseline and 7 days after initiating the differentiation, and that they are also DE longitudinally across all 6 adipogenesis time points, using ImpulseDE2,^63^ and correcting for multiple testing of 10 genes using the Bonferroni adjusted *p*-value < 0.05 (Supplementary Table S5). We then used the DPGP tool^64^ to group the 10 genes into 4 distinct clusters based on their cluster assignment probability, similar longitudinal expression trajectories during adipogenesis (Fig. 3c and Supplementary Table S6), suggesting possible temporal co-expression or co-regulation of these genes.

We conducted an independent adipogenesis experiment for 7 days using the human preadipocyte cell strain derived from an infant with the Simpson-Golabi-Behmel Syndrome (SGBS)^69^ and observed an 88% replication rate with the DE between preadipocytes at the baseline and 7 days after initiating the differentiation and a 75% replication with the longitudinal DE genes detected in both experiments. We also observed a 67% replication rate with the temporal clustering genes among the replicated longitudinal DE genes in the human SGBS preadipocytes (Supplementary Tables S7 and S8). We consider these replication results promising given that one of the adipogenesis experiments was conducted using the human SGBS preadipocytes and the other one using human primary preadipocytes, and the fact that a slightly different RNA sequencing approach was applied in each experiment (see Methods). We limited the differentiation to just 7 days in the second adipogenesis experiment as we observed that the expression of key lipid droplet genes, *PLIN1* and *PLIN4*, are already induced during Day 7 of adipogenesis (Supplementary Table S9) consistently in our two independent adipogenesis experiments. These new replication results provide robust insight into how the longitudinal expression patterns and temporal clustering of these genes change during our two independent experiments of adipogenesis.

### Changes in adipocyte and ASPC marker gene expression during human adipogenesis reflect regional chromatin accessibility

**Fig. 4:**
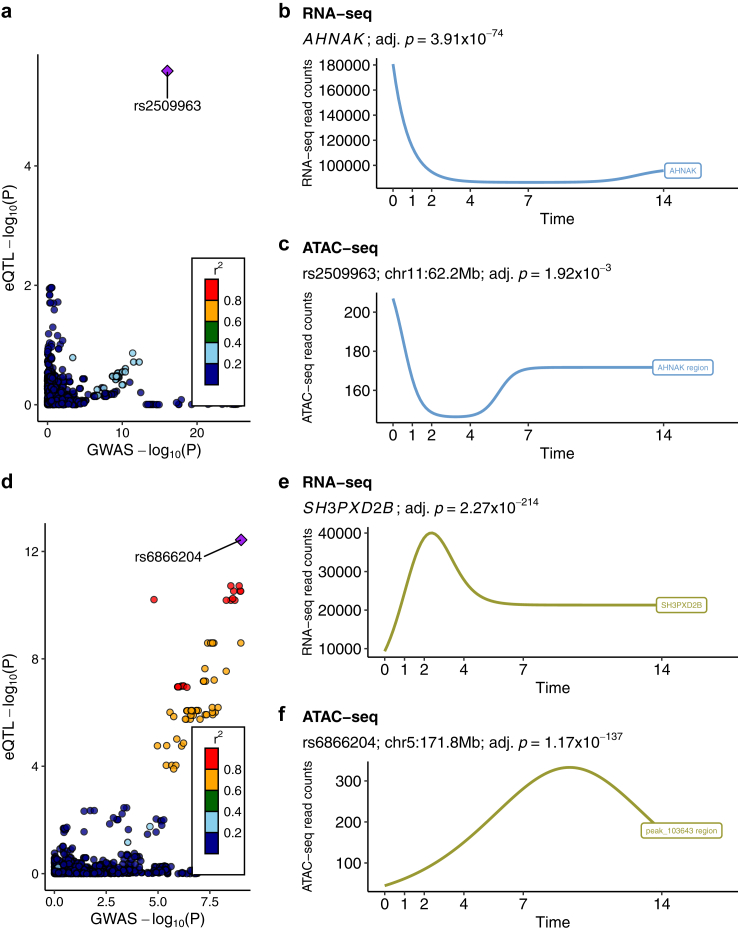
**Changes in adipose marker gene expression during human adipogenesis are reflected in regional chromatin accessibility at the *cis*-eQTL SNP sites**. **a**,**d**, Comparison of WHRadjBMI GWAS and adipose *cis*-eQTL SNPs demonstrates a significant colocalization of the WHRadjBMI GWAS and adipose *cis*-eQTL SNP rs2509963 targeting the gene *AHNAK* (**a**) and rs6866204 targeting the gene *SH3PXD2B* (**d**). The axes show the −log_10_ of *p*-values from the GIANT and UK Biobank WHRadjBMI GWAS meta-analysis^4^ and −log_10_ of *p*-values from the subcutaneous adipose *cis*-eQTL analysis in the KOBS cohort (*n* = 262). The colocalized *cis*-eQTL SNPs are represented by a purple diamond. Colours represent LD (r^2^) with colocalized *cis*-eQTL SNP. **b**,**e**, Bulk RNA-seq data of differentiating preadipocytes collected at 6 time points show significant differential expression (adj. *p*-value<0.05) of the adipose cell-type marker genes *AHNAK* (**b**) and *SH3PXD2B* (**e**) longitudinally during adipogenesis. **c**,**f**, Longitudinal bulk ATAC-seq data of differentiating preadipocytes collected at 6 time points show significant differentially accessible (adj. *p*-value < 0.05) chromatin regions that include the colocalized WHRadjBMI GWAS *cis*-eQTL SNPs targeting the adipose cell-type marker genes *AHNAK* (**c**) and *SH3PXD2B* (**f**). Gene-wise expression and chromatin accessibility trajectory fits were obtained by implementing the impulse model, and the longitudinal differential expressions were evaluated using ImpulseDE2.^63^ Significance of the longitudinal differential expressions and differential chromatin accessibility were measured using ImpulseDE2^63^ and corrected for multiple testing using Bonferroni corrected *p*-value < 0.05. Adj. *p* indicates adjusted *p*-value; chr, chromosome; eQTL, expression quantitative trait locus; GWAS, genome-wide association study; LD, linkage disequilibrium; Mb, mega base; P, *p*-value; and SNP, single nucleotide polymorphism.

We assessed the regulatory potential of the WHRadjBMI GWAS *cis*-eQTL SNPs targeting the 10 adipocyte and ASPC marker genes by examining chromatin accessibility of the IV SNP regions during adipogenesis. We performed ATAC-seq on differentiating human primary preadipocytes collected at the same 6 time points when the gene expression was measured. Focusing on ATAC-seq peak regions overlapping the IV SNPs, we identified 3 longitudinally differentially accessible (DA) chromatin regions that overlap rs2509963 *cis*-regulating *AHNAK*, rs7481709 *cis*-regulating *MYEOV*, and rs6866204 *cis*-regulating *SH3PXD2B*, throughout adipogenesis after correcting for multiple testing using the Bonferroni adjusted *p* < 0.05 (Fig. 4). Including SNPs in tight LD (R^2^ > 0.95) with the IV SNPs further revealed 15 additional DA chromatin regions overlapping one SNP in LD with *AHNAK cis*-eQTL SNP, 3 SNPs in LD with *EDEM2 cis*-eQTL SNP, 9 SNPs in LD with *NF1 cis*-eQTL SNP, one SNP in LD with *MYEOV cis*-eQTL SNP, and one SNP in LD with *PDE8B cis*-eQTL SNP.

We observed that the change in expression of the target eGenes of WHRadjBMI GWAS *cis*-eQTL SNPs during adipogenesis closely reflect the regional chromatin accessibility of the DA peaks that overlap the WHRadjBMI GWAS *cis*-eQTL SNPs (Fig. 4). For example, the expression of *AHNAK* and the chromatin accessibility of the region at the SNP rs2509963 site are both high in preadipocytes but they decrease once adipogenesis initiates (Fig. 4a–c), suggesting a potential enhancer role at the SNP site. Conversely, the expression of *SH3PXD2B* decreases in differentiating preadipocytes 2-days after initiating differentiation whereas the chromatin accessibility of the region at the SNP rs6866204 site continues to increase (Fig. 4d–f), suggesting a potential repressive role at the SNP site. Taken together, our results suggest that the target genes of the WHRadjBMI GWAS *cis*-eQTL SNPs are specific to adipose cell-types and their variant-specific functional changes may directly or indirectly impact key adipose tissue functions, such as adipogenesis.

### Knockdown of *PPP2R5A* and *SH3PXD2B* in human preadipocytes impairs adipogenesis

**Fig. 5:**
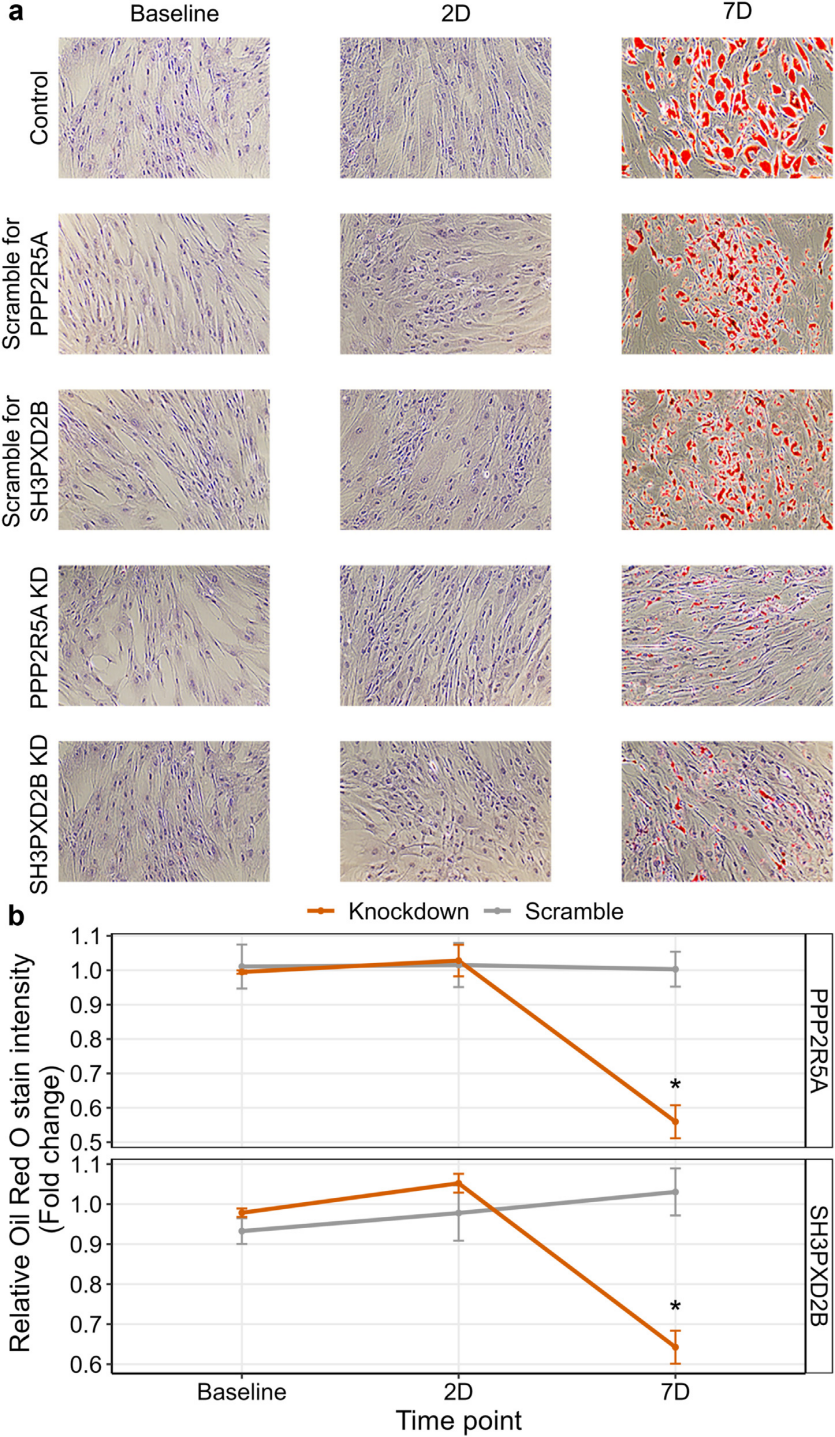
**Oil Red O (ORO) lipid staining reveals altered lipid accumulation with the knockdown of *PPP2R5A* and *SH3PXD2B* in human SGBS preadipocytes during adipogenesis**. siRNA-mediated knockdown (KD) of two abdominal obesity genes *PPP2R5A* and *SH3PXD2B* in the differentiating human SGBS preadipocytes shows disruption in lipid accumulation. The cells were stained with ORO for each condition at three time points, and the intensity of the ORO staining was quantified by measuring the absorbance of 492 nm wavelength light and normalizing to the cell number. **a**, Cell images of differentiating SGBS preadipocytes stained with ORO (red color) taken at 3 time points for each condition using the EVOS Core XL microscope at 20x zoom. Rows indicate experimental conditions as follows: non-transfected controls, scrambled siRNA control for *PPP2R5A* (60 nM), scrambled siRNA control for *SH3PXD2B* (150 nM), *PPP2R5A* knockdown (60 nM), and *SH3PXD2B* knockdown (150 nM). Columns indicate the number of days from initiating the differentiation of preadipocytes: 0-day (baseline), 2-days (2D), and 7-days (7D). **b**, Relative ORO intensity in each knockdown condition at each time point compared to their respective scrambled controls. The average ORO stain intensity from 2 to 4 biological replicates each with 3 technical replicates for each condition (colors) at each time point (dots) with ±standard deviation (error bars) was compared (fold change) to the average ORO stain intensity of the non-transfected controls at the respective time points. Significant differences in ORO intensities between the knockdown and scrambled control samples are shown (∗, *p*-value < 0.05) by the t-test.

As the preadipocyte differentiation experiment suggests potential functional role for the 10 adipocyte and ASPC cell-type marker genes during adipogenesis, we further tested for their impact on adipogenesis by separately knocking down the genes *PPP2R5A* (*PPP2R5A-KD*) and *SH3PXD2B* (*SH3PXD2B*-KD) in human SGBS preadipocytes during differentiation time course. These genes were selected based on previous human adipogenesis and mouse knockout experiments that showed evidence for their potential importance in adipogenesis.^72^^,^^73^ The knockdown was done via small interfering RNA (siRNA), and RNAs were collected at three time points for bulk RNA-seq (see Methods). To quantify preadipocyte differentiation to adipocytes in each of the knockdown and control experiment, we used Oil Red O (ORO) for staining of neutral triglycerides and lipids (Fig. 5 and Supplementary Fig. S13). When compared to the respective scrambled control group, we observed a significant decrease in the relative ORO stain intensity both in the *PPP2R5A*-KD (*p*-value = 0.0021) and *SH3PXD2B*-KD (*p*-value = 0.0028) groups at the 7D time point (Fig. 5b), indicating impaired lipidation of the developing adipocytes in the knockdown cells, hence less differentiation from preadipocytes to adipocytes. No significant difference in the relative ORO stain intensity was observed between the scrambled and non-scrambled controls, verifying that the observed differences in preadipocyte differentiation are not due to the technical effects from the siRNA transfection.

**Fig. 6:**
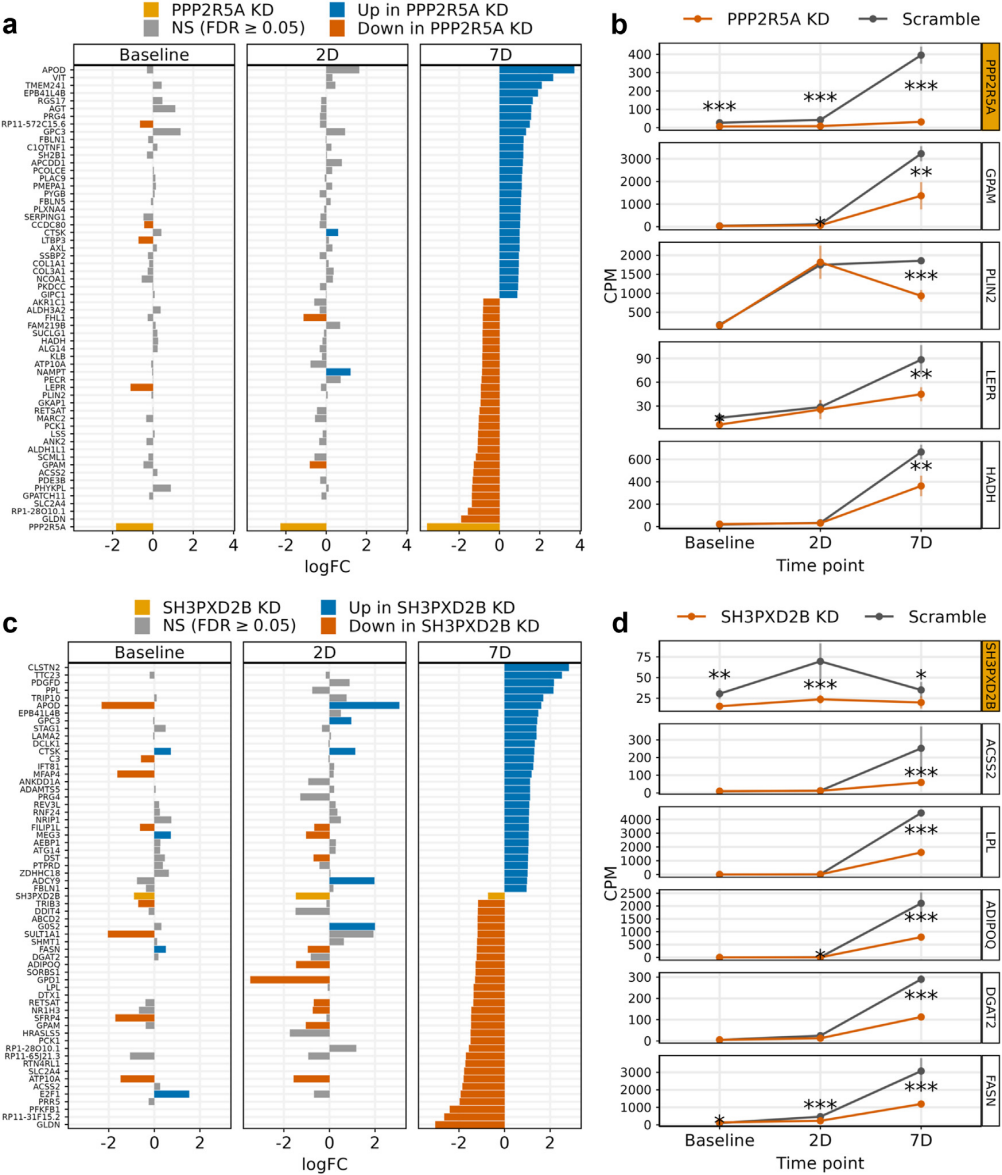
**Knockdown of *PPP2R5A* and *SH3PXD2B* in human SGBS preadipocytes identify altered expression of key adipogenesis genes**. **a**,**c**, Results of the differential expression (DE) analysis using the bulk RNA-sequencing data (see Methods) from the siRNA-mediated *PPP2R5A* (**a**) and *SH3PXD2B* (**c**) knockdown samples compared to the scrambled control samples. The top 30 significantly upregulated (blue) and 30 significantly downregulated (orange) genes by log fold-change in knockdowns compared to the respective scrambled controls (logFC) at the 7-day (7D) time point and the respective knockdown genes (gold) are shown for each time point sorted by the logFC at the 7D time point. All significant DE genes for each KD (passing multiple testing correction using FDR <0.05) are shown in the Supplementary Tables S10 and S11. **b**,**d**, Average expression, in counts per million (CPM), of the knockdown genes and selected key adipogenesis, fat storage regulator, and adipose tissue function genes are shown for each knockdown condition and scrambled controls at each time point. Dots represent average CPMs from 2 to 4 technical replicates with error bars indicating ± standard deviation. Time point is represented by the x-axis and the average expression (CPM) by the y-axis. The colours represent average expression in the knockdown *vs* respective scrambled controls. Significant differences in the average expression of each gene between the knockdown and scrambled controls samples at each time point are shown (∗, *p*-value<0.05; ∗∗, *p*-value<0.01; ∗∗∗, *p*-value < 0.001) by the t-test. FDR indicates false discovery rate; and NS, non-significant (FDR ≥ 0.05).

To examine the effect of *PPP2R5A* and *SH3PXD2B* knockdowns on adipogenesis, we performed DE analysis between each knockdown and their respective scrambled control at all three time points, limiting the tested genes to the adipocyte and ASPC marker genes and the adipogenesis pathway genes from WikiPathways (*n* = 1273 genes). First, we confirmed that both the *PPP2R5A* and *SH3PXD2B* genes are significantly downregulated in their respective knockdown conditions compared to their respective scrambled controls at all three time points (Fig. 6 and Supplementary Tables S10 and S11). Next, we found that the greatest number of DE genes are observed at the 7D time point for both knockdowns, which is in line with the largest difference observed in the ORO stain intensities at the 7D time point.

We observed 150 DE genes in the *PPP2R5A*-KD against the scrambled control at the 7D time point, of which 19 are adipogenesis genes from WikiPathways. The top 30 DE genes impacted by the knockdown of *PPP2R5A* are shown in Fig. 6a and all 150 significant DE genes for the *PPP2R5A*-KD (passing multiple testing correcting) in the Supplementary Table S10. These 150 DE genes include significantly decreased expression of *PLIN2* (log fold-change in knockdown *vs* scrambled control (logFC) = −0.925, FDR = 5.03 × 10^−4^) that encodes a protein involved in lipid droplet formation and *SCD* (logFC = −0.657, FDR = 8.47 × 10^−3^) that encodes an enzyme required for the biosynthesis of fatty acids (Fig. 6a and b and Supplementary Table S10). Our results suggest that *PPP2R5A* may be important for the regulation of fat modulation and energy homeostasis.

In the *SH3PXD2B*-KD at the 7D time point, we observed 330 DE genes when compared to the scrambled control, of which 35 are adipogenesis genes from WikiPathways including significantly decreased expression of *ADIPOQ* (logFC = −1.262, FDR = 1.26 × 10^−7^) that encodes an adipokine important for lipid metabolism, and *PPARG* (logFC = −0.686, FDR = 2.77 × 10^−5^), a master regulator of adipogenesis and lipid storage. The top 30 DE genes impacted by the knockdown of *SH3PXD2B* are shown in Fig. 6c and all 330 significant DE genes for the *SH3PXD2B* knockdown (passing multiple testing correcting) in the Supplementary Table S11. Among the 330 DE genes are 19 transcription factors (TFs), which includes significantly decreased expression of a fatty acid master TF *SREBF1* (logFC = −0.998, FDR = 5.93 × 10^−6^) and *TBX15* (logFC = −0.477, FDR = 0.041) (Supplementary Table S11). In addition, we found that the expression of 2 (*AHNAK* and *PPP2R5A*) out of 10 adipocyte and ASPC marker genes that are target genes of our IV SNPs for the MASLD MR analysis are also DE at the 7D with the knockdown of *SH3PXD2B*, which suggests that these genes may be co-regulated or share common molecular pathways (Supplementary Fig. S14).

As serum adiponectin levels are well known to be negatively correlated with multiple cardiometabolic risk traits,^74^ and we observed significantly decreased expression of *ADIPOQ* in the knockdown of *SH3PXD2B* at the 7D time point (Fig. 6d) and trend towards a decreased expression of *ADIPOQ* in the knockdown of *PPP2R5A* (*p*-value = 0.073), we performed an enzyme-linked immunosorbent assay (ELISA) experiment to quantify the secretion of the adiponectin protein by the *PPP2R5A*- and *SH3PXD2B*-KD SGBS cells during the differentiation at Days 0, 2, and 7. We observed a significant decrease in the secreted adiponectin levels for both the *PPP2R5A*-KD and *SH3PXD2B*-KD cells when compared to the control cells (*p*.adj = 7.33 × 10^−5^ and *p*.adj = 6.87 × 10^−3^, respectively) at Day 7 after correcting for multiple testing using Bonferroni (Supplementary Fig. S15).

**Fig. 7:**
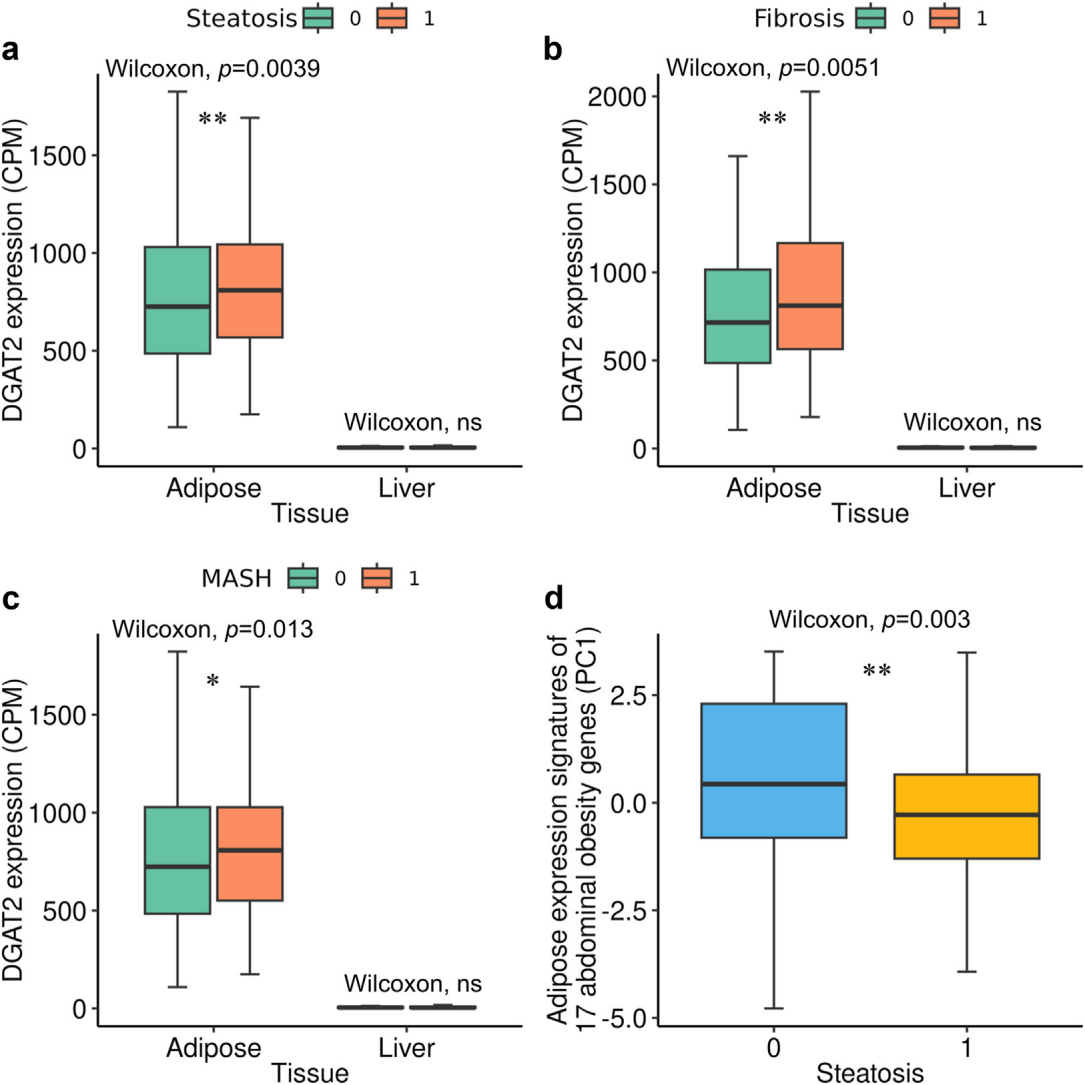
**Expression profiles of the 17 abdominal obesity genes and *DGAT2* in the adipose tissue demonstrate differences based on the liver phenotype status**. **a**,**b**,**c**, *DGAT2* expression quantified in the adipose tissue and liver from the bulk RNA-sequencing data of 262 individuals with obesity in the KOBS cohort shows higher expression in the adipose tissue when compared to the liver. Within the adipose tissue, *DGAT2* expression is significantly higher in the individuals with obesity and diagnosed with steatosis (*n* = 154) (**a**), fibrosis (*n* = 115) (**b**), or metabolic associated steatohepatitis (MASH) (*n* = 81) (**c**) using the liver histology-based assessment when compared to the controls with obesity and healthy livers (*n* = 86 for all comparisons). In contrast, the liver bulk RNA-sequencing data show no significant difference in the liver *DGAT2* expression based on the liver metabolic dysfunction-associated steatotic liver disease (MASLD) diagnosis groups. The x-axis represents the tissue where *DGAT2* is expressed and the y-axis represents *DGAT2* expression measured in Trimmed Mean of M-values (TMM) normalised counts per million (CPM). The colours represent the liver conditions (0 for the controls and 1 for steatosis, fibrosis, and MASH). Significant differences in the *DGAT2* expression between individuals with a liver disease *vs* controls were evaluated in each tissue by the Wilcoxon rank sum test using TMM normalised, log-transformed CPMs adjusted for technical factors and cell-type proportion estimates of their respective tissues (see Methods). **d**, The first principal component of the subcutaneous adipose expression of the 17 abdominal obesity genes is lower in the individuals with obesity and liver steatosis (*n* = 154) when compared to the controls with obesity and healthy livers (*n* = 86). The x-axis represents the hepatic liver diagnosis status (0 = controls and 1 = cases) and the y-axis represents the first principal component (PC1) of the principal component analysis (PCA) performed using the TMM normalised, log-transformed CPMs of the 17 abdominal obesity genes after adjusted for technical factors and adipose cell-type proportion estimates (see Methods). The box shows the 25th and 75th percentiles, the centre line shows the medians, and the whiskers extend to the 5th and 95th percentiles. The significance of the difference in PC1 of the adipose expression of the 17 abdominal obesity genes between the 2 groups was evaluated using the Wilcoxon rank sum test. **a-d**, The box shows the 25th and 75th percentiles, the centre line shows the medians, and the whiskers extend to the 5th and 95th percentiles, and the significant differences between the two groups are annotated (∗, *p*-value < 0.05; ∗∗, *p*-value<0.01; ∗∗∗, *p*-value < 0.001). NS indicates non-significant (*p*-value ≥ 0.05).

Notably, the *SH3PXD2B* knockdown significantly decreased expression of *DGAT2* (logFC = −1.227, *p*.adj = 5.85 × 10^−8^) (Fig. 6c and d), which encodes an enzyme that catalyzes the synthesis of triglycerides. Since *DGAT2* is currently tested as a new therapeutic target for treating MASLD/MASH,^75^^,^^76^ we further examined the expression of *DGAT2* in our group of 262 individuals with obesity from the KOBS cohort with bulk RNA-seq data from both their adipose and liver tissues and liver histology-based assessment of their clinical MASLD status. We first observed that *DGAT2* is highly expressed in the adipose tissue when compared to the liver (Fig. 7a–c), with significantly higher adipose expression (*p*-value = 4.23 × 10^−4^ by the Wilcoxon rank sum test) in the females (*n* = 182) than males (*n* = 80) with obesity. Randomly down sampling the number of females to 80 still showed a significant difference (*p*-value = 1.14 × 10^−3^) in the adipose tissue. Next, we compared the expression of *DGAT2* between individuals diagnosed with steatosis (*n* = 154), fibrosis (*n* = 115), or NASH (*n* = 81) *vs* the controls (*n* = 86 for all tests). We found significantly higher *DGAT2* expression in the adipose RNA-seq for all three liver phenotypes when compared to the controls (*p*-value = 0.0039, 0.0051, 0.013 for steatosis, fibrosis, and MASH, respectively) (Fig. 7a–c). No such differences were observed in their liver RNA-seq data (*p*-value > 0.05).

### Transcriptome profiles of the 17 abdominal obesity genes in the adipose tissue provide a gene signature for MASLD

We next examined whether the transcriptome profiles of the 17 abdominal obesity genes show a difference based on the histology-based liver disease status (healthy *vs* MASLD) of individuals with obesity given that these genes are regulated by the adipose cell-type-aware WHRadjBMI GWAS *cis*-eQTL SNPs used as IVs in our MASLD MR analysis and that we observed functional evidence for their impact on adipose tissue function via adipogenesis. We performed a principal component analysis (PCA) (see Methods) on the adipose bulk RNA-seq data of the 17 eGenes in KOBS (*n* = 262) and found that the first PC of these genes is significantly lower (*p*-value = 0.0031 by the Wilcoxon rank sum test) in the individuals with hepatic steatosis (MASLD) than controls with healthy livers (Fig. 7d). We further checked the 17 genes individually for DE in the adipose tissue by the liver status and found that *TMEM132C* was downregulated (*p*.adj = 8.09 × 10^−5^) in the individuals with histology based hepatic steatosis compared to the controls with healthy livers after correcting for multiple testing of 17 using Bonferroni. None of the other genes showed significant DE (*p*.adj ≥ 0.05).

In summary, we elucidate the biological basis of the statistical putative causal effect of abdominal obesity on MASLD, by integrating adipose snRNA-seq data with WHRadjBMI GWAS, adipose *cis*-eQTL, and colocalization data to select well-defined adipose tissue-of-origin, cell-type-aware GWAS *cis*-eQTL IVs for MR. Overall, we identified 17 abdominal obesity genes underlying the WHRadjBMI GWAS loci, and using their adipose cell-type-aware GWAS cis-eQTL SNPs, we further show a putative directional effect of abdominal obesity on MASLD with an adipose tissue and cell-type level biological origin.

## Discussion

Studying directional relationships, tissues and cell-types of origin, and biological mechanisms underlying associations between abdominal obesity and an increased risk of MASLD has been challenging due to the small effect sizes of obesity-associated GWAS variants, lack of large MASLD GWAS cohorts, and large pleiotropic overlap between the genetic loci of obesogenic metabolic traits.^77^^,^^78^ These factors hamper selection of well-defined IVs in MR by increasing horizontal pleiotropy and heterogeneity,^79^^,^^80^ which may cause a severe bias in MR.^79^^,^^80^ Significant heterogeneity in MR also points to pleiotropy and other possible violations of the necessary IV assumptions.^79^^,^^80^ Thus, the selection of genetic variants as IVs is very important in designing an MR analysis. For a polygenic analysis, two strategies have been suggested for selecting variants: (1) a biologically driven approach, in which genetic variants biologically linked to the exposure trait of interest are selected, and (2) a statistically driven approach, in which all genetic variants that are associated with the exposure trait of interest (e.g. GWAS variants) are selected.^81^ Selecting all exposure associated variants without a prior knowledge on their functions may lead to IVs that are pleiotropic,^81^ whereas a careful selection of well-defined IVs for an MR analysis can increase the effect sizes of the SNPs while decreasing the chance of potential horizontal pleiotropy and heterogeneity.^79^^,^^82^ Thus in this study, we used the biologically driven approach and prioritized colocalized WHRadjBMI-associated functional variants that regulate adipose gene expression at a cell-type level as IVs in our MR analysis, conducted using multiple MR methods, to establish a putative directional effect of WHRadjBMI on MASLD, and to define potential adipose tissue origin expression signatures for MASLD. Our biologically focused MR approach and functional experiments in human primary preadipocytes suggest that the 17 identified adipose cell-type marker genes underlying regional WHRadjBMI risk loci drive the adipose-origin development of MASLD, likely via obesity-induced adipose tissue dysfunction centred on adipogenesis, which ultimately promotes ectopic fat distribution into the liver.

In our search for WHRadjBMI-associated functional variants that regulate gene expression at a cell-type level, we colocalized WHRadjBMI GWAS SNPs with adipose *cis*-eQTL SNPs while prioritizing *cis*-eQTL SNPs, the target genes of which are adipose cell-type marker genes. These genes, by definition, are significantly enriched in one cell-type and they may have important functions at a cell-type level, which is supported by our significant functional enrichment results with the adipocyte and ASPC marker genes while no enrichments were obtained with the same numbers of random adipocyte and ASPC expressed genes (Supplementary Fig. S4). This design helped us detect cell-type level regulatory mechanisms underlying abdominal obesity. Although colocalization analyses between WHRadjBMI GWAS signals and subcutaneous adipose *cis*-eQTL signals have previously been conducted,^83^^,^^84^ these previous studies did not consider cellular heterogeneity of adipose tissue, which can confound the results and possibly mask cell-type-aware signals.^8^ We found that adjusting for cell-type composition in the *cis*-eQTL analysis increased the power to detect 38,364 new adipose *cis*-eQTL variants and 326 new eGenes, suggesting that some *cis*-eQTL and subsequent WHRadjBMI colocalized signals may have been missed in previous studies. We further show that adjusting for cell-type composition also significantly improved the effect sizes of the *cis*-eQTL variants, in line with previous studies showing improved quality of eQTL results after adjusting for cell-type proportions.^8^

We identified 17 WHRadjBMI-associated functional variants that are not directly associated with MASLD and used these variants as IVs to establish a causal effect of WHRadjBMI on MASLD with no evidence for significant horizontal pleiotropy or heterogeneity. When conducting the MASLD MR analysis, we used the MASLD score (MASLDS) that we recently developed as a non-invasive way to assess the MASLD phenotype in the UK Biobank (UKB).^48^ The MASLD scoring resulted in 28,396 MASLD cases and 108,652 healthy individuals at a >90% confidence level for our large MASLD GWAS in the UK Biobank, which identified 90 novel MASLD loci while also detecting the known MASLD loci.^48^ These large MASLD score GWAS data were used here for the abdominal obesity ↔ MASLD MR analysis.

The 17 colocalized WHRadjBMI GWAS *cis*-eQTL SNPs used as IVs in the MASLD MR analysis regulate 17 adipose cell-type marker genes underlying the regional WHRadjBMI GWAS loci, 5 of which are adipocyte marker genes (*LHCGR*, *MYEOV*, *PDE8B*, *PPP2R5A*, *TMEM132C*) and 5 are ASPC marker genes (*AHNAK*, *EDEM2*, *NF1*, *SH3PXD2B*, *TSC22D1*). Adipocytes are critically important for many adipose tissue key functions, including lipogenesis (i.e. storing fat) and lipolysis (i.e. burning fat).^85^ They also have major endocrine functions, which are critical for metabolic homeostasis.^85^ Dysfunction in adipogenesis, i.e. in differentiation of preadipocytes, which are included in ASPCs, to mature adipocytes can result in ectopic fat deposition to other tissues, such as the liver and around the heart, ultimately leading to the development of cardiometabolic diseases, including MASLD.^86^ We found that all 10 adipocyte and ASPC marker genes are DE between human preadipocytes measured at the baseline and differentiating adipocytes at the 7D time point, as well as longitudinally measured at 6 time points throughout adipogenesis, suggesting that these 10 genes may be directly or indirectly involved in adipogenesis or adipocyte functions. In addition, we found that the WHRadjBMI GWAS *cis*-eQTL SNPs, rs2509963 and rs6866204, regulating the expression of ASPC marker genes, *AHNAK* and *SH3PXD2B*, are located in the chromatin regions that are differentially accessible longitudinally throughout adipogenesis, suggesting that the expression of these genes reflects chromatin accessibility of their nearby *cis*-eQTL SNP regions.

In our knockdown experiment of *SH3PXD2B*, we observed 330 DE genes at the 7D time point of human adipogenesis. Notably, previous knockout experiments in mouse have shown decreased abdominal adipose tissue amount, decreased subcutaneous adipose tissue amount, abnormal adipose tissue development, and lipodystrophy with a knockout of *Sh3pxd2b*,^72^^,^^73^ and decreased total body fat amount and decreased susceptibility to diet-induced obesity with a knockout of *Ahnak*.^87^ The *Ahnak* gene directly interacts with *Smad1* on the *Pparγ2* promoter, increasing the expression of *Pparγ2*, an adipose tissue-specific isoform of *Pparγ*.^88^ *Pparγ* is a major regulator of adipogenesis promoting genes.^89^ Thus, *Ahnak* is an important upstream regulator of adipogenesis and is required to initiate differentiation of mouse preadipocytes.^89^ Similarly, *Sh3pxd2b* has been shown to play a role in the early stage of mouse adipogenesis.^88^ In a previous study on adipocyte differentiation of mouse mesenchymal stromal cells (MSCs), impaired expression of *Pparγ2* was observed by an immunoblot analysis for MSCs using the *Sh3pxd2b* encoded scaffold protein tyrosine kinase substrate with 4 SH3 domains (Tks4)knock-out mouse strain (Tks4^−/−^).^73^ Thus, both *AHNAK* and *SH3PXD2B* seem to impact adipogenesis via *PPARG2* in mouse although the exact role of *SH3PXD2B* and its underlying mechanism in human adipogenesis are not well known. In line with the mouse knockout of *Sh3pxd2b*, we first observed a disruption in differentiation of preadipocytes to adipocytes in the knockdown of *SH3PXD2B* in human primary preadipocytes, with a marked decrease in lipidation between the knockdown and control cells at D7 of adipogenesis. Importantly, we also show that knocking down *SH3PXD2B* significantly downregulates *AHNAK* already at the 2D time point. Furthermore, among the 19 TFs that were DE with the knockdown of *SH3PXD2B*, we observed downregulation of not only *PPARG*,^90^ but also of a fatty acid master TF, *SREBF1*^91^ and the TF *TBX15*, which we previously identified as a master *trans* regulator of abdominal obesity genes.^19^ In addition, the knockdown of *SH3PXD2B* downregulated expression of central genes involved in biosynthesis and regulation of fatty acids, such as *ADIPOQ*,^92^ *DGAT2*,^93^ *FASN*,^94^ *GPAM*,^95^ and *LPL*.^96^ The downregulation of the *ADIPOQ* expression is further supported by the significant decrease in the secretion of the adiponectin protein we observed. Adiponectin is an adipokine secreted by adipocytes, and it is well-known to have a role in attenuating metabolic diseases.^74^ Recent studies have suggested that adiponectin and its receptors in the liver may mediate ameliorative effects in obesity-induced MASLD, thus playing an important role in the crosstalk between the adipose tissue and liver.^97^ We found that the secretion of the adiponectin protein is decreased in the *SH3PXD2B* knockdown cells, which may just reflect the lower number of differentiating adipocytes. Nevertheless, this initial observation warrants additional functional studies to further elucidate the actual mechanism underlying this interesting link.

Interestingly, we observed a significant decrease in the expression of *DGAT2* when knocking down *SH3PXD2B* during adipogenesis. *DGAT2*, which was previously shown to be highly expressed in the adipose tissue and liver,^76^ encodes an enzyme that catalyzes the final step in triglyceride synthesis and has a regulatory role in very-low-density lipoprotein (VLDL) production.^98^ A systemic *DGAT2* inhibitor, together with a liver-targeted *ACC* inhibitor, is now under clinical trials for the treatment MASLD/MASH, which works by reducing hepatic lipogenesis.^75^^,^^76^ However, in the 262 individuals with obesity from the KOBS cohort, we observed a 166-fold higher expression of *DGAT2* in their adipose tissue than in their liver. We also observed significantly higher adipose expression among the individuals with obesity diagnosed with steatosis, fibrosis, or MASH by liver histology when compared to the controls with obesity and healthy livers. The function of *DGAT2* in triglyceride synthesis and VLDL production suggests that the observed increase in the adipose expression of *DGAT2* by the MASLD status might be important either through responsive or causal mechanisms for the accumulation of ectopic fat into the liver. Given that the *DGAT2* inhibitors under clinical trials target *DGAT2* systemwide,^76^ further studies evaluating the impact and consequence of inhibiting *DGAT2* in the obese and non-obese adipose tissue are warranted. Our study shows that the knockdown of another functionally relevant adipocyte marker gene, *PPP2R5A*, in human primary preadipocyte disrupts adipogenesis and decreases lipidation of differentiating adipocytes at D7. The knockdown experiment also resulted in significantly decreased expression of genes involved in biosynthesis and regulation of fatty acids, such as *FASN*,^94^ *GPAM*,^95^ and *LPL*,^96^ as well as in the expression of the body weight regulator, *LEPR*.^99^ Previous studies have shown *PPP2R5A* to be up-regulated during adipogenesis in human mesenchymal stem cells^100^ and associated with waist-adjusted BMI based on the EpiXcan analysis that utilised genotype, transcriptomics, and epigenetic data.^101^ The *PPP2R5A* gene encodes phosphatase 2A (PP2A) regulatory subunit B56α protein that binds to the LxxI/VxE motif of GSK3β and dephosphorylates the protein at Ser9, thereby increasing its activity.^102^ During adipogenesis, GSK3β activity mediates phosphorylation of β-catenin, which in turn blocks the Wnt activity and thereby drives adipocyte differentiation.^102^

Among the remaining 7 of the 17 identified WHRadjBMI genes, the *cis* regulatory variants of which were included as IVs in our WHRadjBMI → MASLD MR analyses, 4 are marker genes of macrophages (*GPCPD1*, *PDCD6IP*, *SNX10*, *ZZEF1*), 2 are marker genes of T cells (*LITAF*, *PCNX*), and 1 is a marker gene of endothelial cells (*ATP2B4*). Notably, the T cell marker gene *LITAF* encodes the lipopolysaccharide-induced TNF-alpha factor that binds to the promoter region of the TNF-alpha and mediates its expression.^103^ TNF-alpha is an adipokine known to negatively regulate adipogenesis and induce insulin resistance,^104^ which suggest that *LITAF* is an important upstream mediator of adipogenesis. Overall, previous mouse studies found evidence of obesity traits for 8 of the 17 genes that include *AHNAK*,^87^ *LHCGR*,^105^ *NF1*,^106^ *PDC6IP*,^107^ *PPP2R5A*,^102^ *SH3PXD2B*,^72^^,^^73^ *SNX10*,^108^ and *TSC22D1*.^109^ Additionally, *PDE8B* has previously been associated with monogenic syndromic obesity in humans.^110^ Taken together, our knockdown experiments in human primary preadipocytes and the previous mouse studies suggest that the WHRadjBMI GWAS genes we identified are functionally important in human adipose tissue particularly through adipogenesis and may determine body fat composition in animal models. Thus, our data support the conclusion that variant- and adipose cell-type-specific functional changes of these WHRadjBMI GWAS genes contribute to abdominal obesity.

Our study discovers an adipose gene signature of the 17 abdominal obesity genes for MASLD. This transcriptomic signature of the 17 genes in adipose tissue, captured by the first principal component from the expression PCA, was significantly lower in individuals with obesity and hepatic steatosis (MASLD) than controls with obesity and healthy livers. We compared the first PC of the adipose expression of the 17 genes between the two groups as we expected to observe a small difference or no difference between the two groups at an individual gene level given that the 17 genes were identified as the abdominal obesity genes in the adipose tissue with an indirect subsequent link to MASLD via impaired adipose tissue function. Together with our MASLD MR and gene knockdown analyses, this result suggests that this adipose gene signature marks the component in abdominal obesity that ultimately leads to hepatic fat accumulation via adipose tissue dysfunction.

In line with the previous studies that observed a putative causal effect of abdominal obesity, as measured by WHRadjBMI, on MASLD^13^^,^^78^ by using all significant (*p*-value<5 × 10^−8^) and independent WHRadjBMI GWAS variants from the GIANT or GIANT-UKB meta-analyses^4^ as IVs, we also repeated this possible causal path from abdominal obesity to MASLD; however, we observed significant heterogeneity by the Cochran's Q test in this overall MR analysis using all significant and independent WHRadjBMI GWAS variants from the GIANT-UKB meta-analysis^4^ as IVs. Our MR analysis using only the 17 colocalized SNPs, that regulate the abdominal obesity genes in adipocytes, was also able to generate the same MR signal between abdominal obesity and MASLD but without significant heterogeneity, thus showing a putatively more targeted directional effect of abdominal obesity on MASLD with an adipose tissue and cell-type level biological origin.

We initially tested for reverse causal effect between WHRadjBMI and MASLD using liver cell-type-aware MASLD GWAS *cis*-eQTL SNPs as IVs and found no significant directional effect of MASLD on WHRadjBMI. However, as only one colocalized MASLD GWAS signal and liver cell-type marker gene *cis*-eQTL signal was found, we further tested for the reverse causal effect using all MASLD GWAS variants^48^ as IVs. Although we found a significant possible causal effect of MASLD on WHRadjBMI, there was also significant evidence for horizontal pleiotropy and heterogeneity in this MR. A previous study,^13^ with only 1122 MASLD cases, used a small set of MASLD GWAS SNPs (7 variants in their study versus 19 in our study) as IVs and found a causal effect of MASLD on WHRadjBMI without evidence for horizontal pleiotropy and heterogeneity. However, based on their MR analyses and transgenic mice models, expressing human *PNPLA3* isoforms, that developed severe hepatosteatosis with an increase in abdominal obesity and decreased in overall body weight, Liu et al. proposed a phenotypic distinction between the genetically driven NAFLD (“lipodystrophic NAFLD”) that may be more likely to progress to “lean NAFLD” *vs* metabolically driven NAFLD (“metabolic NAFLD”) that is characterized by “obese NAFLD”.^13^ This distinction supports the recent international efforts, reflected by the change of nomenclature from NAFLD to metabolic-associated fatty liver disease (MAFLD)^111^ and even further to MASLD,^2^ to emphasize the importance of subphenotyping heterogenous groups of individuals with fatty liver and metabolic dysfunction. It is recognized that MASLD has complex causes that include interactions of genetic predisposition with environmental factors and metabolic dysfunction.^111^ This current study and our previous MASLD GWAS study^48^ support the importance of MASLD subphenotype grouping to better elucidate the genetic MASLD risk and its relationship to abdominal obesity and tissue and cell-type of origin at the individual level. Our study also clearly indicates that it would be important to focus on treating abdominal obesity to prevent obesity-related MASLD, which comprises ∼60–80% of MASLD cases, depending on the population.^112^

Our study has some limitations. We recognize that visceral adipose tissue expression and fat mass may also reflect the development of MASLD induced by abdominal obesity. However, prior studies (reviewed in ^113^) suggest that even though visceral adipose tissue is more prone to insulin resistance with unrestrained lipolysis and a pro-inflammatory profile than subcutaneous adipose tissue, the high expandability capacity of subcutaneous adipose tissue can provide a critical adaptive buffering mechanism against lipotoxicity, and thus MASLD. An additional practical benefit of using subcutaneous adipose tissue is that its biopsies are much less invasive than the visceral ones, which require a medically indicated surgical procedure. Thus, we used the subcutaneous adipose tissue as a fat depot proxy for abdominal obesity to search for a putative causal relationship between abdominal obesity and MASLD. Our results suggest that subcutaneous adipose tissue provides a readily available, adequate fat depot proxy for investigations of abdominal obesity by revealing an adipose-origin biological origin of MASLD without pleiotropy or heterogeneity.

The liver snRNA-seq cohort used in this study for the identification of liver cell-type marker genes comprised adjacent non-tumour liver biopsies from three females with MASLD-related hepatocellular carcinoma (HCC)^20^. All known main liver cell-types and their unique marker genes^114^ were observed in these data. However, we recognize that future larger studies generating human liver snRNA-seq data from both sexes and MASLD may provide additional liver cell-type marker genes that might have been missed in this study.

We also recognize the possible limiting factor that full differentiation of the SGBS cells takes 12–14 days.^69^^,^^115^ Our rationale to conduct the KD differentiation experiments using the SGBS cells for only 7 days was that we saw little to no change in gene expression of our two KD genes, *AHNAK* and *SH3PXD2B*, between Day 7 and Day 14 (Fig. 4b–e) in the human primary adipocytes that we did differentiate for 14 days. It also seemed that most expression changes with the 10 adipocyte and ASPC marker genes occurred during early adipogenesis in this same 14-day differentiation experiment. Furthermore, we observed disruption in lipidation of the developing adipocytes in the *SH3PXD2B*- and *PPP2R5A*-knockdown SGBS preadipocytes continuously through the 7 days of differentiation. Overall, our results from the 7-day differentiation experiment using SGBS cells suggest that the knockdown of the two genes already impairs the initiation and early stages of adipogenesis.

In summary, our study demonstrates that prioritizing adipose cell-type marker genes in colocalization analysis identifies regulatory abdominal obesity risk variants and their target genes with a cell-type-aware functional role. We also show that using these cell-type level regulatory eQTL WHRadjBMI GWAS variants as IVs in our MR analysis establishes a putative tissue- and cell-type-of-origin causal effect of abdominal obesity on MASLD, consistently by multiple MR methods. Overall, our approach gains functional insight into the adipose-origin MASLD, and the identified 17 cell-type-specific abdominal obesity genes provide potential therapeutic targets for treating abdominal obesity, thereby preventing obesity-driven MASLD.

## Contributors

S.H.T.L. and P.P. designed the study. S.H.T.L., Z.M., M.A., K.M.G., A.Kar, A.Koka, N.D., D.Z.P., and P.P. performed or supervised the computational and statistical analyses. U.T.A., T.O., and M.U.K. performed the human subcutaneous adipose tissue snRNA-seq from the KOBS biopsies. D.K., P.P., and J.P. collected the genotype data from the KOBS cohort. V.M. and J.P. collected the clinical and histological data from the KOBS cohort. M.A., S.H., P.P., and K.H.P. performed phenotyping, genotyping, and data collection from the Finnish Twin and CRYO cohorts. M.A., J.N.B., P.P., V.G.A., and J.R.P. collected clinical samples and performed liver tissue snRNA-seq. M.L. performed METSIM genotyping. K.M.G. and P.P. performed the human primary preadipocyte differentiation experiment. U.T.A., T.O., and M.U.K performed the siRNA-mediated knockdown experiments in SGBS preadipocytes. M.W. provided human SGBS preadipocytes for the preadipocyte differentiation experiment. S.H.T.L and P.P wrote the manuscript and all authors read, reviewed, and/or edited the manuscript. S.H.T.L, A.Kar, and P.P directly accessed and verified the underlying data reported in the manuscript.

## Data sharing statement

Summary-level data for the KOBS and liver snRNA-seq data are provided in the Supplementary Tables S1 and S2, respectively. The liver snRNA-seq data^20^ and the Finnish Twin and CRYO subcutaneous adipose snRNA-seq data^19^ are available from NIH GEO under accession numbers GSE189175 and GSE236708, respectively. The bulk RNA-seq data from the primary human preadipocyte differentiation experiment are available from NIH GEO under accession number GSE249195. The bulk ATAC-seq data from the primary human preadipocyte differentiation experiment are available from NIH GEO under accession number GSE269929. The KOBS subcutaneous adipose snRNA-seq data are available from NIH GEO under accession number GSE269926. Access to the existing KOBS bulk RNA-seq data are described in the original publications^18^^,^^116^ and KOBS adipose and liver *cis*-eQTL data are available at https://doi.org/10.25346/S6/SXMNDW. Data from the UK Biobank were used in this study under UK Biobank Application Number 33934. UK Biobank data are available for bona fide researchers through the application process (https://www.ukbiobank.ac.uk/learn-more-about-uk-biobank/contact-us).

## Declaration of interests

M.L. was funded by the Sigrid Jusélius Foundation during the last 36 months. The other authors declare no competing interests.
