# Supplementary material for: Single nucleus RNA-sequencing integrated into risk variant colocalization discovers 17 cell-type-specific abdominal obesity genes for metabolic dysfunction-associated steatotic liver disease
Source: eBioMedicine. 2024 Jul 10;106:105232. doi: 10.1016/j.ebiom.2024.105232 (PMC11663762; doi:10.1016/j.ebiom.2024.105232)
Supplement: Supplementary Figures and Tables [file mmc5.pdf]

Supplemental Information

Single nucleus RNA-sequencing integrated into risk variant colocalization discovers 17 cell-type-specific abdominal obesity genes for metabolic dysfunction-associated steatotic liver disease

Seung Hyuk T. Lee[1], Kristina M. Garske[1], Uma Thanigai Arasu[2], Asha Kar[1,3], Zong Miao[1,3], Marcus Alvarez[1], Amogha Koka[1], Nicholas Darci-Maher[1], Jihane N. Benhammou[4], David Z. Pan[1,3], Tiit Örd[2], Dorota Kaminska[5,6], Ville Männistö[7,8], Sini Heinonen[9], Martin Wabitsch[10], Markku Laakso[7], Vatche G. Agopian[11], Joseph R. Pisegna[12], Kirsi H. Pietiläinen[9,13], Jussi Pihlajamäki[5,14], Minna U. Kaikkonen[2], Päivi Pajukanta[1,3,15*]

[1]Department of Human Genetics, David Geffen School of Medicine at UCLA, Los Angeles, CA, USA
[2]A. I. Virtanen Institute for Molecular Sciences, University of Eastern Finland, Kuopio, Finland
[3]Bioinformatics Interdepartmental Program, UCLA, Los Angeles, CA, USA
[4]Vatche and Tamar Manoukian Division of Digestive Diseases and Gastroenterology, Hepatology and Parenteral Nutrition, David Geffen School of Medicine at UCLA and VA Greater Los Angeles HCS, Los Angeles, CA, USA
[5]Institute of Public Health and Clinical Nutrition, University of Eastern Finland, Kuopio, Finland
[6]Department of Medicine, Division of Cardiology, UCLA, Los Angeles, CA, USA
[7]Institute of Clinical Medicine, Internal Medicine, University of Eastern Finland, Kuopio, Finland
[8]Department of Internal Medicine, Kuopio University Hospital, Kuopio, Finland
[9]Obesity Research Unit, Research Program for Clinical and Molecular Metabolism, Faculty of Medicine, University of Helsinki, Helsinki, Finland
[10]Division of Pediatric Endocrinology and Diabetes, Department of Pediatrics and Adolescent Medicine, University of Ulm, Ulm, Germany
[11]Department of Surgery, David Geffen School of Medicine at UCLA, Los Angeles, CA, USA
[12]Department of Medicine and Human Genetics, Division of Gastroenterology, Hepatology and Parenteral Nutrition, David Geffen School of Medicine at UCLA and VA Greater Los Angeles HCS, Los Angeles, CA USA
[13]Healthy WeightHub, Endocrinology, Abdominal Center, Helsinki University Central Hospital and University of Helsinki, Helsinki, Finland
[14]Department of Medicine, Endocrinology and Clinical Nutrition, Kuopio University Hospital, Kuopio, Finland
[15]Institute for Precision Health, David Geffen School of Medicine at UCLA, Los Angeles, CA, USA
*Correspondence: ppajukanta@mednet.ucla.edu

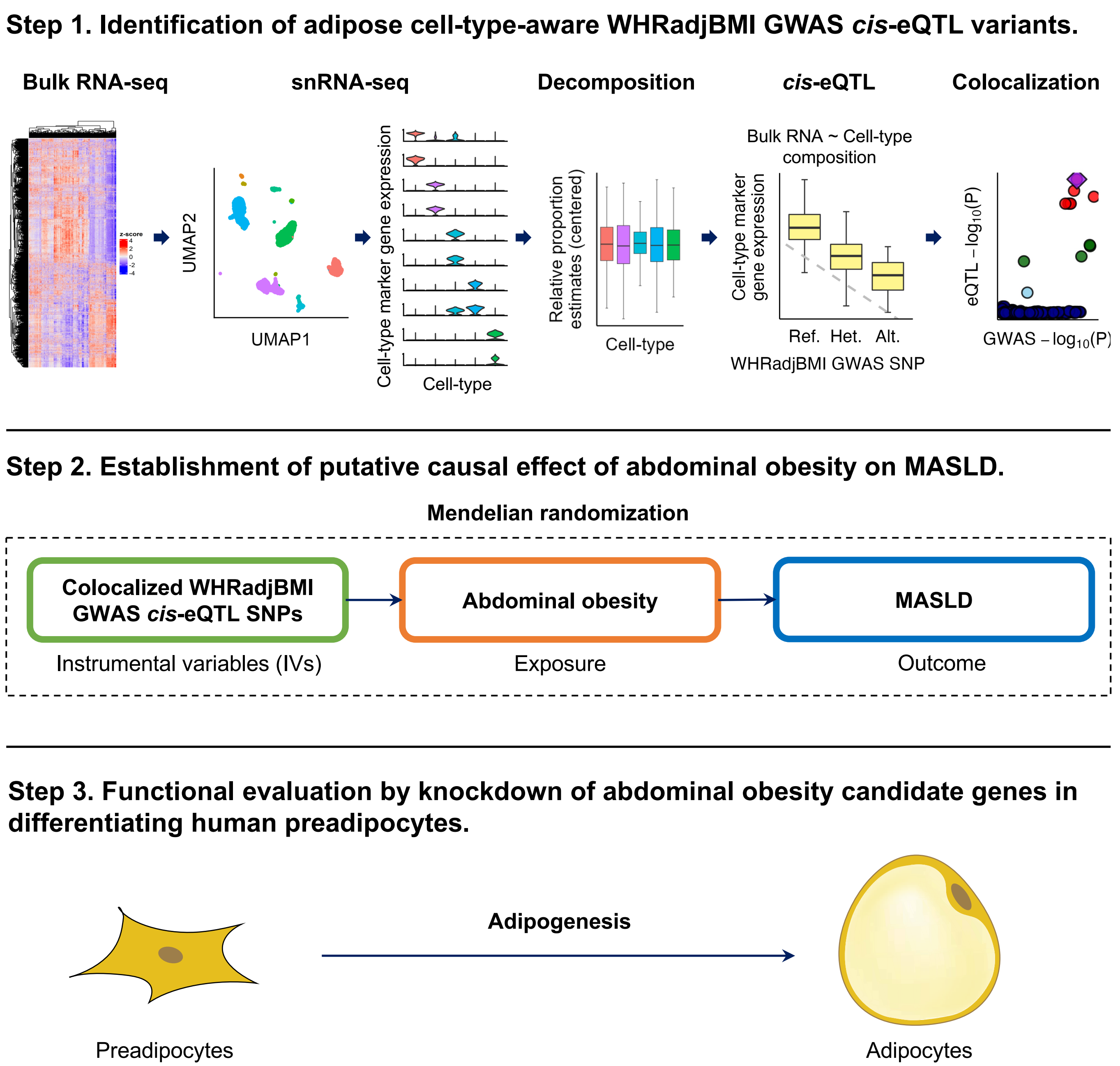

Supplementary Fig. 1. Schematic overview of the study design aiming to gain functional insight into MASLD over non-cell-type oriented MR. Step 1 consists of gene expression profiling (bulk and single nucleus RNA-sequencing) of abdominal subcutaneous adipose biopsies from obese individuals, estimation of adipose cell-type proportions, *cis*-eQTL analysis, and colocalization with waist-to-hip ratio adjusted for body mass index (WHRadjBMI)-associated variants to identify adipose cell-type-aware WHRadjBMI GWAS *cis*-eQTL variants. Step 2 includes Mendelian randomization analysis using the colocalized WHRadjBMI GWAS *cis*-eQTL variants from Step 1 as instrumental variables (IVs) to test for a putative causal effect of WHRadjBMI on MASLD. Finally, in Step 3, abdominal obesity candidate genes from previous steps are functionally evaluated by knockdown in differentiating human preadipocytes. *Cis*-eQTL indicates *cis*-expression trait locus; GWAS, genome-wide association study; MASLD, metabolic dysfunction-associated steatotic liver disease; P, *p*-value; SNP, single nucleotide polymorphism; snRNA-seq, single nucleus RNA-sequencing; and UMAP, Uniform Manifold Approximation and Projection.

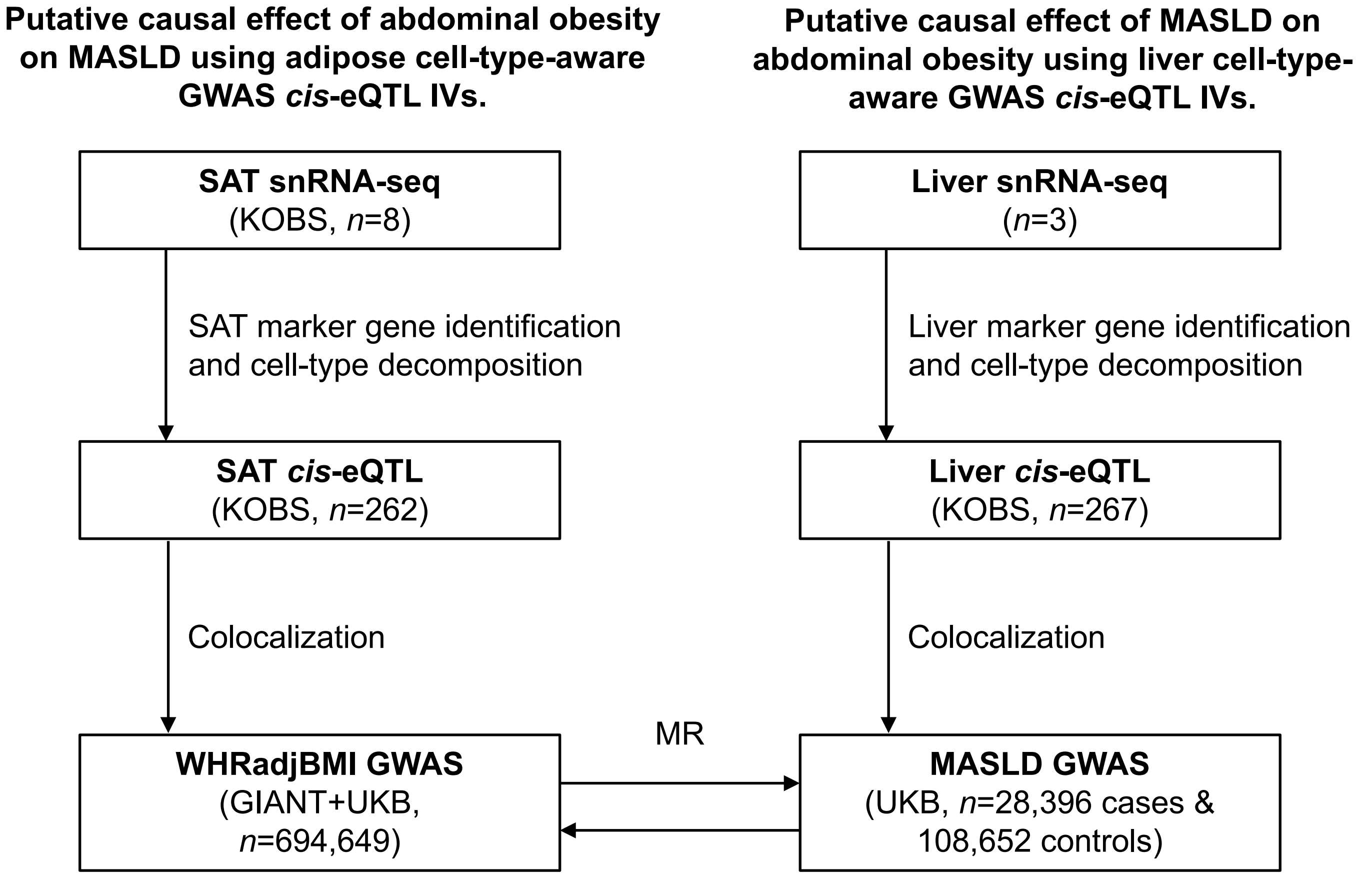

Supplementary Fig. 2. Flowchart of the samples included in the cell-type-aware Mendelian randomization (MR) analysis. The number of individuals included for the adipose and liver cell-type-aware MR analysis is shown for each cohort. Adipose and liver cell-type-aware GWAS *cis*-eQTL IV SNPs were identified by integrating SAT and previously published liver(1) snRNA-seq data with bulk RNA-seq data(2) of respective tissues. We used previously published WHRadjBMI(3) and MASLD(4) GWAS summary statistics for the MR analysis. *Cis*-eQTL indicates *cis*-expression trait locus; GWAS, genome-wide association study; MASLD, metabolic dysfunction-associated steatotic liver disease; SAT, subcutaneous adipose tissue; SNP, single nucleotide polymorphism; snRNA-seq, single nucleus RNA-sequencing; and WHRadjBMI, waist-to-hip ratio adjusted for body mass index.

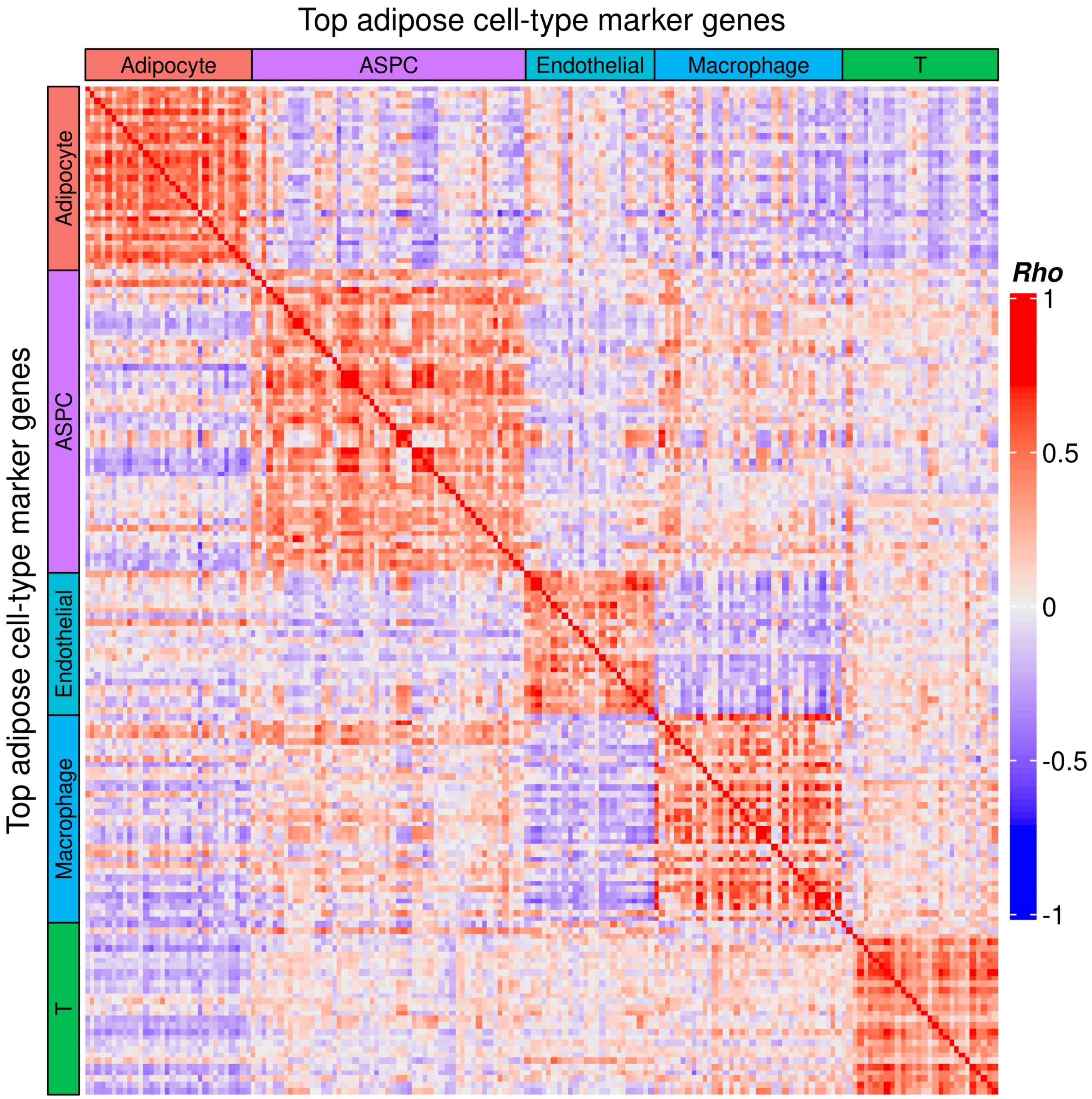

Supplementary Fig. 3. Subcutaneous adipose tissue cell-type marker genes are correlated within each cell-type. Pairwise gene-gene Spearman correlations of the top adipose tissue cell-type marker genes used by Bisque(5) for cell-type decomposition of adipose bulk RNA-sequencing data from 262 obese individuals in the KOBS cohort show high gene-gene correlations within each cell-type. The adipose cell-type marker genes were defined using adipose single nucleus RNA-sequencing data from 8 obese individuals in the KOBS cohort. ASPC indicates adipose stem and progenitor cells; and *Rho*, Spearman correlation coefficient.

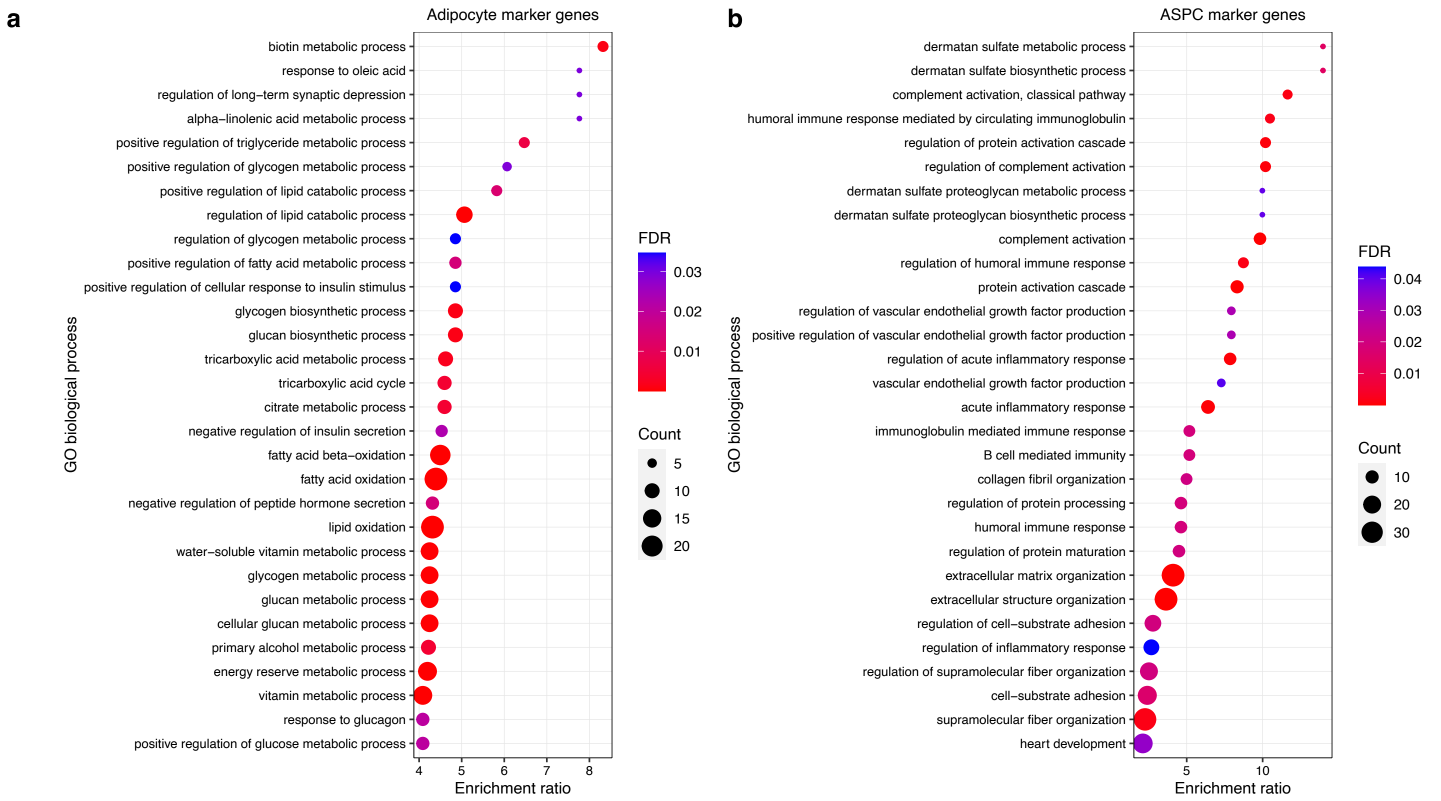

Supplementary Fig. 4. Adipose cell-type marker genes show significant functional enrichments. a,b, The marker genes of adipocyte (**a**) and ASPC (**b**) adipose tissue cell-types demonstrate significant (FDR<0.05) functional enrichments over the same number of randomly selected expressed genes in each respective cell-type, in which no functional enrichments were observed. The cell-type marker genes were defined using adipose single nucleus RNA-sequencing data from 8 obese individuals in the KOBS cohort. The dots represent significantly enriched biological processes from the Gene Ontology(6) database. Colors indicate FDR for the enrichment and the dot size represents the number of marker genes enriched in each biological process. The top 30 most strongly enriched biological processes, measured by enrichment ratio, are shown for each cell-type. ASPC indicates adipose stem and progenitor cells; FDR, false discovery rate; and GO, Gene Ontology.

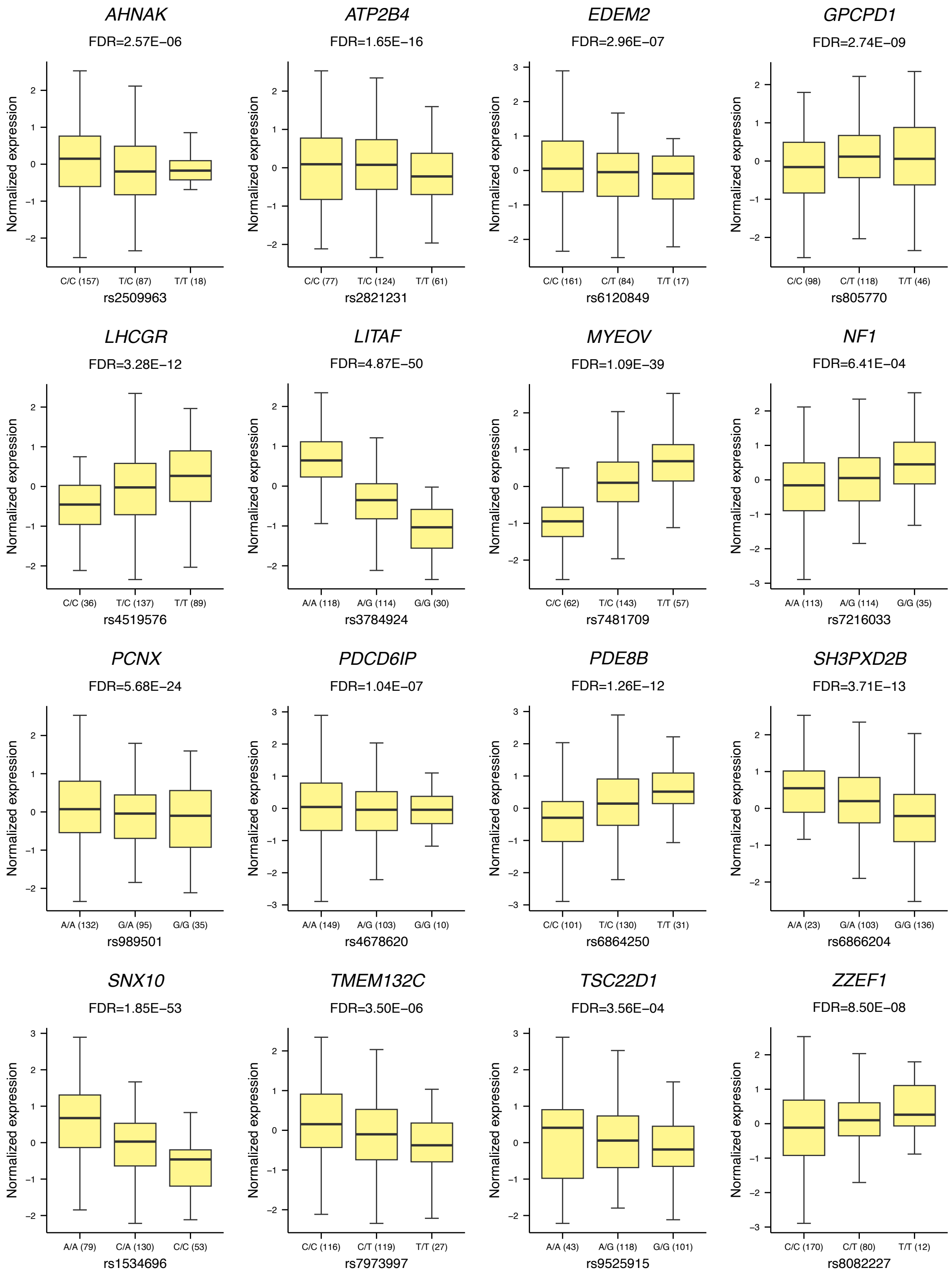

Supplementary Fig. 5. *Cis*-eQTL analysis identifies SNPs associated with adipose expression of subcutaneous adipose tissue cell-type marker genes. Boxplots show association between the genotypes of *cis*-eQTL SNPs and the normalized expression of adipose cell-type marker genes from 262 individuals in the KOBS cohort. The adipose cell-type marker genes were defined using adipose single nucleus RNA-sequencing data from 8 individuals in the KOBS cohort. The box shows the 25th and 75th percentiles with the whiskers extending to the 5th and 95th percentiles, and the center line shows the medians. The numbers in parentheses represent the numbers of individuals with the corresponding genotypes. The boxplot for the gene *PPP2R5A* is shown in Figure 1 of the manuscript. eQTL indicates expression quantitative trait locus; FDR, false discovery rate; and SNP, single nucleotide polymorphism.

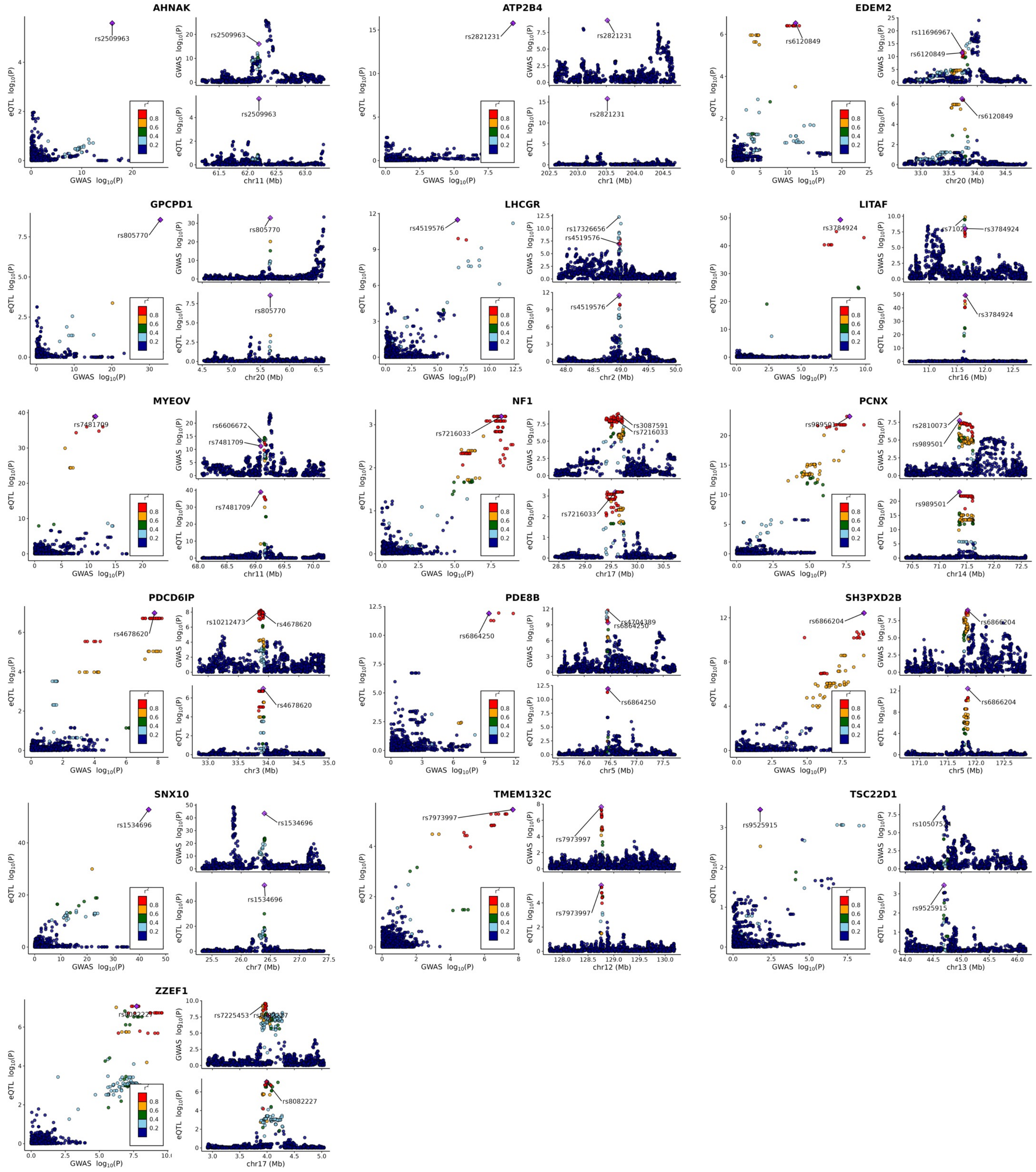

Supplementary Fig. 6. Regional overview showing the significantly colocalized WHRadjBMI GWAS cell-type-aware *cis*-eQTL SNPs regulating adipose cell-type marker genes. Comparisons of WHRadjBMI GWAS and adipose *cis*-eQTL SNPs (left panel) and regional overview of WHRadjBMI GWAS (top right panel) and adipose *cis*-eQTL loci (bottom right panel) demonstrate significant colocalizations of the WHRadjBMI GWAS and adipose *cis*-eQTL SNPs regulating adipose cell-type marker genes. The axes show the $-\log_{10}$ *p*-values from the GIANT and UK Biobank WHRadjBMI GWAS meta-analysis(3) and $-\log_{10}$ of *p*-values from the subcutaneous adipose *cis*-eQTL analysis in the KOBS cohort (*n*=262). The colocalized *cis*-eQTL SNP is represented by a purple diamond for each gene. The rsID number of the colocalized *cis*-eQTL SNP is shown in

all panels for each gene. If the colocalized GWAS SNP and *cis*-eQTL SNP are different, the rsID number of the colocalized GWAS SNP is also shown in the top right panel for each gene. The regional overview plot for the gene *PPP2R5A* is shown in Figure 1 of the manuscript. The primary *cis*-eQTL SNPs for *AHNAK*, *ATP2B4*, *EDEM2*, *LHCGR*, *LITAF*, *NF1*, *PCNX*, *PDCD6IP*, *PDE8B*, *PPP2R5A*, *SH3PXD2B*, *TMEM132C*, *TSC22D1*, and *ZZEF1* colocalized with the lead GWAS SNPs or SNPs in tight LD (R^2>0.8) with the lead GWAS SNPs. The primary *cis*-eQTL SNPs for *GPCPD1*, *MYEOV*, and *SNX10* colocalized with the secondary GWAS SNPs after fine-mapping using COLOC-SuSiE(7). Colors represent LD (R^2) with the colocalized *cis*-eQTL SNP. eQTL indicates expression quantitative trait locus; GWAS, genome-wide association study; LD, linkage disequilibrium; rsID, reference single nucleotide polymorphism identification number; SNP, single nucleotide polymorphism; and WHRadjBMI, waist-to-hip ratio adjusted for body mass index.

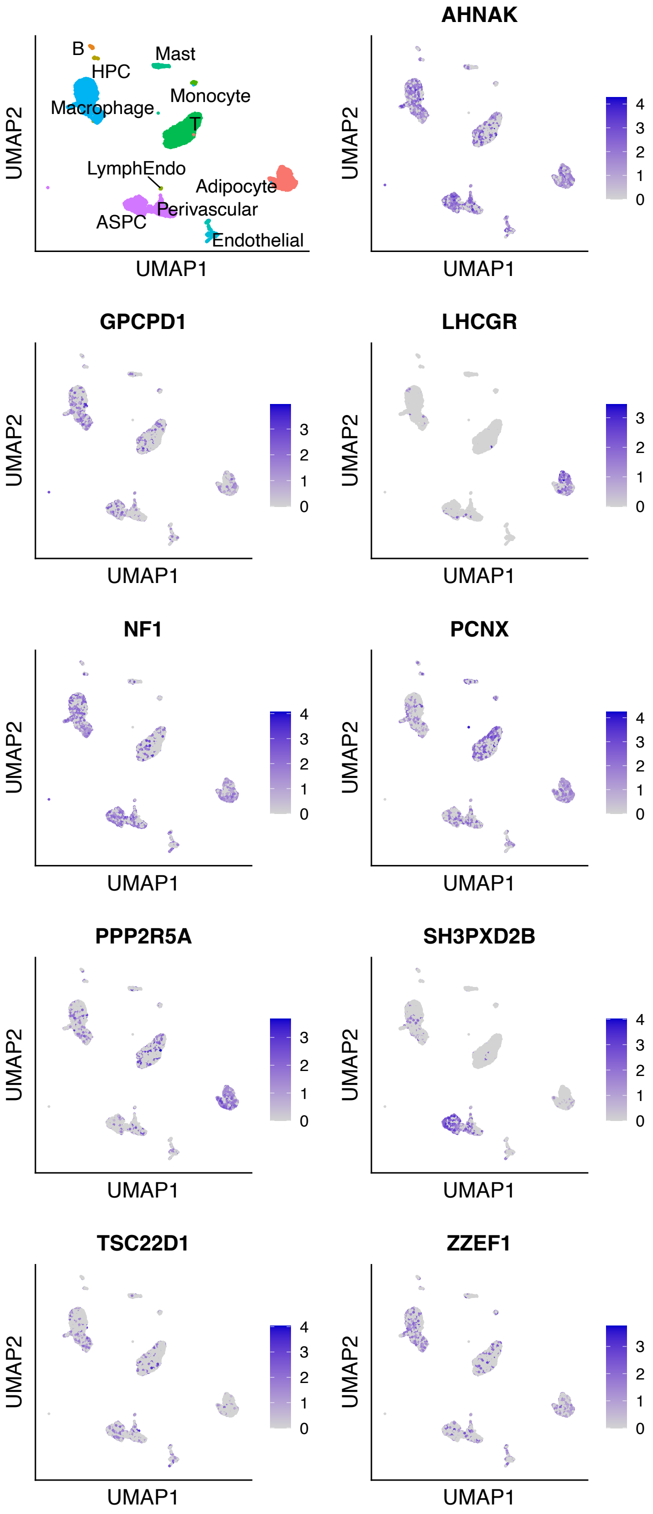

Supplementary Fig. 7. Single-nucleus RNA-sequencing identifies subcutaneous adipose tissue cell-types expressing adipose cell-type-aware WHRadjBMI GWAS *cis*-eQTL target genes. UMAP shows 11 cell-type clusters in single nucleus RNA-seq (snRNA-seq) data of subcutaneous adipose tissue from 8 obese KOBS participants. Colors represent nuclei expression of the adipose cell-type-aware WHRadjBMI GWAS *cis*-eQTL target genes identified by the colocalization analysis of WHRadjBMI GWAS and subcutaneous adipose tissue *cis*-eQTL data from the adipose cell-type marker genes. Expression values are gene counts normalized by total counts per cell, multiplied by a scaling factor, and then log transformed. ASPC indicates adipose stem and progenitor cells; eQTL, expression quantitative trait locus; GWAS, genome-wide association study; HPC, hematopoietic stem cell; LymphEndo, lymphatic endothelial cells; UMAP, Uniform Manifold Approximation and Projection; and WHRadjBMI, waist-to-hip ratio adjusted for body mass index.

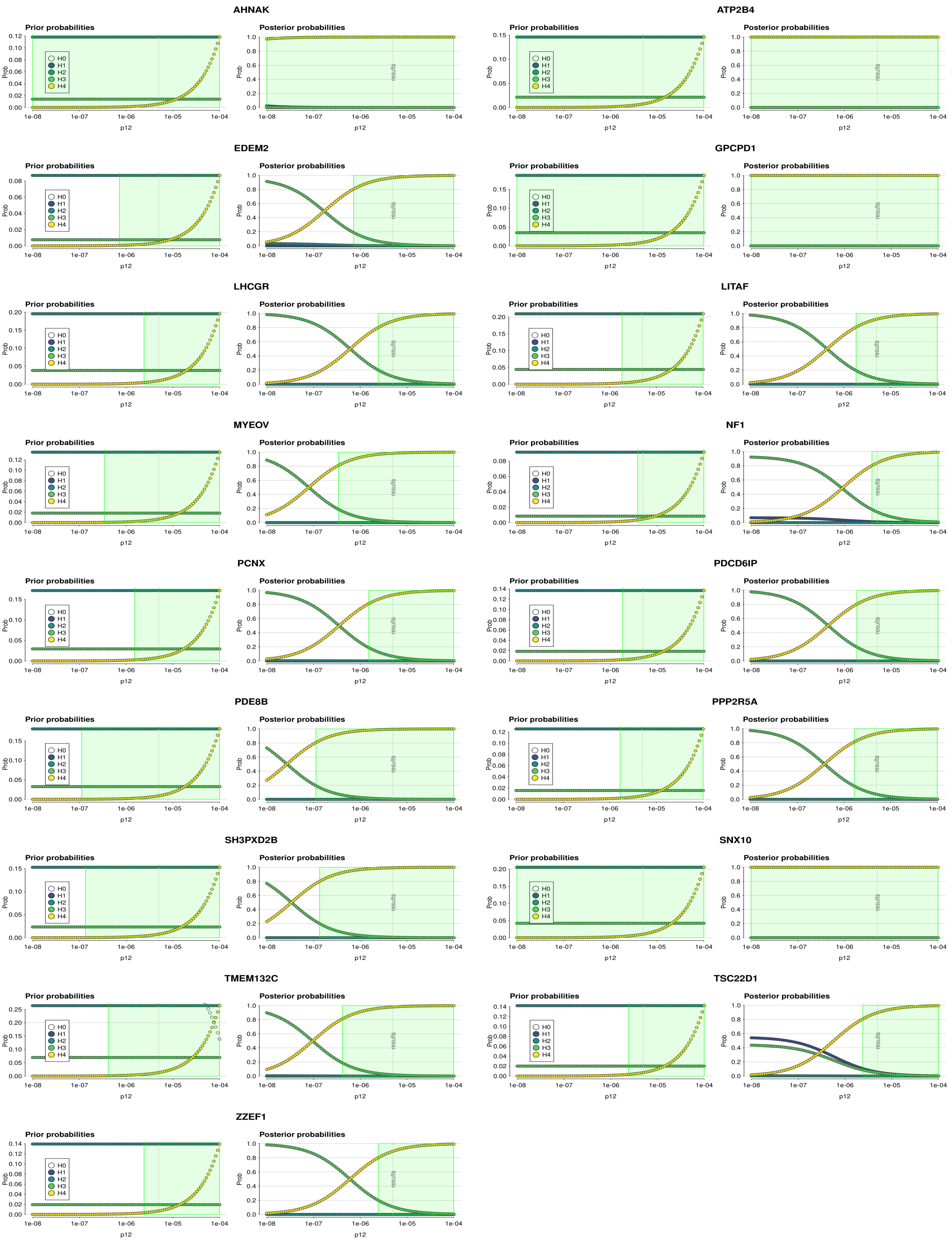

Supplementary Fig. 8. A sensitivity analysis shows robustness of the colocalized variants to prior probability used in the colocalization analysis. Each dot shows the prior probability (left panel) and posterior probability (right panel) for each hypothesis under the range of prior probabilities (p_{12}) used for the colocalization analysis. Colors represent the hypothesis that variants have no association with either GWAS or *cis*-eQTL in region (H_0), association with GWAS but not with *cis*-eQTL (H_1), association with *cis*-eQTL but not with GWAS

(H_2), association with both GWAS and *cis*-eQTL but with separate causal variants (H_3), and association with both GWAS and *cis*-eQTL with same causal variants (H_4), respectively. p12 indicates prior probability that a variant is jointly associated with both GWAS and *cis*-eQTL. A vertical dashed line is shown for the prior probability ($p_{12}=5\times10^{-6}$) used in the main analysis. A range of prior probabilities (p_{12}) that shows evidence for colocalization (posterior probability $H_4>0.8$) is highlighted in green. eQTL indicates expression quantitative trait locus; and GWAS, genome-wide association study.

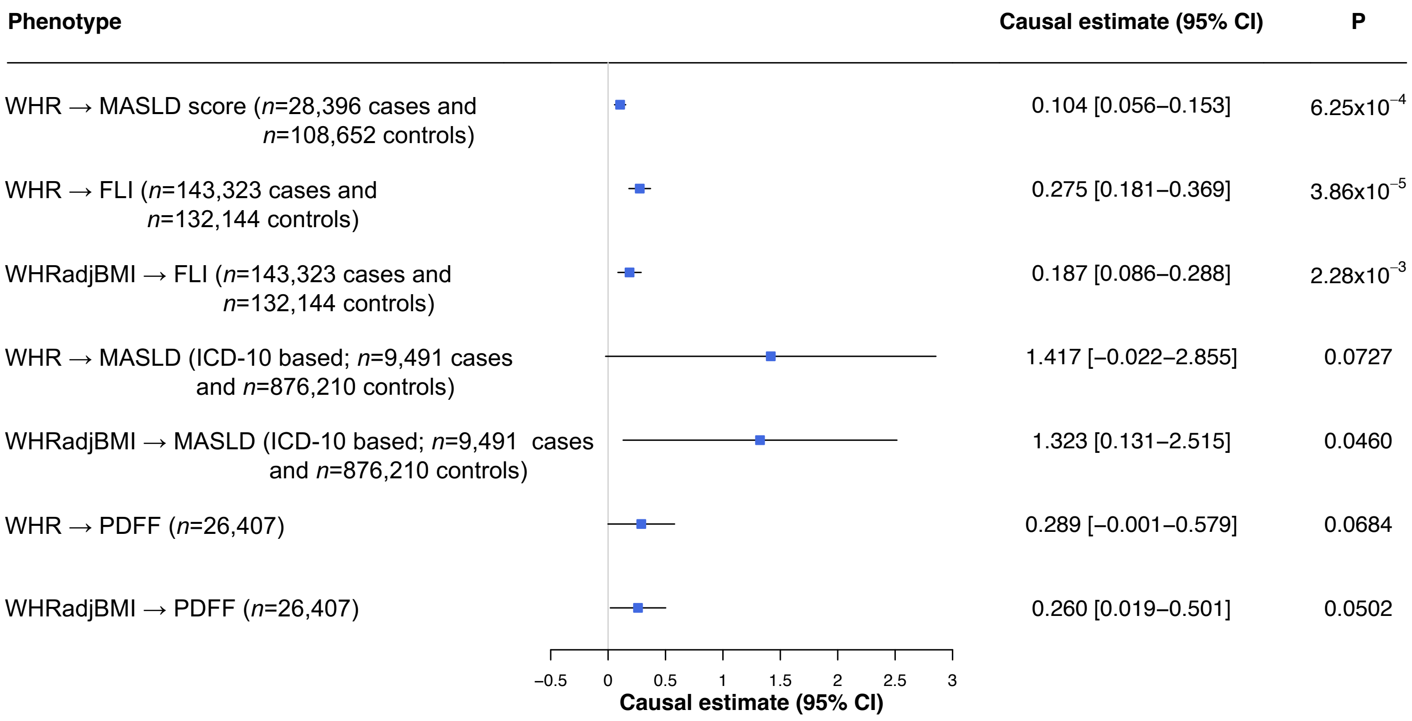

Supplementary Fig. 9. Mendelian randomization (MR) demonstrates a putative causal effect of abdominal obesity on MASLD using multiple surrogate measurements. A putative causal effect of abdominal obesity on MASLD by MR-PRESSO(8) is shown using WHR and WHRadjBMI as the exposure traits and MASLD score(4) , the fatty liver index (FLI)(9), meta-analysis of the International Classification of Disease, 10th revision (ICD-10) based MASLD status(10), and liver magnetic resonance imaging (MRI) proton density fat fraction (PDFF) as the outcome traits. The adipose cell-type-aware WHRadjBMI GWAS *cis*-eQTL SNPs (*n*=17) were used as instrumental variables with MR-. One SNP detected as an outlier by MR-PRESSO was removed for WHR→FLI, WHR→MASLD (ICD-10 based), and WHRadjBMI→MASLD (ICD-10 based) phenotypes. CI indicates confidence interval; MASLD, metabolic dysfunction-associated steatotic liver disease; WHRadjBMI, waist-to-hip ratio; and WHRadjBMI, waist-to-hip ratio adjusted for body mass index.

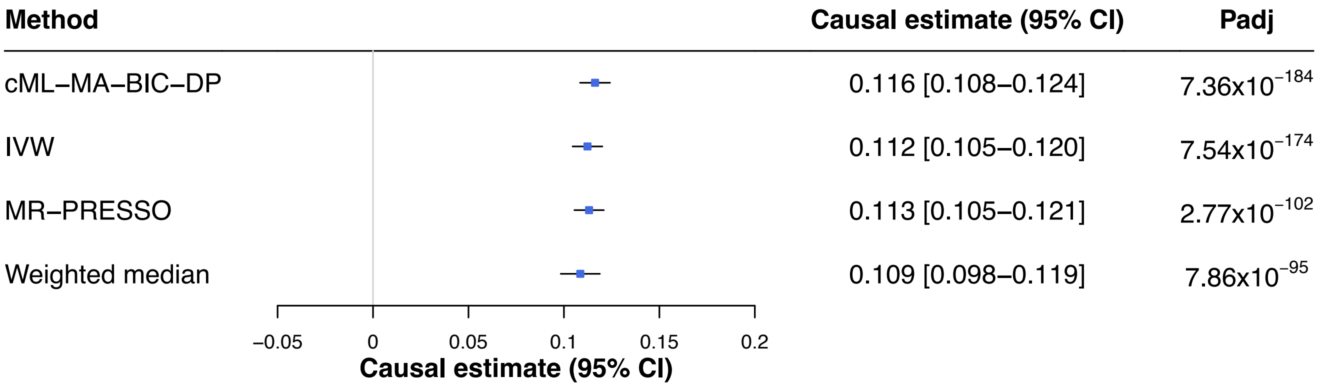

Supplementary Fig. 10. Mendelian randomization (MR) demonstrates a significant heterogeneity in the putative causal effect of WHRadjBMI on MASLD when using all non-redundant WHRadjBMI GWAS SNPs. A putative causal effect of WHRadjBMI on MASLD is shown using all independent (R^2>0.0.001; *n*=442) WHRadjBMI GWAS SNPs(3). Significant MASLD GWAS SNPs(4), SNPS in tight LD (R^2>0.0.001) with MASLD GWAS SNPs, and SNPs detected as outliers using MR-PRESSO(8) were excluded. Causal estimates (beta) with 95% confidence intervals and *p*-values computed using cML-MA-BIC-DP(11), IVW(12), MR-PRESSO(8), and median weighted(13) are shown. Cochran's Q test(14) (Q=730, *p*-value=1.34×10^{-16}) shows a significant heterogeneity among the instrumental variables (IVs) used for the MR analysis. CI indicates confidence interval; GWAS, genome-wide association study; IVW, inverse variance weighting; LD, linkage disequilibrium; MASLD, metabolic dysfunction-associated steatotic liver disease; nSNP, number of single nucleotide polymorphisms; Padj, Bonferroni adjusted *p*-value; and WHRadjBMI, waist-to-hip ratio adjusted for body mass index.

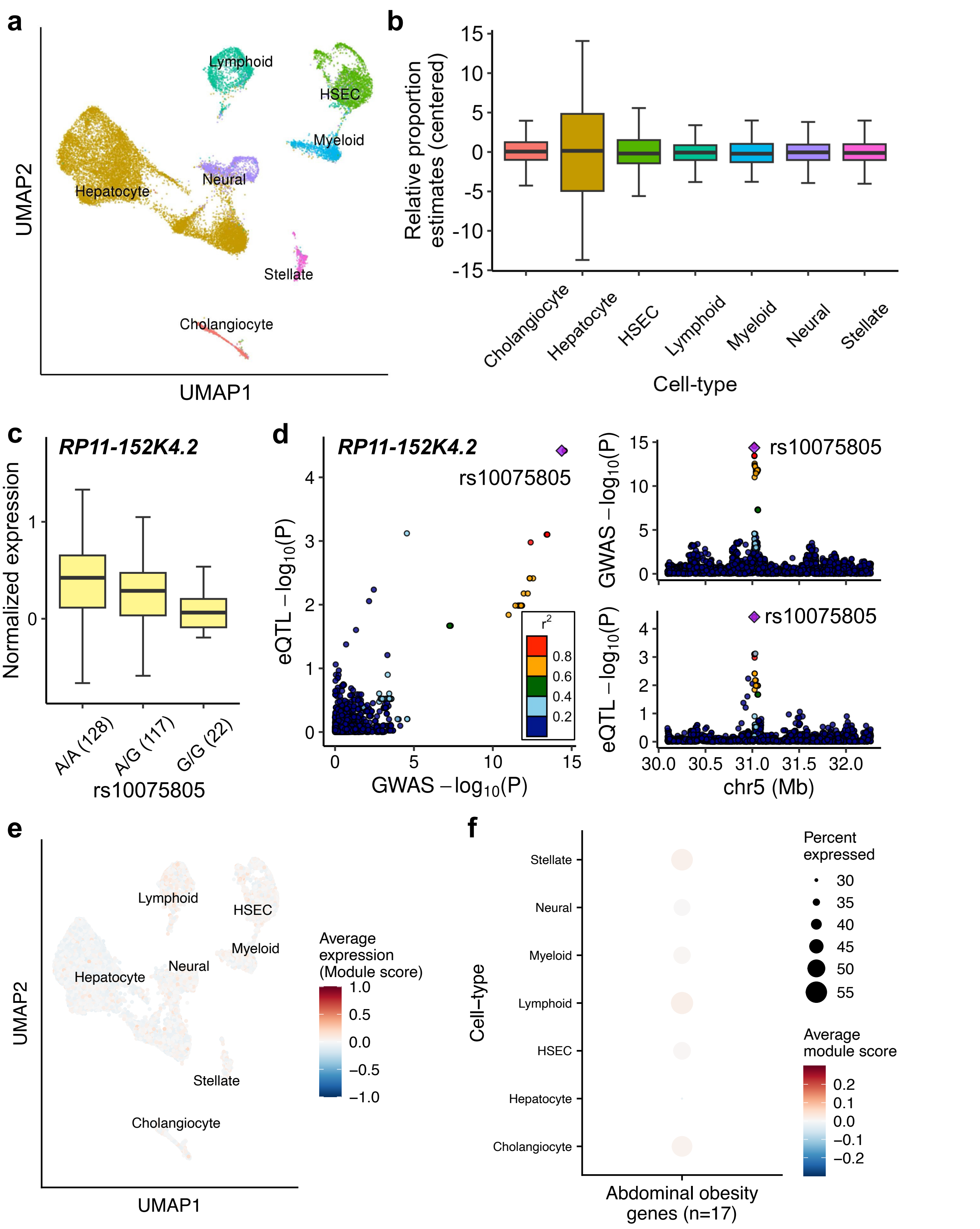

Supplementary Fig. 11. Single-nucleus RNA-sequencing and colocalization analyses identify a liver cell-type-aware MASLD GWAS *cis*-eQTL variant. a, UMAP shows 7 cell-type clusters in single nucleus RNA-sequencing (snRNA-seq) data of liver tissue from 3 individuals. HSEC indicates hepatic sinusoidal endothelial cells; and UMAP, Uniform Manifold Approximation and Projection. **b**, Liver cell-type proportions were estimated in the bulk RNA-seq data from 272 obese individuals in the KOBS cohort using Bisque(5). The box shows the 25th and 75th percentiles with the whiskers extending to the 5th and 95th percentiles, and the center line

shows the medians. **c**, *Cis*-eQTL variant rs10075805 targets the gene *RP11-152K4.2*. Boxplots show an association between the genotypes of rs10075805 and the normalized expression of the liver cell-type marker gene *RP11-152K4.2* from the liver bulk RNA-seq data (n=272 individuals) in the KOBS cohort. The box shows the 25th and 75th percentiles with the whiskers extending to the 5th and 95th percentiles, and the center line shows the medians. The numbers in parentheses represent the numbers of individuals with the corresponding genotypes. eQTL indicates expression quantitative trait locus. **d**, Comparison of MASLD GWAS and liver *cis*-eQTL SNPs (left panel) and regional overview of MASLD GWAS (top right panel) and liver *cis*-eQTL loci (bottom right panel) demonstrate a significant colocalization of the MASLD GWAS and liver *cis*-eQTL SNP rs10075805 targeting the gene *RP11-152K4.2*. The axes show the −log10 of *p*-values from the MASLD GWAS(4) and −log10 of *p*-values from the liver *cis*-eQTL analysis in the KOBS cohort (*n*=272). The colocalized *cis*-eQTL SNP, rs10075805, is represented by a purple diamond. Colors represent LD (R^2) with colocalized *cis*-eQTL SNP. **e,** UMAP illustration of the liver cell-type clusters where each dot represents a nucleus colored by the module score of the 17 abdominal obesity genes. **f,** Dot plot shows no enrichment of the module score in any liver cell-types. The size of each dot represents the percent of cells with a module score >0 in each cell-type and the colors represent an average module score for each cell-type. Chr indicates chromosome; GWAS, genome-wide association study; LD, linkage disequilibrium; HSEC, hepatic sinusoidal endothelial cells; Mb, mega base; MASLD, metabolic dysfunction-associated steatotic liver disease; P, *p*-value; SNP, single nucleotide polymorphism; and UMAP, Uniform Manifold Approximation and Projection.

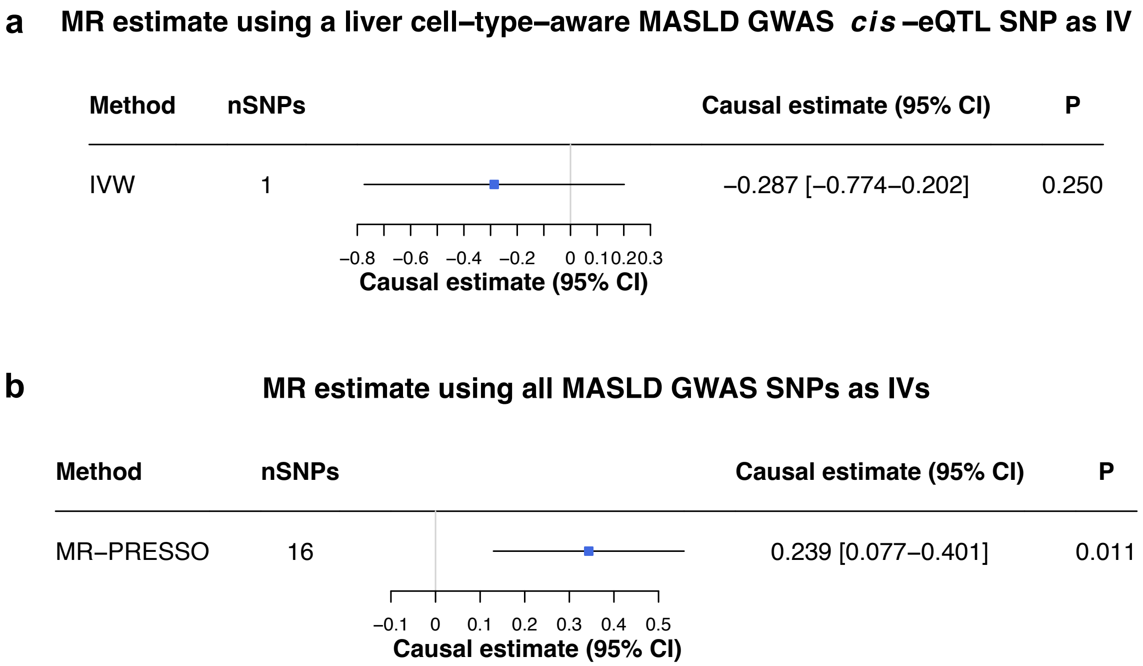

Supplementary Fig. 12. Mendelian randomization (MR) demonstrates significant heterogeneity in the putative causal effect of MASLD on WHRadjBMI. a,b Putative causal effects of MASLD on WHRadjBMI are shown using a liver cell-type-aware MASLD GWAS *cis*-eQTL SNP regulating the liver cell-type marker gene *RP11-152K4.2* (**a**) or all independent (R^2>0.001) MASLD GWAS SNPs(4) as the instrumental variables (IVs) (**b**). Significant WHRadjBMI GWAS SNPs(3) and SNPs in tight LD (R^2>0.001) with WHRadjBMI GWAS SNPs were excluded. **a**, As only 1 SNP was used as a single IV, the causal estimate (beta) with 95% confidence intervals and *p*-values were computed using the IVW method(12) and horizontal pleiotropy and heterogeneity were not evaluated. **b**, The causal estimate (beta) with 95% confidence intervals and *p*-value computed using MR-PRESSO(8) are shown. Cochran's Q test(14) (Q=33.53, *p*-value=0.004) shows significant heterogeneity among the instrumental variables (IVs) used for the MR analysis. CI indicates confidence interval; GWAS, genome-wide association study; IVW, inverse variance weighted; LD, linkage disequilibrium; MASLD, metabolic dysfunction-associated steatotic liver disease; nSNPs, number of single nucleotide polymorphisms; P, *p*-value; and WHRadjBMI, waist-to-hip ratio adjusted for body mass index.

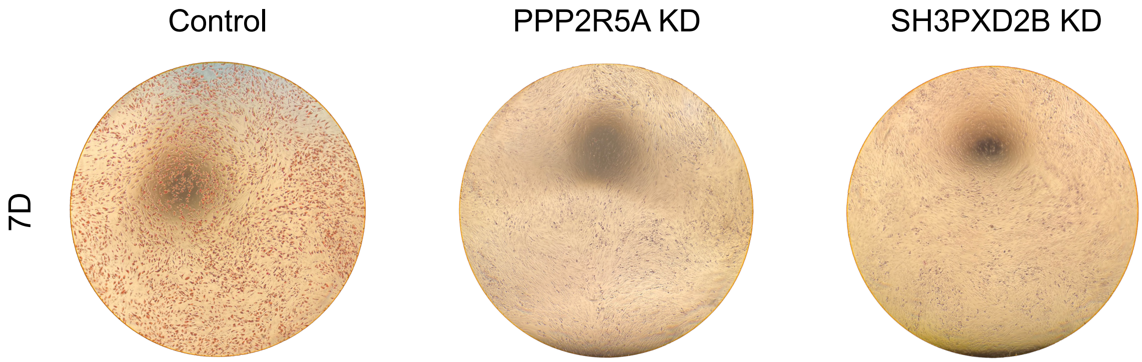

Supplementary Fig. 13. Oil Red O (ORO) lipid staining shows impaired lipid accumulation with the knockdown of *PPP2R5A* and *SH3PXD2B* in human SGBS preadipocytes. Human SGBS preadipocytes with siRNA-mediated knockdown (KD) of *PPP2R5A* and *SH3PXD2B* in the differentiation media were stained with ORO for each condition at three time points. Cell images shown here are taken at the 7-days (7D) time point using the EVOS Core XL microscope at 5x zoom to visualize the full extent of the differentiation and lipidation. Row indicates the number of days from initiating the differentiation of preadipocytes. Columns indicate the experimental conditions as follows: non-transfected controls, *PPP2R5A* knockdown (60 nM), and *SH3PXD2B* knockdown (150 nM). The cell images taken at 20x zoom for more detailed visualization from all conditions and time points are shown in Figure 5a.

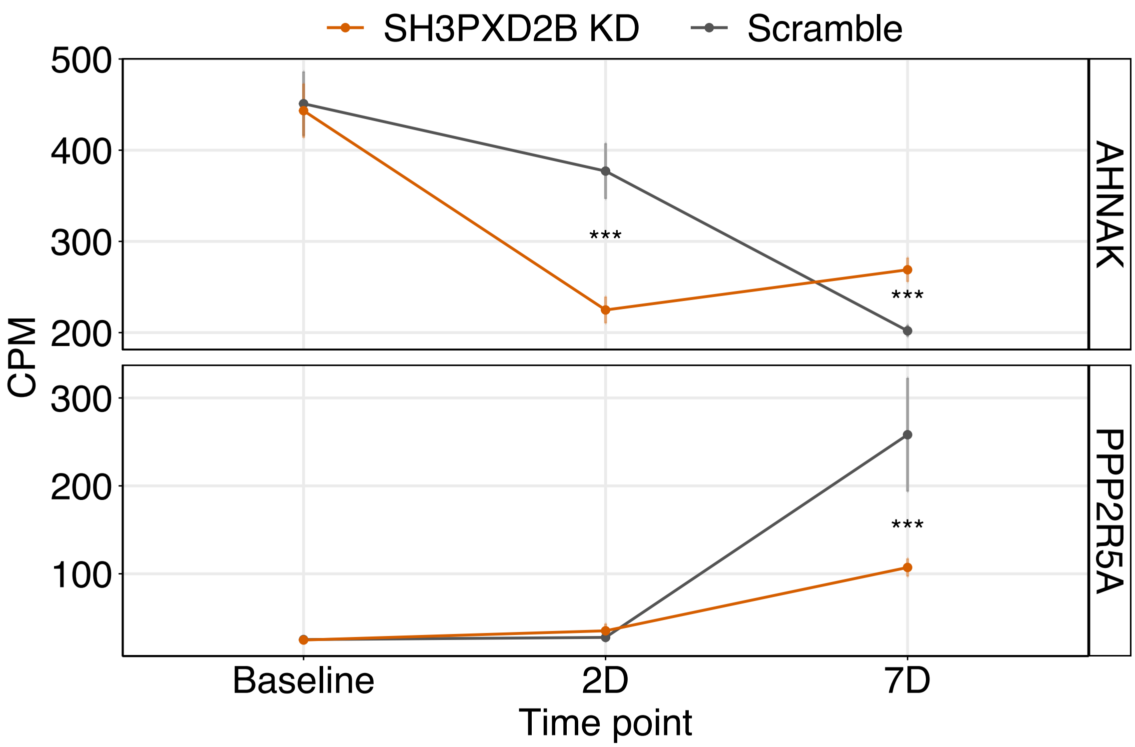

Supplementary Fig. 14. ***SH3PXD2B*** **knockdown in human SGBS preadipocytes affects expression of three other abdominal obesity adipocyte and ASPC marker genes.** Average expression, in counts per million (CPM), of the adipocyte and ASPC marker genes *AHNAK*, and *PPP2R5A,* among the 17 abdominal obesity genes, are shown for the *SH3PXD2B* knockdown and scrambled control samples at each time point. Dots represent average CPMs from 2-4 technical replicates with error bars indicating ± standard deviation. Time point is represented by the x-axis and the average expression (CPM) by the y-axis. The colors represent average expression in the *SH3PXD2B* knockdown *vs* respective scrambled controls. Significant differences in the average expression of each gene between the *SH3PXD2B* knockdown and scrambled controls samples at each time point are shown (*, *p*-value<0.05; **, *p*-value<0.01; ***, *p*-value<0.001) by the t-test.

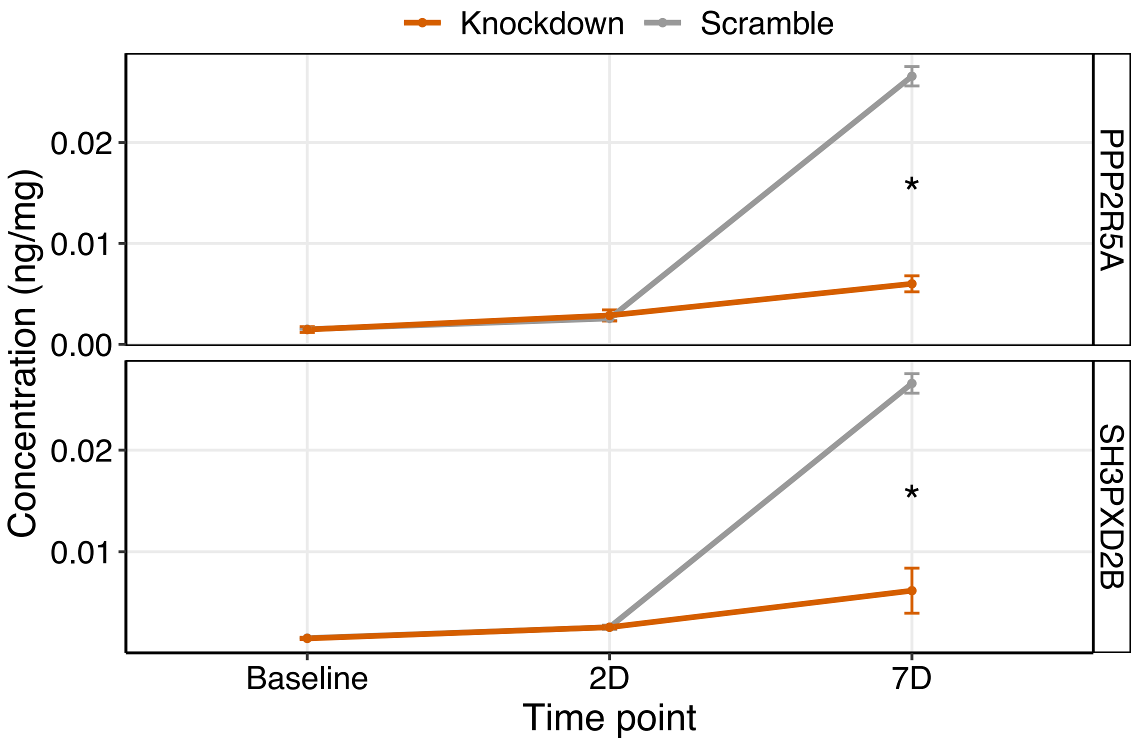

Supplementary Fig. 15. *PPP2R5A* and *SH3PXD2B* knockdowns in human SGBS preadipocytes affect serum adiponectin levels during adipogenesis. The secretion of the adiponectin protein by the *PPP2R5A* and *SH3PXD2B* knockdown and the scramble control cells were quantified during adipocyte differentiation. Dots represent the concentration of the adiponectin in the cell culture supernatant that was determined by interpolating against a standard curve and then normalized based on the total protein content. The error bars indicate ± standard deviation and the colors represent the condition. Significant differences in the adiponectin measurements between the knockdown and scrambled control samples are shown (*, p-value<0.05) by the t-test.

Supplementary Table 1. Summary report of the ATAC-seq QC metrics in the human primary preadipocyte differentiation experiment.

QC metric	Mean	SD	Range
**FRiP**	0.43	0.10	0.20 - 0.65
**NRF**	0.61	0.04	0.51 - 0.70
**PBC1**	0.70	0.03	0.62 - 0.74
**PBC2**	3.7	0.4	2.7 - 4.4
**Final read count**	90,357,088	31,428,074	33,062,460 – 146,940,751
**Fraction of reads uniquely aligned**	0.73	0.04	0.67 - 0.81
**Fraction of reads aligned to Mt**	0.006	0.002	0.002 - 0.009
**Median insert size (bp)**	196	35	136 - 268

ATAC-seq indicates assay for transposase accessible chromatin sequencing; Bp, base pair; FRiP, fraction of reads in called peak regions; Mt, mitochondria; NRF, non-redundant fraction; PBC1 and PBC2, polymerase chain reaction (PCR) bottlenecking coefficients 1 and 2; QC, quality control; and SD, standard deviation.

Supplementary Table 2. Summary of the KOBS SAT bulk RNA-seq cohort phenotypes.

Cohort	Number of males	Number of females	Age (mean±SD)	BMI (mean±SD)
**KOBS SAT bulk RNA-seq**	80	182	48.6±9.1	43.1±5.3

SD indicates standard deviation.

Supplementary Table 3. Subcutaneous adipose tissue cell-type marker genes identified by snRNA-seq in the KOBS participants with severe obesity are ranked by the Bonferroni adjusted *p*-value (please see the Supplementary Table 3 excel table).

Supplementary Table 4. Liver cell-type marker genes identified by snRNA-seq are ranked by the Bonferroni adjusted *p*-value (please see the Supplementary Table 4 excel table).

Supplementary Table 5. The adipose cell-type-aware WHRadjBMI GWAS *cis*-eQTL SNPs targeting adipocyte and ASPC marker genes are differentially expressed between the differentiating human preadipocytes measured at the baseline and 7 days after initiation of the differentiation as well as longitudinally across the adipogenesis experiment.

Cell-type	Gene	Baseline *vs* 7-day time points DE*			Longitudinal DE†	
		logFC	P	adj.P	P	adj.P
Adipocyte	LHCGR	3.73	8.73×10^{-8}	1.75×10^{-7}	3.67×10^{-15}	4.59×10^{-15}
	MYEOV	-3.42	8.52×10^{-5}	9.47×10^{-5}	2.55×10^{-4}	2.55×10^{-4}
	PDE8B	8.46	9.35×10^{-7}	1.56×10^{-6}	2.70×10^{-147}	5.39×10^{-147}
	PPP2R5A	1.33	1.53×10^{-8}	3.83×10^{-8}	2.44×10^{-218}	1.22×10^{-217}
	TMEM132C	10.61	1.17×10^{-10}	1.17×10^{-9}	$<2.23\times10^{-308}$	$<2.23\times10^{-308}$
ASPC	AHNAK	-0.63	3.12×10^{-6}	4.45×10^{-6}	2.35×10^{-74}	3.91×10^{-74}
	EDEM2	-0.60	1.16×10^{-5}	1.45×10^{-5}	6.94×10^{-45}	9.92×10^{-45}
	NF1	0.23	1.89×10^{-3}	1.89×10^{-3}	1.43×10^{-4}	1.59×10^{-4}
	SH3PXD2B	1.68	5.50×10^{-10}	2.75×10^{-9}	6.82×10^{-215}	2.27×10^{-214}
	TSC22D1	1.35	1.15×10^{-9}	3.82×10^{-9}	1.30×10^{-147}	3.25×10^{-147}

*The expression of the 10 human subcutaneous adipose tissue adipocyte and ASPC marker genes that are adipose cell-type-aware WHRadjBMI GWAS *cis*-eQTL eGenes were tested for DE in the differentiating human preadipocytes between the baseline and 7-day time points using limma(15). P-values were corrected for multiple testing using Bonferroni.

†The expression of the 10 human subcutaneous adipose tissue adipocyte and ASPC marker genes that are adipose cell-type-aware WHRadjBMI GWAS *cis*-eQTL eGenes were tested for longitudinal DE in differentiating human preadipocytes across the 6 time points over 14 days (baseline, 1-day, 2-day, 4-day, 7-day, and 14-day) using ImpulseDE2(16). P-values were corrected for multiple testing using false discovery rate (FDR).

ASPC indicates adipose stem and progenitor cells; adj.P, multiple testing adjusted *p*-value; DE, differential expression; eQTL, expression quantitative trait locus; GWAS, genome-wide association study; logFC, log fold change; P, *p*-value; and WHRadjBMI, waist-to-hip ratio adjusted for body mass index.

Supplementary Table 6. Clustering of the adipocyte and ASPC marker genes regulated by the adipose cell-type-aware WHRadjBMI GWAS *cis*-eQTL SNPs based on similar longitudinal expression trajectories in the differentiating human preadipocytes using the DPGP tool(17)**.**

Cluster	Gene name	Cluster assignment probability
1	AHNAK	1.00
1	MYEOV	1.00
2	EDEM2	1.00
3	LHCGR	0.85
3	NF1	0.92
3	SH3PXD2B	0.92
4	PDE8B	0.97
4	PPP2R5A	0.96
4	TMEM132C	0.96
4	TSC22D1	0.94

Supplementary Table 7. The adipocyte and ASPC marker genes regulated by the adipose cell-type-aware WHRadjBMI GWAS *cis*-eQTL SNPs are differentially expressed between the differentiating human SGBS preadipocytes measured at the baseline and 7 days after initiation of the differentiation as well as longitudinally across the adipogenesis experiment.

Cell-type	Gene	Baseline *vs* 7-day time points DE*			Longitudinal DE†	
		logFC	P	adj.P	P	adj.P
Adipocyte	MYEOV	-5.52	1.01×10^{-5}	2.02×10^{-5}	1.10×10^{-11}	2.94×10^{-11}
	PDE8B	5.31	1.78×10^{-7}	1.43×10^{-6}	5.60×10^{-50}	2.24×10^{-49}
	PPP2R5A	3.52	8.35×10^{-6}	2.02×10^{-5}	3.87×10^{-90}	3.10×10^{-89}
ASPC	AHNAK	-1.07	5.11×10^{-6}	2.02×10^{-5}	3.50×10^{-11}	6.99×10^{-11}
	EDEM2	-0.20	0.261	0.261	0.224	0.256
	NF1	-0.91	3.23×10^{-3}	4.23×10^{-3}	3.05×10^{-5}	4.07×10^{-5}
	SH3PXD2B	0.69	3.70×10^{-3}	4.23×10^{-3}	4.81×10^{-6}	7.69×10^{-6}
	TSC22D1	0.41	9.43×10^{-5}	1.51×10^{-4}	0.998	0.998

*The expression of the 10 human subcutaneous adipose tissue adipocyte and ASPC marker genes that are adipose cell-type-aware WHRadjBMI GWAS *cis*-eQTL eGenes were tested for DE in the differentiating human SGBS preadipocytes between the baseline and 7-day time points using limma(15). P-values were corrected for multiple testing using Bonferroni.

†The expression of the 10 human subcutaneous adipose tissue adipocyte and ASPC marker genes that are adipose cell-type-aware WHRadjBMI GWAS *cis*-eQTL eGenes were tested for longitudinal DE in differentiating human SGBS preadipocytes across the 6 time points over 7 days (baseline, 2-day, and 7-day) using ImpulseDE2(16). P-values were corrected for multiple testing using false discovery rate (FDR).

ASPC indicates adipose stem and progenitor cells; adj.P, multiple testing adjusted *p*-value; DE, differential expression; eQTL, expression quantitative trait locus; GWAS, genome-wide association study; logFC, log fold change; P, *p*-value; and WHRadjBMI, waist-to-hip ratio adjusted for body mass index.

Supplementary Table 8. Clustering of the adipocyte and ASPC marker genes regulated by the adipose cell-type-aware WHRadjBMI GWAS cis-eQTL SNPs based on similar longitudinal expression trajectories in the differentiating human SGBS preadipocytes using the DPGP tool(17).

Cluster	Gene name	Cluster assignment probability
1	AHNAK	1.00
1	EDEM2	1.00
1	MYEOV	1.00
1	NF1	1.00
2	PDE8B	1.00
2	PPP2R5A	1.00
2	TSC22D1	1.00
3	SH3PXD2B	1.00

Supplementary Table 9. Key lipid droplet genes, *PLIN1* and *PLIN4*, are already highly expressed at Day 7 in both the human primary preadipocyte and human SGBS preadipocyte adipogenesis experiments.

Experiment	*PLIN1* baseline *vs* 7-day time points*			*PLIN4* baseline *vs* 7-day time points		
	Day 0	Day 7	P	Day 0	Day 7	P
Human primary predipocytes	0.031	733.4	8.924×10^{-5}	0.458	505.0	1.081×10^{-4}
SGBS	0.129	698.4	9.805×10^{-3}	0.295	516.6	0.035

*The expression of the key lipid droplet genes, *PLIN1* and *PLIN4*, were tested for DE between the baseline and 7-day time points of preadipocyte differentiation in two independent human adipogenesis experiments. Student's t-test was performed on the normalized average CPMs from 3 technical replicates at each time point.

Supplementary Table 10. Significant (adjusted p-value<0.05) differential expression (DE) analysis results from the siRNA-mediated knockdown of *PPP2R5A* in human SGBS preadipocytes cultured for differentiation and collected for bulk RNA-sequencing at 3 time points are ranked by log fold-change in the knockdown compared to the scrambled controls for each time point (please see the Supplementary Table 10 excel table).

Supplementary Table 11. Significant (adjusted p-value<0.05) differential expression (DE) analysis results from the siRNA-mediated knockdown of *SH3PXD2B* in human SGBS preadipocytes cultured for differentiation and collected for bulk RNA-sequencing at 3 time points are ranked by log fold-change in the knockdown compared to the scrambled controls for each time point (please see the Supplementary Table 11 excel table).

Supplementary References

1. Rao S, Yang X, Ohshiro K, Zaidi S, Wang Z, Shetty K, et al. β2-spectrin (SPTBN1) as a therapeutic target for diet-induced liver disease and preventing cancer development. Sci Transl Med. 2021 Dec 15;13(624):eabk2267.

2. van der Kolk BW, Muniandy M, Kaminska D, Alvarez M, Ko A, Miao Z, et al. Differential Mitochondrial Gene Expression in Adipose Tissue Following Weight Loss Induced by Diet or Bariatric Surgery. J Clin Endocrinol Metab. 2021 Apr 23;106(5):1312–24.

3. Pulit SL, Stoneman C, Morris AP, Wood AR, Glastonbury CA, Tyrrell J, et al. Meta-analysis of genome-wide association studies for body fat distribution in 694 649 individuals of European ancestry. Hum Mol Genet. 2019 Jan 1;28(1):166–74.

4. Miao Z, Garske KM, Pan DZ, Koka A, Kaminska D, Männistö V, et al. Identification of 90 NAFLD GWAS loci and establishment of NAFLD PRS and causal role of NAFLD in coronary artery disease. Hum Genet Genomics Adv. 2022 Jan;3(1):100056.

5. Jew B, Alvarez M, Rahmani E, Miao Z, Ko A, Garske KM, et al. Accurate estimation of cell composition in bulk expression through robust integration of single-cell information. Nat Commun. 2020 Apr 24;11(1):1971.

6. Ashburner M, Ball CA, Blake JA, Botstein D, Butler H, Cherry JM, et al. Gene ontology: tool for the unification of biology. The Gene Ontology Consortium. Nat Genet. 2000 May;25(1):25–9.

7. Wallace C. A more accurate method for colocalisation analysis allowing for multiple causal variants. Cordell HJ, editor. PLOS Genet. 2021 Sep 29;17(9):e1009440.

8. Verbanck M, Chen CY, Neale B, Do R. Detection of widespread horizontal pleiotropy in causal relationships inferred from Mendelian randomization between complex traits and diseases. Nat Genet. 2018 May;50(5):693–8.

9. Bedogni G, Bellentani S, Miglioli L, Masutti F, Passalacqua M, Castiglione A, et al. The Fatty Liver Index: a simple and accurate predictor of hepatic steatosis in the general population. BMC Gastroenterol. 2006 Nov 2;6:33.

10. Sveinbjornsson G, Ulfarsson MO, Thorolfsdottir RB, Jonsson BA, Einarsson E, Gunnlaugsson G, et al. Multiomics study of nonalcoholic fatty liver disease. Nat Genet. 2022 Nov;54(11):1652–63.

11. Xue H, Shen X, Pan W. Constrained maximum likelihood-based Mendelian randomization robust to both correlated and uncorrelated pleiotropic effects. Am J Hum Genet. 2021 Jul;108(7):1251–69.

12. Burgess S, Butterworth A, Thompson SG. Mendelian Randomization Analysis With Multiple Genetic Variants Using Summarized Data. Genet Epidemiol. 2013 Nov;37(7):658–65.

13. Bowden J, Davey Smith G, Haycock PC, Burgess S. Consistent Estimation in Mendelian Randomization with Some Invalid Instruments Using a Weighted Median Estimator. Genet Epidemiol. 2016 May;40(4):304–14.

14. Bowden J, Del Greco M F, Minelli C, Zhao Q, Lawlor DA, Sheehan NA, et al. Improving the accuracy of two-sample summary-data Mendelian randomization: moving beyond the NOME assumption. Int J Epidemiol. 2019 Jun 1;48(3):728–42.

15. Ritchie ME, Phipson B, Wu D, Hu Y, Law CW, Shi W, et al. limma powers differential expression analyses for RNA-sequencing and microarray studies. Nucleic Acids Res. 2015 Apr 20;43(7):e47–e47.

16. Fischer DS, Theis FJ, Yosef N. Impulse model-based differential expression analysis of time course sequencing data. Nucleic Acids Res. 2018 Nov 16;46(20):e119.

17. McDowell IC, Manandhar D, Vockley CM, Schmid AK, Reddy TE, Engelhardt BE. Clustering gene expression time series data using an infinite Gaussian process mixture model. Nie Q, editor. PLOS Comput Biol. 2018 Jan 16;14(1):e1005896.
